# Segregation of an *MSH1* RNAi transgene produces heritable non-genetic memory in association with methylome reprogramming

**DOI:** 10.1038/s41467-020-16036-8

**Published:** 2020-05-05

**Authors:** Xiaodong Yang, Robersy Sanchez, Hardik Kundariya, Tom Maher, Isaac Dopp, Rosemary Schwegel, Kamaldeep Virdi, Michael J. Axtell, Sally A. Mackenzie

**Affiliations:** 1 Departments of Biology and Plant Science, The Pennsylvania State University, University Park, PA USA; 2 Department of Agronomy and Horticulture, University of Nebraska, Lincoln, NE USA; 3 Department of Biology, The Pennsylvania State University, University Park, PA USA

**Keywords:** Cellular signalling networks, DNA methylation, Plant genetics

## Abstract

MSH1 is a plant-specific protein. RNAi suppression of *MSH1* results in phenotype variability for developmental and stress response pathways. Segregation of the RNAi transgene produces non-genetic *msh1* ‘memory’ with multi-generational inheritance. First-generation memory versus non-memory comparison, and six-generation inheritance studies, identifies gene-associated, heritable methylation repatterning. Genome-wide methylome analysis integrated with RNAseq and network-based enrichment studies identifies altered circadian clock networks, and phytohormone and stress response pathways that intersect with circadian control. A total of 373 differentially methylated loci comprising these networks are sufficient to discriminate memory from nonmemory full sibs. Methylation inhibitor 5-azacytidine diminishes the differences between memory and wild type for growth, gene expression and methylation patterning. The *msh1* reprogramming is dependent on functional *HISTONE DEACETYLASE 6* and methyltransferase *MET1*, and transition to memory requires the RNA-directed DNA methylation pathway. This system of phenotypic plasticity may serve as a potent model for defining accelerated plant adaptation during environmental change.

## Abstract (web summary)

Segregation of an MSH1 RNAi transgene produces non-genetic memory that displays transgenerational inheritance in Arabidopsis. Here, the authors compare memory and non-memory full-sib progenies to show the involvement of DNA methylation reprogramming, involving the RdDM pathway, in transition to a heritable memory state.

## Introduction

Plants possess an extensive circuitry for environmental response. Short-term stress conditions during plant growth are managed through transcriptional changes triggered as short-term survival responses. The plant response primes the system to mount a more rapid and robust action upon subsequent stress exposure^1^. This acclimation, conditioned as short-term memory, involves chromatin changes surrounding key response loci^2^. However, mechanisms of phenotypic plasticity and the extent to which plants undergo transgenerational memory following chronic stress is less clear. Evolution of plant seed dispersal mechanisms were likely accompanied by mechanisms to deal with dramatic changes in environment as species invaded new niches. While examples of transgenerational, epigenetic stress memory are implied in ecological contexts^3^, reproducible induction of this type of plant behavior has not been documented.

Most plant chromatin changes are reprogrammed each generation, with the apparent exception of cytosine methylation, where some proportion of parental patterns are inherited through meiosis^4^. Genome-wide methylome analysis, therefore, provides one avenue for investigating transgenerational epigenetic behavior. In plants, cytosine methylation is generally in three contexts, CG, CHG and CHH (H = C, A or T), with CG more prominent within gene-body regions^5^. Cytosine methylation in plants is important in transposable element silencing, seed development, flower development^6^, fruit ripening^7^, stress response and formation of heritable memory^8,9^. However, association of CG gene-body methylation with changes in gene expression remains in question^10^. There exist ample data associating chromatin behavior with plant response to environmental changes^11^, but affiliation of DNA methylation with these effects, or their inheritance, remains inconclusive^9,12^.

The system we investigate involves disruption of *MUTS HOMOLOG 1* (*MSH1*), a plant-specific gene that participates in organelle genome stability^13,14^. Plastid depletion of MSH1 conditions broadly pleiotropic, variable phenotypes^15^, and altered expression of developmental and stress response pathways^16^. The *msh1* phenotype can be induced by MSH1 RNAi knockdown^17^. Subsequent null segregation of the RNAi transgene results in “memory” plants restored for *MSH1* expression but delayed in flowering, growth, and maturity transition^15,17^. Selected null segregants, termed *msh1* memory lines, display full penetrance and inheritance of the altered phenotype, as we report here. Most striking is the finding that derived memory lines, when crossed or grafted to wild-type isolines, produce progeny lineages enhanced in growth vigor and resilience^17^.

In this report, we show that *MSH1* mutation results in changes that are *HDA6*-dependent. The derived *msh1* heritable memory state, when contrasted with nonmemory full-sib progeny, reveals methylome repatterning that can be discriminated within 373 loci. These loci reside in pathways controlling circadian rhythm, auxin response, phytohormone signaling and RNA spliceosome processes and the heritable repatterning is influenced by RNA-directed DNA methylation. These results are compelling evidence for the epigenomic influence of *MSH1* disruption on plant growth and environmental response.

## Results

### The memory line phenotype

**Fig. 1:**
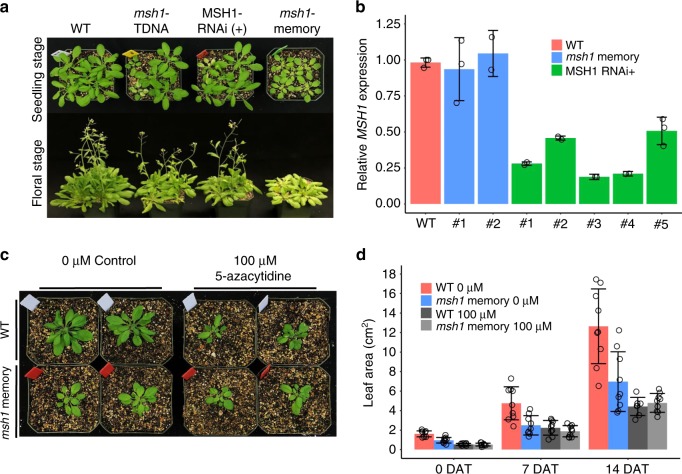
*MSH1* disruption produces transgenerational memory. **a** Phenotypic range in MSH1-derived developmental reprogramming, with *msh1* memory plants uniformly reduced in growth rate, delayed flowering and pale leaves. Seedling stage photo at 4 weeks and floral stage at 6 weeks. **b** Relative *MSH1* expression levels in *msh1* memory and MSH1− RNAi line. Each column represents one individual plant, error bars represent mean ± SD; each circle represents independent result from RTPCR experiment. **c** Phenotype of *msh1* memory line after methylation inhibitor 5-azacytidine treatment. The *msh1* memory plants were grown on MS medium with 0 or 100 µM 5-azacytidine for 10 days, then transplanted to soil; photo at 2 weeks after transplanting. **d** Leaf area measurements of wild type (WT) and *msh1* memory plants after 5-azacytidine treatment. Each circle represents data from a single plant, bars represent means ± SD, with nine plants in each population (*n* = 9). Data represent one experiment of three replicates. DAT days after transplanting. Source data underlying Fig. 1b, d are provided as a Source Data file.

In Arabidopsis lines silenced for *MSH1*, segregation of the *MSH1*-RNAi transgene produces heritable phenotype change in ca. 20% of the resulting transgene-null progeny (Fig. 1). The *msh1* memory phenotype is more uniform than that of *msh1* mutants derived from point mutation, T-DNA mutation or RNAi suppression^15,17^ (Fig. 1a; Supplementary Fig. 1a). Memory lines show normal *MSH1* transcript levels (Fig. 1b), but 100% penetrance and heritability of the altered phenotype in self-crossed generations. Over 3000 RNAi-null memory line progeny under greenhouse conditions were uniformly altered in growth, producing neither visible reversion to wild type nor intensified *msh1* phenotypes (Supplementary Fig. 1a). We postulate that memory lines maintain the heritable phenotype by virtue of epigenomic modifications.

As initial test of cytosine methylation influence on the *msh1* memory phenotype, the methylation inhibitor 5-azacytidine was applied to these plants. Germination of memory line and isogenic Col-0 wild-type seeds on media with 100 µM 5-azacytidine alleviated early phenotype differences between the two lines, resulting in similar growth rates (Fig. 1c; Supplementary Fig. 1b). Transfer of treated seedlings to potting media to assess later growth revealed similar phenotypes in wild-type and memory lines following treatment (Fig. 1d). These observations suggest the possibility of cytosine methylation influence on the memory phenotype.

### Association of *msh1* memory with cytosine methylation

To discriminate potential *msh1*-associated differential methylation from background variation, we implemented two revisions to standard analysis methods, the first biological and the second methodological. Self-crossing of the *MSH1*-RNAi suppression line produces transgene-positive and transgene-null progeny, with ca. 20% of transgene-null progeny displaying the memory phenotype and the remainder appearing unchanged and designated “non-memory”^17^. We included these nonmemory progeny in our analysis. Self-crossing an MSH1-RNAi transgenic (hemizygous) individual for a progeny population of 233 plants produced 35 transgene-nulls. Of these, seven (20%) displayed the memory phenotype and 28 appeared wild type (nonmemory) in phenotype.

**Fig. 2:**
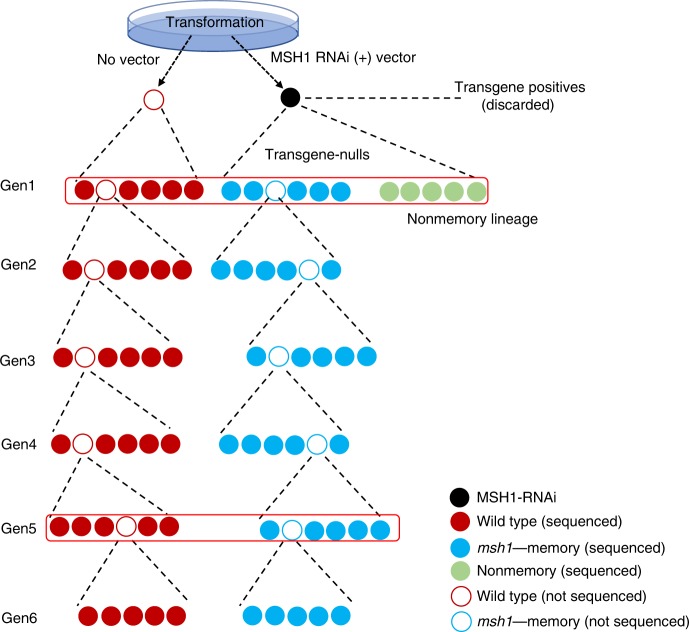
Transgenerational *msh1* memory line pedigree and sample collection strategy. The *msh1* memory lineage was developed by selecting transgene-null progeny from segregation of the *MSH1*-RNAi transgene, maintained through six generations in parallel to a wild-type control (through same transformation procedure) for each generation. Five plants from each generation (represented as solid circles) were processed for bisulfite sequencing, and five plants from wild type (WT), *msh1* memory (MM) and nonmemory (NM) generation 1 and WT and MM generation 5 were processed for RNAseq at the same time (red outline). Rosette leaf tissues were collected at bolting stage.

Memory and nonmemory full-sib progeny from one parent can be expected to share a majority of methylation patterning, revealing putative phenotype-associated methylome effects. A transgenerational memory dataset was established where the first-generation memory line was comprised of six full-sib individuals, with one used to derive the next generation by self-crossing, a process carried forward for six generations (Fig. 2). In this design, memory-nonmemory methylation differences were cross-referenced against memory-associated changes heritable over six generations to provide robust discrimination of phenotype-linked methylation variation.

Changes in genome-wide cytosine methylation were evident in *msh1* memory and nonmemory lines relative to wild type by comparing genome-wide methylation levels, confirming epigenomic activity (Supplementary Fig. 2). These data allowed assessment of transgenerational methylation fluctuation. Overall methylation level was higher among wild-type individuals than memory in all six generations. Relying on high data quality (Supplementary Data 1), rigorous experimental design and large population sizes (30 samples each for wild type and *msh1* memory, Fig. 2), we estimated probability distributions of methylation signal (divergence of methylation levels) in wild type and *msh1* memory populations with a recently published signal detection-machine-learning procedure (implemented in the R package Methyl-IT)^18^. By this means, we discriminated methylation signal induced by *msh1* from natural spontaneous variation in control samples at each methylation site. We defined a differentially methylated position (DMP) as a cytosine position with high probability to be altered in methylation by *msh1* effect, distinct from spontaneous variation in the control population. Methyl-IT analysis was previously shown to achieve excellent discrimination power^18^, so we tested this procedure for discrimination power in the *msh1* memory dataset. DMPs for CG context derived in the Gen1 nonmemory (NM) versus memory (MM) comparison were used to test four DMP identification methods, Fisher’s exact test (used by methylKit), Wald Test (used by DSS), root-mean-square test (used by methylpy) and signal detection-machine-learning method (used by Methyl-IT), for classification performance with similar methylation level difference (>0.25) and *p* value (<0.05) cutoff (Supplementary Fig. 3).

The discrimination power or accuracy of DMP calling for each method is evaluated based on performance of classifier models built on DMPs identified by each method. Supplementary Fig. 3b, c shows the signal detection-machine-learning approach to provide the best overall DMP calling performance among the four methods, with second largest number of DMPs identified, highest accuracy, sensitivity, and specificity and lowest FDR.

Applying a similar procedure to all six-generation datasets showed DMPs from all memory vs. nonmemory and WT vs. memory comparisons at false discovery rate (FDR) < 0.05 and accuracy > 90% (Supplementary Table 1). Downstream analysis with identified DMPs used the signal detection procedure (Supplementary Fig. 3a). Examining the probability density distribution of gene-body methylation divergence, the genome-wide patterning change in memory Gen6 more closely resembled nonmemory, unlike memory Gen1 to Gen5 datasets (Supplementary Fig. 4). This observation implies that Gen6 is partially transitioning to a nonmemory-like state.

**Fig. 3:**
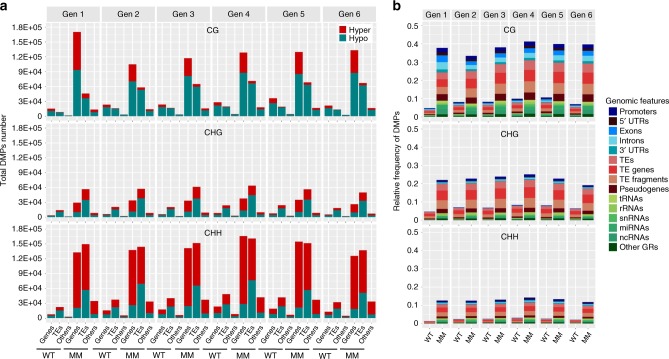
DMP counts and genomic distribution in *msh1* memory line. **a** Total hyper- and hypomethylation DMP counts in CG, CHG, CHH context embedded in genic regions (gene coding plus 1 kb upstream to start codon, 1 kb downstream to stop codon), TE-related regions (including TEs, TE genes, TE fragments and pseudogenes) and others (including tRNAs, rRNAs, snRNAs, miRNAs, ncRNAs). DMPs were defined as hyper if the site methylation difference in comparisons of each individual to the average of reference plants (the centroid of the wild type group) is greater than 0 and defined as hypo if less than 0. **b** The relative frequency of DMPs in genic regions (blue shades), TE-related regions (in red shades) and others (in green shades). The average of five wild-type plants (the centroid of the wild-type group) was used as a reference for each generation. The relative frequency of DMPs in each genomic feature was estimated as the number of DMPs divided by the number of total genomic cytosine positions in each genomic feature. Each individual’s DMP number and frequency is computed separately and the group mean for WT (wild type) and MM (*msh1* memory) are presented. Notice that DMPs are detected within wild-type samples due to spontaneous variation, distinct from treatment-induced, is detectable within our system. Source data underlying Fig. 3a, b are provided as a Source Data file.

**Fig. 4:**
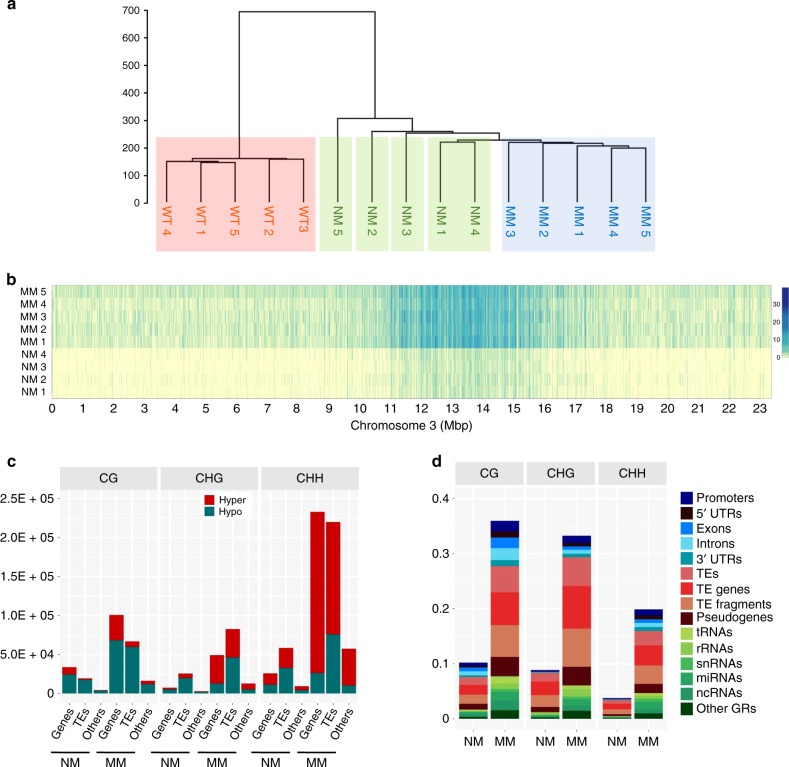
High-resolution discrimination of memory and nonmemory methylome features. **a** Hierarchical clustering of first-generation wild-type, memory, and nonmemory lines. Individuals were represented as vectors of the mean of Hellinger divergences (HD) at DMP positions within 2-kb nonoverlapping genomic regions. The hierarchical clustering was built using Ward agglomeration method. The Hellinger divergence (HD) was computed by using the centroid of five wild-type samples (HD formula is listed in Sanchez et al.^18^). **b** Heatmap showing the sum of absolute methylation difference for the direct memory vs. nonmemory comparison. The absolute value of the difference between methylation levels from control centroid (average of four control plants) and each individual (five memory plants, four nonmemory plants) at each differentially methylated cytosine site was used to build the heatmap. Each bin represents sum of methylation level difference in 2 kb. Scale bars represent the sum of absolute methylation level difference for a 2-kb interval. **c** Total hyper- and hypo-DMP counts in the NM (nonmemory) and MM (*msh1* memory) comparison. Each context, CG, CHG, CHH, was assigned to genic regions (coding region plus 1 kb upstream to start codon, 1 kb downstream to stop codon), TE-related regions (including TEs, TE genes, TE fragments and pseudogenes) and others (including tRNAs, rRNAs, snRNAs, miRNAs, ncRNAs). DMPs were defined as hyper if site methylation difference in the comparison of each individual to the control centroid was greater than 0 and defined as hypo if less than 0. **d** The relative frequency of DMPs in the nonmemory (NM) and memory (MM) comparison. DMPs are assigned to genic region (blue shades), TE-related region (red shades) and others (green shades). The centroid of the nonmemory group was used as a reference. The relative frequency of DMPs in each genomic feature was estimated as the number of DMPs divided by the number of total genomic cytosine positions in each genomic feature. Source data underlying Fig. 4c, d are provided as a Source Data file.

Identified memory DMPs showed a slightly greater trend toward hypomethylation for CG and CHG context, and increased hypermethylation for CHH, with low generational variability (Fig. 3a). The greatest proportion of DMP variation was observed within transposable element (TE) regions in all methylation contexts, with CG-associated DMPs predominant within genic regions (Fig. 3b). This repatterning includes a shifting of methylation from CG to non-CG sites in the first generation of memory individuals relative to nonmemory individuals (Supplementary Fig. 2). To understand the divergence of methylation within these three populations, we identified DMPs in all three populations and conducted hierarchical cluster analysis. With six clades detected, wild-type individuals formed one distant clade, as expected. The memory lines formed a second clade, while nonmemory individuals formed four clades, suggesting that nonmemory comprises a transition state (Fig. 4a).

Comparison of memory and nonmemory individuals revealed significant difference between the two states (Fig. 4b). Each lane within the heatmap shows a full-sib individual selected on phenotype alone, evidence of a relationship between differential methylation behavior and memory expression. DMP distribution in the nonmemory vs. memory direct comparison showed similar features to the WT vs. memory comparison, with more hypo- than hypermethylation changes in CG context (Fig. 4c), and CG-associated DMPs within genic regions at higher density (Fig. 4d).

We applied generalized linear regression analysis (GLM) to test significance of the difference between group DMP counts (WT vs. memory, nonmemory vs. memory) at genic regions (gene body plus 1 kb up- and downstream). Genes with statistically significant difference in DMP counts in memory vs. nonmemory or WT were defined as differentially methylated genes (DMGs). Relying on the robust dataset available (6 generations, 65 samples and 4-Gb reads per sample) and two additional biological filters, network enrichment analysis and heritability, to assess the nature of memory-related genes, we implemented an intermediate stringency cutoff to enhance the gene network resolution power of potential DMGs. This approach identified 6925, 5148, 5603, 7231, 7704, and 6050 DMGs for gen1–gen6, respectively, and 7130 DMGs for the nonmemory vs. memory comparison. Designation as a DMG indicates presence of a substantial number of DMPs with significant methylation level difference (Supplementary Fig. 5), reflecting high probability of methylation change in the memory plants assayed. All DMGs are not assumed to associate with memory phenotype.

**Fig. 5:**
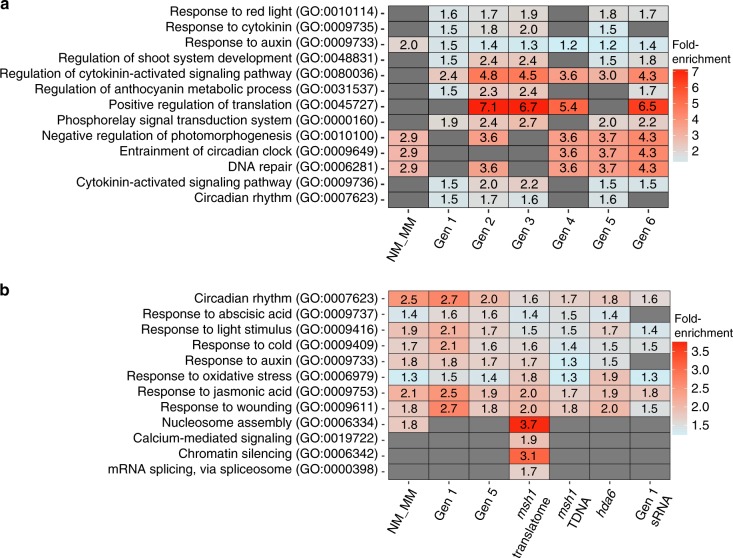
Statistically significant overenrichment of gene networks. **a** Networks identified by NEAT (Network Enrichment Analysis Test) for the DMGs identified in *msh1* memory line by MethylIT. The full list of DMGs for each generation of the memory line is listed in Supplementary Data 2. **b** Functional enrichment analysis of DEGs in *msh1* memory line, *msh1* mutant translatome and *msh1* T-DNA mutant. GO biological process enrichment categories above the cutoff, FDR < 0.01, are shown. DAVID GO was used to conduct the analysis^76^. The *msh1* T-DNA mutant expression data were generated and reported in Shao et al.^16^. The *msh1* mutant translatome refers to the MSH1 cell type-specific TRAPseq dataset that was reported in Beltrán et al.^20^. The *hda6* transcriptome data were taken from Yu et al.^27^ and reanalyzed using our pipeline. For Gen1 sRNA, genes within ±1 kb of differentially expressed sRNA clusters were used for the GO analysis. Source data for generational DMGs are provided as a Source Data file.

To screen the DMG datasets for phenotype-associated DMGs, we used the additional biological filters of network enrichment analysis and heritability. Network-based enrichment analysis identified pathways shared by at least four of the seven DMG datasets tested, most predominant being circadian clock, stress response and phytohormone signaling networks (Fig. 5a, Supplementary Data 2). We also conducted network enrichment analysis with 2637 DMGs that were obtained for the memory–nonmemory comparison under more stringent filtering conditions. Results from this parallel analysis showed a similar enriched network profile to that obtained with the 7130 DMGs, including circadian rhythm and hormone signaling pathways, but with fewer individual genes identified in each network (Supplementary Fig. 6). These results imply that the enriched network profile identified with intermediate filter stringency is robust and biologically meaningful.

A comparative analysis within wild type alone permitted assessment of background methylome variation identifiable as DMGs. Analysis of five wild-type samples from Gen3 (samples 1, 2, 3 as reference and control, with 4, 5 as treatment) revealed 1854 DMGs for variation in cold response, photomorphogenesis, meristem and plant development, response to sucrose, and photoperiodism as identifiable pathways (Supplementary Data 3). These DMGs are characteristic of variation for transition to flowering, consistent with expectations for plants harvested at bolting stage^19^. The wild-type DMGs from Gen3 data produced considerable overlap (1374, 74%) with Gen3 memory DMGs, also harvested at bolting stage. Therefore, the wild-type DMGs were subtracted from the Gen3 memory dataset to enrich for memory-specific effects and reduce influence of developmental signal. This subtracted dataset of 4231 Gen3 memory DMGs showed greater enrichment in circadian rhythm, auxin response, cytokinin signaling, and stress response pathways (Supplementary Data 4).

### Memory methylation changes intersect with gene expression

To assay gene expression changes in *msh1* memory, two generations were selected, with Gen1 (WT, MM and NM plants) representing the *msh1* memory-initiation stage and Gen5 (WT, MM) representing an advanced stage. We identified 5045 differentially expressed genes (DEGs) in the Gen1 nonmemory vs. memory comparison, 4509 in Gen1 WT vs. memory comparison and 5777 DEGs for Gen5 WT vs. memory (Supplementary Data 5–7). Gene expression datasets in nonmemory and wild-type comparisons were highly similar (Supplementary Fig. 7a), indicating that methylation changes shared between nonmemory and memory lines, when compared against wild type, do not detectably impact phenotype. Therefore, we used memory vs. nonmemory comparisons to discriminate phenotype-associated methylation effects.

Two important limitations exist for comparing gene expression with methylation variation. Because genes function in networks, only a portion of associated regulators may undergo methylation changes. Also, spatiotemporally regulated gene expression may go undetected in pooled tissue sampling. To partially offset these limitations, we conducted gene enrichment analysis with expression datasets to identify overlaps with methylation network data (Fig. 5b). We also included gene expression data derived from targeted purification of polysomal mRNA (TRAP-Seq) analysis of changes within *MSH1*-expressing cells in wild type vs. *msh1* ^20^.

Several important observations emerged from this analysis. Four gene pathways were detected exclusively within data from *MSH1*-expressing cells and not generally: nucleosome assembly, chromatin silencing, calcium-mediated signaling and mRNA splicing via spliceosome (Fig. 5b). Arising distinctly in *MSH1*-expressing cells, we assume that these changes represent early and local response to *MSH1* suppression and precede memory induction. Comparison of memory vs. nonmemory identified gene networks for circadian clock, light response, and phytohormone response to stress. Importantly, pathways identified in both methylation and gene expression datasets are known to be highly interconnected (Fig. 5) and function as plant environmental sensing and response mechanisms^21,22^.

As an alternative means of assessing the relationship of gene expression to methylation repatterning in the memory state, we carried out a correlation analysis of DNA methylation divergence (MD) and gene expression divergence on 20,022 genes (total of genes with methylation divergence > 0) in gen1 *msh1* memory plants. A linear statistical association between and MD was confirmed with application of a linear-by-linear association test (*p* value ≪ 0.0001). The Spearman’s *ρ* = −0.166 for upregulated genes and *ρ* = −0.17 for downregulated genes suggests that gene expression and methylation processes are not independent in Gen1 memory line (Supplementary Fig. 8a, b).

We further investigated the impact of methylation change location and direction on gene expression. Using 821 genes that were both DMGs and DEGs in Gen1 *msh1* memory, we identified significant difference between CG gene-body hypo- and hypermethylated DMG expression levels, with higher CG gene-body methylation associated with lower gene expression (Supplementary Fig. 8c). A significant difference was also observed between CHH promoter region hypo- and hypermethylated DMG expression level, with higher CHH methylation in promoters associated with lower gene expression (Supplementary Fig. 8d).

### Altered circadian clock network within the memory line

**Fig. 6:**
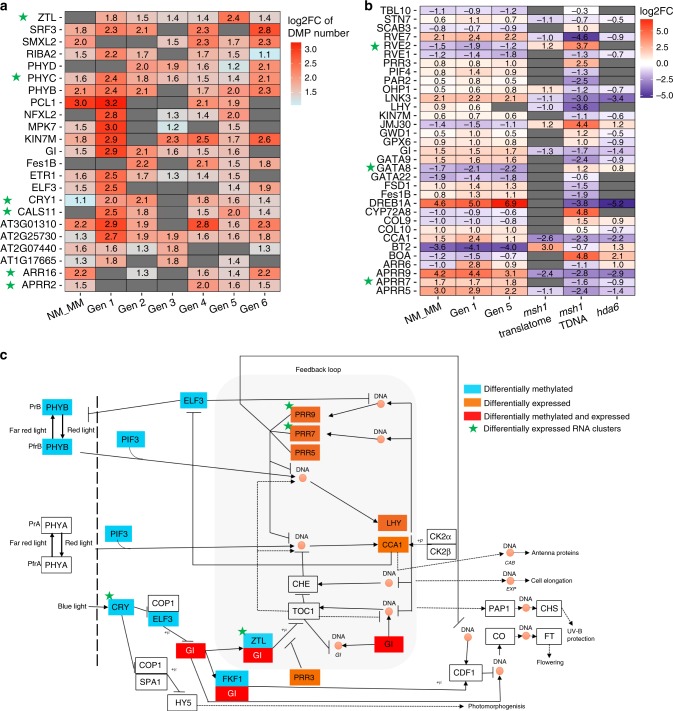
Circadian rhythm network genes are both differentially expressed and methylated. **a** DMGs for the circadian rhythm (GO:0007623) network in *msh1* memory line. Only genes identified as DMGs in at least four out of seven comparisons (Gen1 to Gen6 WT vs. MM and Gen1 NM vs. MM) are presented. Scale represents the log2 Fold change of DMP number at specific genes in each comparison. **b** DEGs from the circadian rhythm (GO:0007623) network in *msh1* memory line and *msh1* mutant. Only genes identified as DEGs in at least four out of five comparisons (Gen1 WT vs. MM, Gen1 NM vs. MM, Gen5 WT vs. MM, WT vs. *msh1* translatome (TRAPseq) and WT vs. *msh1* TDNA mutant) are presented. **c** DMGs and DEGs presented in panels (**a**) and (**b**) are positioned in the circadian clock network. The network is adopted from the KEGG database (Circadian rhythm—plant—*Arabidopsis thaliana*). The *hda6* transcriptome data were taken from Yu et al.^27^ and reanalyzed using our pipeline. Genes associated with differentially expressed sRNA clusters were highlighted with a green star. Source data for generational DMGs are provided as a Source Data file.

To investigate the relationship between differential methylation and gene expression in *msh1* memory, we looked at individual genes within three prominent networks identified by our analysis: circadian rhythm, response to auxin and spliceosome function. In the circadian rhythm gene network, at least 23 genes were identified as differentially methylated in memory vs. nonmemory and/or heritably altered in at least four of the six generations (Fig. 6a). We interpreted occasional discrepancies in representation (e.g. presence in generations 2 and 4, but not 3) as stochastic fluctuation around the significance threshold.

Circadian clock-related DEGs were examined in Gen1 and Gen5, a much greater number than DMGs, with only modest overlap between the two (Fig. 6a, b). Integration of DMG and DEG datasets showed a trend of differential methylation within genes functioning upstream in the pathway (Fig. 6c), and a significant proportion (37.3–40.8%) of the 1982 genes that interact with CCA1/TOC1 differentially expressed in *msh1* memory (Supplementary Fig. 7b), reflecting sizeable enrichment. Networks regulated by circadian clock functions and differentially expressed in the memory line included starch metabolism and responses to ethylene, ABA, and cold stress (Supplementary Fig. 7c–e).

Evaluation of circadian clock behavior in *msh1* memory by tracking gene expression levels of four selected circadian core genes, *TOC1, LHY, CCA1* and *GI*, under 24-h light and alternating light–dark conditions showed significant and similar changes in each. Memory lines were altered in oscillation amplitude, with little phase change (Supplementary Fig. 9). These patterns resemble those created by the *rve 4, 6, 8* triple mutant, where *REVEILLE8* (*RVE8*) and its homologs *RVE4* and *RVE6* function to regulate growth by controlling cell size^23^. Memory plants display significantly altered days to bolting, leaf area, and chlorophyll content in short vs. long days, relative to isogenic wild type (Supplementary Fig. 10), typical of many circadian clock mutants^24–26^.

### Altered auxin response and RNA splicing networks in memory

Within the auxin response network, over 49 genes displayed differential methylation in memory vs. nonmemory and/or at least four generations (Supplementary Fig. 11a). Among them were crucial auxin response genes *GH3.1* and *GH3.9*, which function in auxin conjugation, *ABCB 19(PGP19)* and *ABCB1(PGP1)* encoding key auxin transport proteins and *ARF1, ARF6, ARF8*, which encode central auxin transcription factors (Supplementary Fig. 11c). Numerous auxin-associated DEGs, including genes critical for auxin synthesis (*IAA4, IAA18* and *IAA29*) and SAUR family genes (auxin early response factors), were identified. Auxin network genes including *AXR4* and *ATHB-8* were present in both DMG and DEG datasets (Supplementary Fig. 11a, b).

A third pronounced pathway identified within the *msh1* memory DMG dataset involved components of spliceosome-mediated RNA splicing. At least 42 loci in this pathway were identified as differentially methylated in comparisons of memory with nonmemory and/or at least four generations (Supplementary Fig. 12a). From the analysis of *msh1* differential expression, this network emerged in TRAP-Seq data for MSH1 cell-specific expression (Supplementary Fig. 12b). These observations suggest that changes in alternative splicing pathways occur early in *MSH1* suppression outcomes^20^. A surprisingly large number of components in this network are influenced in *msh1* (Supplementary Fig. 12c), which may contribute, in part, to the dramatic and programmed changes in gene expression^16,17^.

The experiment was designed to define memory-associated genes as those displaying methylation differences in the memory–nonmemory comparison that are retained through six consecutive generations. These criteria narrowed the original 5000–7500 DMGs to a total of 954 (Supplementary Data 8). Consistent with our other analyses, this refined dataset showed overenrichment for 21 networks involved in phytohormone and stress response, circadian rhythm, and regulation of transcription (Supplementary Data 9). Memory changes in transcriptional regulation identified 32 loci known to participate in auxin response, abiotic and biotic stress response, DNA and chromatin modifications, and circadian clock (Supplementary Data 9).

### Confirming gene methylation repatterning in memory

To understand gene-associated repatterning within the memory line, we plotted DMPs by cytosine context in the selected 954 loci with heritable, memory-associated changes. Supplementary Fig. 13 shows repatterning predominantly by hypermethylation within CHH context and milder CG hypomethylation to distinguish memory from nonmemory and wild type. Despite the genome-wide methylome shift toward nonmemory in Gen6 (Supplementary Fig. 4), the DMP distribution pattern among 954 heritable Gen6 DMGs was very similar to other generations (Supplementary Fig. 13).

We elucidated methylation repatterning effects by examining individual loci. Expression of 18 circadian-, hormone-, and spliceosome-related genes was confirmed by quantitative real-time PCR and association with *msh1* memory phenotype (Supplementary Fig. 14). To confirm DMP calling accuracy by Methyl-IT, we selected a promoter region within *XTH16* (AT3G23730) with substantial DMP density and hypermethylation in all three contexts in each generation of *msh1* memory (Supplementary Fig. 15a). Targeted BS-PCR confirmed DMP calling for both nonmemory vs. memory comparison (Gen1) and wild type vs. memory (Gen6).

**Fig. 7:**
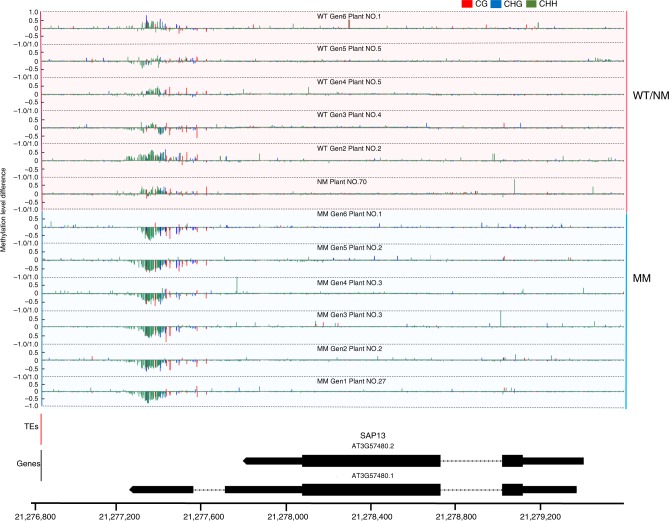
Methylation changes at DMG loci. Single cytosine methylation level changes in the *msh1* memory line (MM) *SAP13* (AT3G57480) locus. Methylation level difference at each cytosine is computed by (mC/(mC + uC))_each individual_ − (mC/(mC + uC)_average of all reference plants_, with mC denoting methylated cytosine and uC denoting unmethylated cytosine. For gen1, nonmemory plants were used as reference. For gen2 to gen6, wild-type plants were used as the reference for each generation. Only one plant from each generation was selected as representative; the pattern will be slightly different for different individuals due to fluctuation in methylation. Integrated Genome Browser (version 9.0.2) was used to generate figure.

Gene-associated methylation changes were partitioned into gene-body vs. TE-associated and displayed by cytosine context. Figure 7 shows an example of high-density gene methylation changes (mainly CHH hypomethylation in this case) in the memory line with no TE influence. The gene shown, *STRESS-ASSOCIATED PROTEIN 13* (*SAP13*), is responsive to abiotic stress and is differentially expressed in the memory line. In TE-associated loci, genes adjacent to, or containing, transposable element sequences generally displayed high-density DMPs within the TE sequence as well as promoter or gene-body of the adjacent locus.

*ATMEKK1* encodes a member of the MAPK/ERK kinase kinase family that mediates stress signaling. The gene is altered in expression and methylation in the memory line, detectable as mainly CG hypomethylation in the gene-body (Supplementary Fig. 16). *ARF8*, encoding an auxin responsive protein, shows evidence of TE-associated gene-body methylation changes. These differences in methylation (CG hypomethylation in gene-body, CHH hypermethylation in promoter region) persist six generations (Supplementary Fig. 17). Similarly, *GIGANTEA* (*GI)* is an important component of the circadian clock core network and is heritably altered in expression in the memory line. The altered methylation domain within this gene, outlined in black, was detected in all six generations (Supplementary Fig. 18a). The subtle differential methylation signal near the 3′ end of GI, mainly CG context, was confirmed by targeted BS-PCR sequencing (Supplementary Fig. 18b).

Significant CHH repatterning in memory prompted us to develop sRNA sequence data for memory and wild-type lines. Samples were derived from Gen1 memory and corresponding wild-type plants. Screening differential 24nt sRNAs from these datasets identified 2999 differentially expressed sRNA clusters and 2847 genes within ±1 kb of these clusters (Supplementary Data 10), representing similar pathways as in memory line methylome analysis (Fig. 6b). In all, 913 DMGs from memory Gen1 and 205 DMGs from the 954 heritable DMGs were located within ±1 kb of the sRNA clusters (Supplementary Data 8, 10). Among 373 DMGs in four prominent networks (Supplementary Data 11), 57 were colocalized with differentially expressed sRNA clusters, and individual gene examples are highlighted with a green star in Fig. 6 (circadian rhythm network) and Supplementary Fig. 11 (auxin response network). The location of differentially expressed sRNA clusters within heritable DMGs is pinpointed in *MEKK1* (Supplementary Fig. 16) and *ARF8* (Supplementary Fig. 17). These observations further support a conclusion of nonstochastic repatterning and likely participation of the RdDM pathway in the maintenance of memory.

Principal component and linear discriminant analyses were used to assess the 954-DMG *msh1* memory dataset in Gen1 memory, Gen2-6 memory, nonmemory and wild-type lines (Supplementary Fig. 19a). The nonmemory dataset was separable from memory in Gen1, consistent with distinct repatterning of memory relative to nonmemory sibs, and subsequent generation datasets comprised a distinct and cohesive transition. These relationships were retained when analysis was focused on a subset of only 373 DMGs in four prominent networks, circadian rhythm, response to auxin, RNA spliceosome and phytohormone signal transduction, indicating that changes within these four pathways are sufficient to discriminate the memory state from nonmemory (Supplementary Fig. 19b).

A similar approach was used to investigate the relationships of gene expression (Supplementary Fig. 19c, d) and DMG (Supplementary Fig. 19e, f) datasets derived from memory lines before and after 5-azacytidine treatment. In both gene expression and methylation repatterning, memory and isogenic wild-type datasets were distinct. However, following 5-azacytidine treatment, the memory and wild-type samples clustered, consistent with the hypothesis that *msh1* memory methylation effects are largely obviated by 5-azacytidine treatment. Further elaboration of these data in Supplementary Fig. 20 shows quantitative gene expression assays for 16 selected memory DEG loci before and after 5-azacytidine treatment.

### Genetic control of the *msh1* effect

**Fig. 8:**
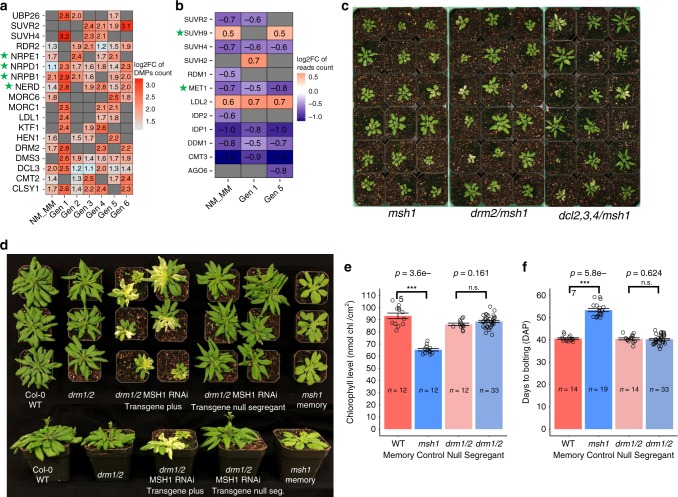
Genetic control of the *msh1* effect. Methylation/demethylation and silencing pathway component methylation (**a**) and expression changes (**b**) in *msh1* memory. Genes associated with differentially expressed sRNA clusters were highlighted with a green star. **c** Double mutants of *msh1* with components of the RdDM pathway. **d** Embryo lethality in the *msh1/hda6* double mutant. Methylation/demethylation and silencing pathway component gene list was taken from Matzke et al.^5^. **d** Representative plants of WT, *drm1/2*, *drm1/2* MSH1 RNAi (+), *drm1/2* MSH1 RNAi transgene null segregant and *msh1* memory (12 h day length, 6-week-old plants). **e** Chlorophyll measurements, and **f** days to bolting in wild type (WT), *msh1* memory, *drm1/2 and drm1/2* MSH1 RNAi transgene null segregant. Bars represent means ± SE, *n* represents number of plants in each population. One-sided Mann−Whitney test was used to compute *p* value. Significance codes: ***(*p* value < 0.001), ns not significant. Plants grown on normal soil conditions. Chlorophyll level was measured with a SPAD-502 meter 4 weeks after planting. Source data underlying Fig. 8e, f are provided as a Source Data file.

To begin to dissect components of the *msh1* epigenomic process, we examined the effect of methylation/demethylation and silencing pathway components on establishment of the *msh1* state, previously shown to involve significant changes in CG methylation^17^, and the transition to *msh1* memory, shown here to involve significant CHH repatterning. Several major RNA-directed DNA methylation (RdDM) pathway protein coding genes were differentially methylated in multiple generations of *msh1* memory, including *RDR2*, *DCL3*, *DRM2*, and *CLSY1*(Fig. 8a). Methylation components *MET1* and *CMT3* were downregulated, and chromatin modifiers *SUVR2* and *SUVH4* were both altered in methylation and expression (Fig. 8a, b). These observations imply that the RdDM pathway and particular methylation and chromatin modifiers participate in the MSH1 effect. We, therefore, constructed a series of *msh1* double mutants in these pathways. Figure 8c shows that double mutants between *msh1* and components of the RdDM pathway displayed a phenotype similar or identical to *msh1* alone. These data indicate that a fully intact RdDM pathway is not essential for initial *msh1* effects.

In contrast, the double-mutant *msh1/hda6* was not recoverable in segregating populations germinated on soil or nutrient media (Table 1; Supplementary Table 2). This observation indicates that histone deacetylase HDA6 activity is required for initial *msh1* reprogramming. The *hda6* mutant is altered in expression of 5738 genes^27^, of which 49% (2855) are shared with the *msh1* DEG dataset, a 75% overlap of gene ontology networks identified for the *hda6* dataset (Fig. 5b; Supplementary Data 12). In comparisons with memory, the *hda6* DEG dataset shared 34% (1948) overlap with the Gen1 memory line dataset and 44% (2583) with Gen5 memory. HDA6 is known to recruit the methyltransferase MET1 to TEs and other sites that undergo differential methylation and silencing^28–30^ and participates in regulation of pathways identified in this study^29,31–33^ (Fig. 6; Supplementary Figs. 7, 11). Expression changes in the auxin response and circadian rhythm pathways in *msh1* memory show an inverse relationship with expression changes in *hda6*, consistent with HDA6 participation in this response.

**Table 1:** Segregation analysis of *msh1*^−/−^/*hda6-7*^+/−^ F_3_ plants.

Progeny genotype	On soil		On 0.5 M MS medium	
	Expected # of progeny	Observed # of progeny	Expected # of progeny	Observed # of progeny
*msh1*^−*/*−^*/hda6-7*^*+/+*^	103	343	23.5	77
*msh1*^−*/*−^*/hda6-7*^*+/*−^	206	69	47	17
*msh1*^−*/*−^*/hda6-7*^−*/*−^	103	0	23.5	0
Total	412	412	94	94

Double mutants of *msh1/met1*, while recovered, were markedly reduced in frequency relative to expected (Supplementary Tables 3 and 4). These data implicate the MET1−HDA6 interaction in initial *msh1* epigenomic reprogramming. Interestingly, the *MET1* promoter is overlapped by a transposon (AT5TE71740; Supplementary Fig. 21), and both the element and *MET1* promoter undergo methylation change. This change, located more than 1 kb upstream to *MET1*, was not identified as a DMG in our datasets. The data show that initial stress-derived *msh1* reprogramming depends on *HDA6* and *MET1*.

To investigate *msh1* memory transition, with increased CHH variation, we introduced the MSH1-RNAi construct to a *drm1/2* double mutant to attempt memory induction. DRM1 and DRM2 methyltransferases maintain CHH methylation in the RdDM pathway^34^. Subsequent segregation of the RNAi transgene permitted testing for evidence of memory in progeny. Of 547 progeny from a *drm1/2* MSH1-RNAi hemizygous plant, we identified 170 transgene-nulls and no evidence of *msh1* memory phenotype based on growth rate, chlorophyll content and flowering time (Fig. 8d, e). We would predict ca. 20% (34 plants) to display *msh1* memory behavior. These data, together with sRNA datasets, support RdDM participation in memory methylation repatterning.

## Discussion

*MSH1* suppression in plants leads to developmental reprogramming and expression of phenotypic plasticity^15^. The *msh1* memory comprises a distinct state that occurs in about 20% of plants that have undergone reprogramming. Characterized by reduced growth rate, altered chlorophyll content, delayed maturity transition and flowering, and enhanced stress response, *msh1* memory is unexpectedly stable, penetrant and heritable.

Comparison of memory and nonmemory progeny from a single parent revealed memory-specific methylation changes, supporting phenotype-associated methylome behavior. These data showed a marked change in memory progeny methylation repatterning (Fig. 4; Supplementary Figs. 4, 13a) with local but not globally significant change in methylation level (Supplementary Fig. 2). The observed repatterning appeared to target specific gene networks, permitting discrimination of the memory state based on 373 DMGs associated with circadian rhythm, auxin response, spliceosome functions and plant hormone signal transduction.

Memory and nonmemory were similar in methylome repatterning when compared to wild type, but clearly distinguishable with PCA + LDA and cluster analyses (Fig. 4a; Supplementary Fig. 19). Changes shared by memory and nonmemory types, and the variation observed in nonmemory individuals, suggest a continuum in reprogramming and a threshold that delimits the memory state. We assume that some of the nonmemory lines could diminish or intensify in memory methylome effects in subsequent generations (Supplementary Fig. 4). The extent to which nonmemory plants can display phenotypic plasticity under stress is not known.

It might be argued that the methylation level differences detected between memory and nonmemory were sufficiently subtle to reside within the range of natural fluctuation or epigenomic drift. But our data show that memory full-sib individuals display evidence of methylome repatterning at markedly greater magnitude than occurs by natural fluctuation, represented in wild-type and nonmemory individuals (Fig. 4). These observations reflect influences on memory methylome behavior that are more subtle than are commonly described in most plant epigenomic studies, yet readily distinguished from random epigenomic variation. This outcome provides perspective for plant methylome studies when applied to phenotypic plasticity. Whereas methylation behavior during gene and TE silencing often involves high-density, large magnitude changes, methylome repatterning in response to environmental flux may require higher resolution quantitative analysis.

Integration of DMG and DEG networks identified memory-associated plant processes. Memory phenotype involved changes in circadian rhythm, auxin response, cytokinin-related, spliceosome, and stress response pathways. These results are consistent with previous datasets investigating *msh1* mutant^16,35^ and cell-specific^20^ expression changes and imply that *msh1* memory alters central control networks within the plant.

Genome-wide methylome studies require signal-to-noise discrimination^36^. Methyl-IT DMP-based analysis identified 954 genes with differential methylation in memory vs. nonmemory comparisons, and through six generations of testing. The experiment was designed for stringent, unbiased selection of DMGs, incorporating filters for signal detection, discriminating background variation from *msh1* effect, plant phenotype association, and six-generation heritability followed by gene network association. What is striking about the resulting 954-gene dataset is its similarity, in networks identified, to each generational dataset (Supplementary Data 2 and 9). Whereas memory-associated DMG numbers varied somewhat each generation, the fundamental biological interpretation remained consistent in identifying circadian rhythm, phytohormone, spliceosome and abiotic/biotic stress response pathways in the memory state. Taken together, these observations reflect nonstochastic, programmed methylation repatterning of select gene networks.

Methyl-IT analysis produced larger DMG numbers within each dataset than would generally be identified by conventional DMR analysis. A dataset of 6925 DMGs was derived in the Gen1 memory vs. wild-type analysis. Of these, 1115 (16.1%) were retained in all subsequent generations. However, 79.4% of the DMG total was detected in at least two generations, 63.1% in at least three generations, 47.7% in at least four generations, and 32.5% in at least five generations. In contrast, simulation (selecting six gene lists with equal generational DMG numbers from a total of 27,655 Arabidopsis genes randomly) produces, among 6925 randomly selected genes, 8.0% in at least four lists, and 1.2% in at least five lists, with only four genes (0.05%) retained in all generations. Hence, the observed recurrence rate of identified DMGs throughout the generational study is significant and non random (*p* value < 10^−16^, Pearson’s chi-squared test).

We assume that intergenerational variability observed in the memory datasets reflects inherent stochasticity in the system. While we measured variability within methylome and gene expression datasets, metabolic variability likely occurs as well, invoking organellar influence. We observe a strong degree of organellar and metabolic signal in both methylome and gene expression datasets, and others have shown evidence of organellar influence on stochasticity behavior^37^. However, it is not feasible to fully distinguish contribution of organellar vs. methylome effects to the memory state.

Comparison of identified *msh1* memory DMG datasets with previously reported gene-body methylated (GbM) genes in Arabidopsis^38^ showed only ca. 34% overlap of memory DMGs with previously identified GbM loci, so that a large proportion of *msh1* memory DMGs were not previously categorized as gene-body methylated. DMGs associated with circadian rhythm, cytokinin response, abiotic and biotic stress response and alternative splicing networks were largely missing from the overlap of *msh1* DMGs with the public GbM dataset. Similar expansion of gene-associated methylation has been reported in other studies^6,39^. These observations may reflect gene-body methylation as a function of developmental staging and/or growth conditions, with *msh1* memory representing a previously undescribed plant state.

Intersection of methylation repatterning with gene expression was not pronounced on a gene-by-gene basis (70 and 104/954 DMGs were identified as DEGs in Gen1 WT vs. MM and Gen5 WT vs. MM, respectively), but produced informative outcomes with network-based enrichment. Data for circadian rhythm, auxin response and RNA splicing pathways suggested that methylation repatterning was more prominent in upstream components of these pathways. The extent that DNA methylation affects gene expression and vice versa is not known in this system, and a number of DMGs may function to re-establish local homeostasis without influencing phenotype^40^. However, we provide evidence that a relationship between methylation repatterning and gene expression is detectable within these datasets.

Memory-associated methylation repatterning is assumed to accompany, and may be consequence of, significant chromatin changes during MSH1 suppression. Altered expression in *msh1*, based on TRAPseq studies of specific cells harboring MSH1, implicate changes in histone composition and numerous chromatin modifiers as prominent early effects in the *msh1* mutant^20^.

General heritability of identified DMGs within this system suggests that a subset is targeted and fixed once memory is established. For example, the methylation maintainer *CMT2*, RdDM pathway components *DCL3*, *DRM2*, *CLSY1* (Fig. 8), circadian rhythm components *GI*, *PHYB*, *PHYC*, *PHYD*, *CRY* (Fig. 6, Supplementary Fig. 18), central auxin response genes *ARF8*, *GH3.1*, *ABCB1*, *ABCB19* (Supplementary Figs. 11, 17) and genes involved in the spliceosome complex (Supplementary Fig. 12) are candidates for this effect. These loci comprise a conservative list of *msh1* memory “core” components and represent examples of factors that could trigger a cascade in environmentally induced gene expression changes.

We detected evidence of transposable element association with heritable memory methylation behavior. Of 954 heritable memory DMGs identified, 538 (56.4%) contained at least one TE within a 1-kb distance (Supplementary Data 13). This frequency represents a significant enrichment (Fisher’s exact test, *p* value < 10^−6^) when compared to the 6936 (25.3% of 27,420 annotated) genes with at least one TE at 1-kb distance in Arabidopsis^41^. These data are consistent with numerous studies suggesting that plant TEs have evolved positionally within plant genomes to participate in gene regulation and phenotypic plasticity^40–43^.

Proximal TE-associated methylation may facilitate transgenerational heritability of methylation repatterning. One example is *MEKK1* (Supplementary Fig. 16), which is downregulated in expression in memory and shows significant differential methylation over multiple *msh1* memory generations. A TE residing upstream to this gene may account for the transgenerational gene-body methylation of the locus. The methyltransferase *MET1* is also downregulated in expression and shows heritable methylation differences in *msh1* memory, a factor that may contribute to the observed relaxation of gene expression constraints in *msh1*. The *MET1* promoter is overlapped by a TE (AT5TE71735; Supplementary Fig. 21) that appears to participate in its heritable methylation repatterning following environmental stress^44^.

The hyper-stress condition that arises with MSH1 depletion appears to be unsustainable in the absence of functional *HDA6*. This histone deacetylase has been associated with genes controlling plant circadian clock, ABA stress response, auxin signaling, brassinosteroid signaling and flowering time^45^. Histone deacetylases (HDACs) catalyze removal of acetyl groups from acetylated lysine residues in the N-termini of histone proteins that serve as epigenetic marks^45^, thus impacting chromatin compaction and gene suppression.

HDA6 interacts directly with the methyltransferase MET1, both in vitro and in vivo^29^, and disruption of *MET1* also affected viability of the *msh1* mutant state. HDA6 participates in DNA methylation; the *hda6* mutant loses CG methylation at target loci that are not surrounded by flanking DNA methylation^28^. In these loci, MET1-derived CG methylation is dependent on HDA6. The 954 heritable DMGs identified in this study are divided approximately evenly between loci that are associated with TE sequences, supplying flanking DNA methylation, and loci that appear independent of TE influence; it is possible that TE association and HDA6 both contribute to transgenerational DMG retention.

**Fig. 9:**
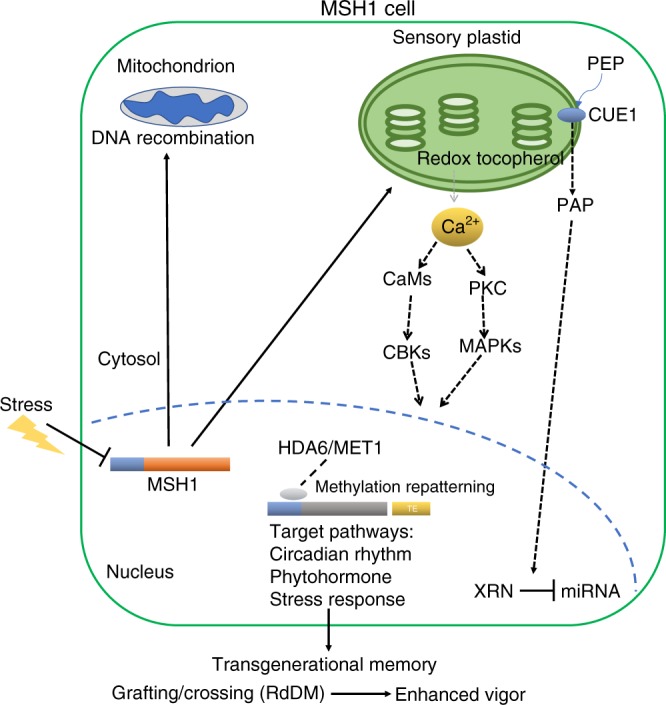
A simplified model of MSH1-associated phenotypic plasticity. Stress-associated suppression of *MSH1* expression alters conditions within the sensory plastid of epidermal and vascular parenchyma cells^20,58^. These changes involve at least two retrograde signaling pathways to the nucleus, one including redox and calcium signaling^20^ and the other tocopherol-mediated modulation of the PAP phosphonucleotide as a mediator of miRNA regulation^58,77^. Nuclear response to sensory plastid perturbation is dependent on *HDA6* and *MET1* and includes genome-wide cytosine methylation repatterning and altered expression of integrated stress response networks. Specificity factors (gray oval) have been identified that associate with, and recruit, HDA6 to target loci that participate in the *MSH1* effect^45^. The heritability of this repatterning may be influenced by proximity of TEs to the target loci and the nature of HDA6 activity. Transgenerational memory induced by *MSH1* suppression gives rise, through crossing or grafting, to progeny with markedly enhanced growth vigor and resilience phenotypes^15,17,59,60^. Gene promoters are shown as blue bars; target genes are shown by generic gray bar. Solid lines reflect data shown by our group; dashed lines reflect data published by other groups that are consistent with MSH1 data (past and present). This figure was created by the authors.

HDA6 interacts directly with proteins FLD^46^, FVE, MSI^47^, HOS1^48^ and AHL22^49^ that influence flowering time. JAZ 1, JAZ3, JAZ9, EIN3, EIL11^50^ and COI1^51^, involved in ethylene and jasmonate response, also comprise HDA6 interaction partners in environmental response. HDA6 associates with TOPLESS (TPL) and pseudo-response regulators to regulate core circadian clock loci^52^ and, together with LDL1/2, functionally associates with CCA1/LHY^53^ and TOC1^33^ clock components. These protein interactions are thought to comprise a means of site-directed targeting by HDA6, acting on pathways that are prominent in *msh1* memory. Observed lethality in the *hda6/msh1* double mutant appears consistent with the centrality-lethality rule in network hub behavior^54,55^, and implies that HDA6 serves as a vital hub within the identified networks during *msh1* changes. Previous results suggest that MSH1 depletion triggers a signal from within the sensory plastid that elicits *msh1* system-wide stress response in the plant^20^ (Fig. 9). Lethality of the *hda6/msh1* double mutant implies that programmed TE and gene-associated methylation changes dependent on HDA6 are essential to the cell’s ability to mount this stress response.

Apparent influence of *drm1/2* to suppress induction of the memory state following MSH1-RNAi transgene segregation, together with memory-associated repatterning of genic CHH methylation, suggest that RdDM-mediated methylation distinguishes memory from nonmemory in our study. HDA6 functions in association with RdDM processes^45^ and, with identification of specific memory-associated loci, it should be feasible to more fully delineate the influence of these various chromatin remodeling components in a stepwise manner.

The MSH1 phenomenon is a system distributed throughout angiosperms^15,56^. Modeling this system as a plant environmental response mechanism presumes that *MSH1* is environmentally responsive. In fact, *MSH1* steady-state transcript levels are suppressed under conditions of drought, heat^57^, excess light^14^ and cold^35^. Previous studies show that plastid depletion of MSH1 leads to local changes in epigenetic, calcium signaling, and spliceosome responses^20,58^, and the *msh1* mutant displays phenotypic variation, from nearly wild type to severely dwarfed^15^. Individuals within a mutant population are enhanced in tolerance to high light^14^, drought^58^, cold^35^ and heat^57^. Mutant plants show evidence of short-day partial perennialization, partial male sterility and altered leaf morphology^15^.

Self-crossing of an *MSH1* RNAi suppression line produces heritable memory that is similarly primed for stress but more uniform in phenotype intensity. Crossing wild type and the memory line, or presumably a plant environmentally suppressed for *MSH1* expression, produces progeny populations with markedly enhanced fitness^17,59,60^.

These behaviors resemble expectations for the evolutionary concept of diversified bet hedging, wherein an organism under chronic stress undergoes epigenetic changes to unleash phenotypic variation for survival of at least a portion of its progeny^61^. In an *MSH1* scenario, chronic stress leading to *MSH1* suppression elicits nongenetic variation within a population. Surviving progeny would be heritably sustained through programmed memory characterized by plasticity. Successful intercrossing within the emergent population would give rise to progeny enhanced in fitness. Phenotypes derived by artificial *MSH1* suppression may exaggerate what would be common to natural ecosystems, but the reproducibility of this *MSH1* effect is conspicuous across plant species tested^15,59,60^.

If *MSH1* participates in natural phenotypic plasticity, the epigenetic mechanism could have coevolved with plant seed and spore dispersal mechanisms. Transit of seed increases the probability of environmental change within a single generation, requiring rapid adaptation and cross-tolerance mechanisms. *MSH1* plasticity and heritable memory provide a bet hedging mechanism with acquired fitness, and offers approaches to agricultural improvement for variable climates. Conservation of *MSH1*^56^ across plant species and uncommon pliability of the system should permit direct modeling of this process.

## Methods

### Plant materials and growth conditions

For Arabidopsis plants used in this study, clean seeds were sown on peat mix in square pots, with stratification at 4 °C for 2 days before moving to growth chamber (22 °C, 120−150 μmol m^−2^ s^−1^ light). Arabidopsis mutants *dcl2-1/3-1/4-2* (CS16391), *hda6-7* (CS66154), *drm1-2/2-2* (CS16383) and *drm2-2* (CS16386) were obtained from ABRC Stock Center. The *met1-3* line was described in Saze et al.^62^. Primers used for screening mutant plants are listed in Supplementary Table 5. The Col-0 used in the study has been sequence-confirmed relative to the public reference genotype. For the *drm1/2* background *msh1* memory screening experiment, a similar approach as described in Fig. 2 was used to generate the *drm1/2* MSH1-RNAi transgenic line used for *msh1* memory screening.

### 5-azacytidine treatment

Col-0 wild-type and *msh1* memory line seeds were surface-sterilized in 10% (v/v) sodium hypochlorite, rinsed thoroughly with sterile water, and sown in 8-oz clear cups (Fabri-Kal, USA) containing 30 mL 0.5 M Murashige and Skoog medium (Sigma, USA) supplemented with 1% (w/v) agar and 0 (control) or 100 μM 5-azacytidine (Sigma, USA). The 100 µM concentration was derived from a concentration gradient experiment of four concentrations (0, 30, 50, 100 µM) where 100 µM showed visible impact on plant growth for both wild-type Col-0 and *msh1* memory line plants. Seeds were germinated and grown at 24 °C, 18-h day length, and 120−150 μmol m^−2^ s^−1^ light intensity for 14 days. For longer observation, the 14-d-treated plants were transferred to square pots with soil and grown under standard conditions in the growth chamber. The experiment was repeated three times, with at least 18 replicates per treatment each experiment. This protocol was adapted from Griffin et al.^63^.

For the purpose of RNA-seq and methylome sequencing, Murashige and Skoog medium (Sigma, USA) supplemented with 1% (w/v) agar was prepared. 5-azacytidine (Sigma, USA) dissolved in dimethyl-sulfoxide (DMSO) was added to cooling agar at final concentration of 100 µM 5-azacytidine. The *msh1* memory line (third generation) and Col-0 wild-type seeds were surface-sterilized with ethanol and 30 seeds per treatment were plated (MS medium containing DMSO with no chemical agent served as 5AZA mock-treated control). Following a 2-d stratification period at 4 °C, seeds were transferred to room temperature, allowed to grow for 8 days under constant light, then harvested, pooled, frozen in liquid nitrogen, and used for RNAseq (total 26 samples) and whole-genome bisulfite sequencing (total 12 samples).

### Circadian clock gene expression assays

To assess the expression pattern of core circadian clock genes under clock-driven free running conditions, plants were entrained at LD condition (12-h light/ 12-h dark) for 4 weeks, then moved to LL (24-h constant light) for 48 h before sample collection was initiated.

For expression of core circadian clock genes under cycling light conditions, plants were entrained at LD (12-h light/12-h dark) for 4 weeks before samples were collected. The entire above-ground plant was collected and placed into liquid nitrogen. Samples were taken every 4 h (ZT6, ZT10, ZT14, ZT18, ZT22, ZT26, ZT30, ZT34, ZT38, ZT42, ZT46, ZT50) in both LD and LL conditions. For each genotype at each time point, at least three plants were collected and used in qPCR experiments as biological replicates. This procedure was adapted from Kay et al.^64^.

### Gene expression analysis by qPCR

For the qPCR experiments, total RNA from each sample was extracted by NucleoSpin RNA Plant kit (Macherey-Nagel, Germany) following the manufacturer’s protocol and genomic DNA removal. First-strand cDNA was synthesized from 400 ng total RNA with oligo primers using iScript Reverse Transcription Supermix for RT-PCR (Bio-Rad, USA). The qPCR was performed on the CFX real-time system (Bio-Rad, USA) with 95 °C for 3 min, 40 cycles of 95 °C for 30 s and 60 °C for 1 min with three biological replicates. RNA abundance of target genes was calculated from the average of four technical replicates using ΔΔCq method, where Cq is the cycle number at which amplification signal reaches saturation in each PCR run. The Cq values of AT4G05320 and AT5G15710 were used as normalization controls in the calculation. This procedure was adapted from MIQE^65^ as standard protocol.

Real-time PCR primers used in this study are listed in Supplementary Table 5. The PCR amplification efficiency was calculated based on a calibration standard curve specific for each primer set, and only primers having amplification efficiency greater than 0.97 were used in the study.

### Bisulfite DNA methylome sequencing experiments

For *msh1* memory transgenerational bisulfite sequencing experiments, *MSH1* RNAi T2 population of 233 progeny plants was developed from a single T1 plant, and 35 were confirmed transgene-null. Among transgene-null individuals, 7 (20%) displayed the *msh1* memory phenotype visually (*msh1* memory Gen1), and 28 showed unaltered (nonmemory) phenotype. Five individual plants of wild-type *Arabidopsis thaliana* ecotype Col-0, five isogenic *msh1* memory plants (Gen1), and five full-sib nonmemory plants were used for sequencing. In subsequent generations, five individual plants of wild-type *Arabidopsis thaliana* ecotype Col-0 and five isogenic *msh1* memory line plants for four generations were sampled. In total, 65 plants were used for sequence analysis.

All wild-type control plants were selected from negative RNAi transformation events and were grown in parallel with *msh1* memory counterparts. Whole plants at early bolting were flash frozen in liquid nitrogen. Samples were ground in liquid nitrogen. A portion of the tissue sample was processed by DNeasy Plant Kit (Qiagen, Germany) for genomic DNA (RNA removed) and subsequent bisulfite sequencing, with the remainder used for RNA extraction by NucleoSpin RNA Plant Kit (Macherey-Nagel, Germany) following the manufacturer’s protocol, including genomic DNA removal, for RNA-seq analysis. Tissues from two Gen1 *msh1* memory and four corresponding wild-type plants were also used for sRNA extraction with the NucleoSpin miRNA Plant Kit (Macherey-Nagel, Germany).

All BSseq experiments were conducted on the Hiseq 4000 or HiSeq X-ten analyzer (Illumina, USA) at BGI-Tech (Shenzhen, China), according to the manufacturer’s instructions. Genomic DNA was sonicated to 100–300 bp fragments and purified with MiniElute PCR Purification Kit (Qiagen, Germany), and incubated at 20 °C after adding End Repair Mix. DNA was purified, a single “A” nucleotide added to the 3′ ends of blunt fragments, repurified and Methylated Adapter added to 5′ and 3′ ends of each fragment. Fragments of 300–400bp were purified with QIAquick Gel Extraction Kit (Qiagen, Germany) and subjected to bisulfite treatment with Methylation-Gold Kit (ZYMO), followed by PCR and gel purification (350–400bp fragments were selected). Qualified libraries were paired-end sequenced on the Hiseq 4000 or HiSeq X-ten system (150 bp read length and at least 4 Gb data per sample). For the six-generation experiment, the 65-sample libraries were loaded into four different HiSeq X-ten flow cells, with a majority (38/65) sharing the same cell. All processing was carried out under a controlled and stringent protocol. Based on the output reads number from each sample (Supplementary Data 1), variation among samples was very low, indicating that batch effect was negligible.

### RNA sequencing and analysis

RNA libraries were constructed as described in the TruSeq RNA Sample Preparation v2 Guide. These libraries were sequenced with the 150-bp reads option, in Hi-Seq 4000 analyzer (Illumina, USA) at BGI-Tech (Shenzhen, China). To enhance resolution of RNA transcription changes in *msh1* memory lines, we generated high depth datasets (at least 80 M read for each sequenced sample). Alignments were performed using STAR (version 2.7.0a) with –twopassMode = Basic and –outFilterMultimapNmax = 1 parameters, retaining only uniquely mapped reads. The read count data were generated from the BAM files by using QoRTs software package (version v1.3.0) with –minMAPQ = 25 option. DESeq2 (version 1.20.0) was used for gene count normalization and to identify DEGs (FDR < 0.05, |*log2FC*| > 0.5).

### sRNA-Seq and analysis

sRNA-seq libraries were constructed from total RNA samples with the Illumina TruSeq Small RNA Sample Preparation Kit and sequenced on a BGISEQ-500 with a single-end 50-bp run length. Small RNA-seq data were aligned to the *Arabidopsis thaliana* genome assembly (version TAIR10) using ShortStack (version 3.8.3) with default parameters except that the “align-only” switch was activated. Each library was individually aligned, followed by merging the resultant bam files using SAMtools merge^66^.

### Methylation analysis

Raw sequencing reads were quality-controlled with FastQC (version 0.11.5), trimmed with TrimGalore! (version 0.4.1) and Cutadapt (version 1.15), then aligned to the TAIR10 reference genome using Bismark (version 0.19.0) with bowtie2 (version 2.3.3.1). 1. The deduplicate_bismark function in Bismark with default parameters was used to remove duplicated reads, and reads with coverage greater than 500 were removed to control PCR bias. Whole-genome bisulfite conversion rate was computed based on chloroplast genome read counts for every sample, with conversion rate >99% for all samples (details in Supplementary Data 1). Four samples from the *msh1* memory transgenerational experiment were excluded from downstream analysis due to poor uniformity issues (61 samples remaining).

Naturally occurring DMPs can be identified in samples from the control and treatment populations. Machine-learning algorithms implemented in Methyl-IT were applied to discriminate treatment-induced DMPs from those naturally generated. After Methylated Cs (COV files) were acquired from Bismark methylation extractor with default parameters, four published DMP identification procedures were tested for classification performance of DMPs obtained by the different methodologies, including Fisher’s exact test (used by methylKit^67^), Wald Test (used by DSS^68^), Root-mean-square test (used by methylpy^69^) and signal detection-machine-learning approach (used by Methyl-IT), with similar methylation level difference (>0.25) and *p* value (<0.05) cutoff. The discrimination power or accuracy of DMP calling for each method was assayed by performance of classifier models built on DMPs identified by each method. For each DMP set from the four methods, we divided DMPs into training set (accounting for 60% of total DMPs) and testing set (accounting for 40% of total DMPs). The machine-learning algorithm was applied to the training set, followed by evaluation of classification performance on the testing set. Methyl-IT was used for further analysis based on its better overall performance.

The basic theoretical approach to DMP identification applied was based on previous published results^70^ and carried out by the R package Methyl-IT (version 0.3.1)^18,71^. Briefly, methylation count (COV) files were read into R and Hellinger Divergence (HD, a variable used to measure methylation level divergence, was calculated by using the pool of wild-type methylation counts as reference).

Potential DMPs (pDMPs) were selected from cytosines with methylation level difference higher than 20% (in the reference vs. treatment comparison) and further estimated based on critical values of HD_*α*_ = 0.05 estimated for each individual from the best fitted probability distribution model, in this case, a two-parameter gamma distribution model. Final DMPs were estimated from the set of pDMPs by estimating the optimal cutoff threshold for HD based on Youden index^72,73^. A full elaboration of the optimal cutoff estimation process is available at https://genomaths.github.io/ Cutpoint_estimation_with_Methyl-IT.html. To further confirm the discrimination power or accuracy of DMP calling for each generation, we divided DMPs into two groups: a training set, accounting for 60% of total DMPs, and a testing set, accounting for 40% of total DMPs. A machine-learning algorithm was applied on the training set, followed by performance of classification on the testing set, with performance of classification verified by cross check with sample ID. In this study, DMPs from all memory vs. nonmemory and WT vs. memory comparisons achieved false discovery rates (FDR) < 0.05 and accuracy > 90% with 999 bootstrap (Monte Carlo) sampling, with the exception of gen6 WT vs. memory at slightly lower accuracy (Supplementary Table 1). Generalized linear regression analysis (generalized linear model, GLM) was applied to test the difference between group DMP counts (WT vs. memory) for selected genomic features. The fitting algorithm approaches provided by glm and glm.nb functions from the R packages stat and MASS were used for Poisson (PR), Quasi-Poisson (QPR) and Negative Binomial (NBR) models with logarithmic link. The “*countTest*” function in Methyl-IT was used to implement the selected model. The following parameters are needed for testing: the minimum DMP count per bp, *p* value adjustment, cutoff for the DMP number difference, cutoff for *p* value and Minimum Mean/Variance rate. For potential DMG identification for transgenerational *msh1* memory, we defined a potential DMG as a gene (ATG to stop codon) with at least 2.5 DMP per 1 kb in each sample, and displaying significant difference between group comparisons in Wald test, given log_2_fold change for group DMP number mean difference > 1, *p* value < 0.05 with Benjamini & Hochberg procedure for *p* value adjustment. Parameter setting was adopted from Yang et al.^6^ and carefully estimated based on our own dataset. In contrast to GbM defined by Takuno and Gaut^74^ and Bewick et al.^38^, where GbM genes are those methylated in CG context but not in non‐CG contexts, we included all genes to the analysis without pre-selection. A detailed description of how to define and compute DMPs and potential DMGs is included in the Methyl-IT vignettes and package manual, available at https://github.com/genomaths/MethylIT and at https://genomaths.github.io/methylit/. Arabidopsis genome annotation file Arabidopsis_thaliana. TAIR10.38. gff3 from ensemble genomes database was used to annotate genome features.

### Network enrichment analysis test

R package neat was used to implement the network enrichment analysis test (NEAT). Network enrichment analysis is an extension of traditional gene enrichment analysis (GEA) tests. A major limitation of GEA tests is that they ignore associations and dependence between genes. The purpose of network enrichment analysis is to integrate GEA tests with information on known relations between genes, represented by means of a gene network. The analysis incorporates genetic networks, with their information on gene dependence, into gene enrichment tests^75^. Throughout the study, the following parameter set was used for the NEAT function: blist = Biological process network from GO database, nettype = directed, only networks having nab > 1 were included in the final output.

### Principal component plus linear discriminant analyses

Principal component plus linear discriminant analyses (PCA-LDA) and hierarchical cluster analysis for 5-azacytidine RNAseq and methylome data were applied by using *prcomp* (implemented in Methyl-IT function *pcaLDA*) and *hclust* functions, respectively, from the R package *stats*^17^. A full description of these analyses (with Methyl-IT) is documented at https://genomaths.github.io/.

### Reporting summary

Further information on research design is available in the Nature Research Reporting Summary linked to this article.

## Supplementary information

Supplementary Information

Peer Review

Reporting Summary

Description of Additional Supplementary Files

Supplementary Data 1-13

## Data Availability

Data supporting the findings of this work are available within the paper and its Supplementary Information files. A reporting summary for this Article is available as a Supplementary Information file. The datasets generated and analyzed during the current study are available from the corresponding author upon request. All next-generation sequencing data generated by this study were deposited to Gene Expression Omnibus database: Arabidopsis methylome for *msh1* memory and nonmemory sibling plants with isogenic Col-0 wild-type control (GSE118874), Arabidopsis *msh1* memory 4-week-old plant RNAseq (GSE106536), Arabidopsis 10-day-old seedling 5-azacytidine treatment RNAseq (GSE109164), Arabidopsis 10-day-old seedling 5-azacytidine treatment methylome (GSE114665), Arabidopsis methylome for the generation 2−6 of *msh1* memory line and isogenic Col-0 wild-type control (GSE129303), Arabidopsis RNAseq for the generation 1 and 5 of *msh1* memory line and isogenic Col-0 wild-type control (GSE129343), and small RNA sequencing of *msh1* memory line and isogenic Col-0 wild-type control in Arabidopsis (GSE134028). The source data underlying Figs. 1b, d, 3a, b, 4c, d, 5, 6, 8e, f as well as Supplementary Figs. 1c, 2, 9−12, 14, and 20 are provided as a Source Data file.

## Source data

Source Data

## Code availability

Custom codes used for methylation analysis with Methyl-IT, DMP classification based on signal detection, principal components and linear discriminant analyses with Methyl-IT are available at https://genomaths.github.io/.
