# Supplementary material for: Segregation of an MSH1 RNAi transgene produces heritable non-genetic memory in association with methylome reprogramming
Source: Nat Commun. 2020 May 5;11:2214. doi: 10.1038/s41467-020-16036-8 (PMC7200659; doi:10.1038/s41467-020-16036-8)
Supplement: Supplementary file 1 — Supplementary Information [file 41467_2020_16036_MOESM1_ESM.pdf]

Segregation of an *MSH1* RNAi transgene produces heritable non-genetic memory in association with methylome reprogramming

Yang *et al.*

Supplementary Table 1. The classification performance of DMPs.

	Classification model	Total Classification events	Accuracy	Sensitivity	FDR
NM vs MM	PCA-QDA	371958	0.9624	0.9998	0.042312
Gen1 WT vs MM	PCA-QDA	403342	0.9895	0.998	0.009586
Gen2 WT vs MM	PCA-QDA	354925	0.9042	0.9066	0.028395
Gen3 WT vs MM	PCA-QDA	375064	0.905	0.9167	0.037389
Gen4 WT vs MM	PCA-QDA	435864	0.9627	0.9956	0.038864
Gen5 WT vs MM	PCA-QDA	444159	0.9206	1	0.094945
Gen6 WT vs MM	PCA-QDA	342283	0.8538	0.831	0.010395

Performance of classifier models built on DMPs using principal component plus quadratic discriminant analysis, PCA-QDA. To further confirm the discrimination power or accuracy of DMP calling for each comparison, we divided DMPs into two groups: training set (accounting for 60% of total DMPs) and testing set (accounting for 40% of total DMPs). The machine learning algorithm was applied to the training set, followed by evaluation of the classification performance on the testing set. In this study, DMPs from all memory vs non-memory and WT vs memory comparisons achieved False Discovery Rate (FDR) <0.05 and accuracy > 90% with 999 bootstrap samplings. The code used to generate this table is available at https://genomaths.github.io/

Supplementary Table 2. Segregation analysis of *msh1* $^{+/-}$ / *hda6-7* $^{-/-}$ (F_3) plants.

	On soil	
Progeny genotype	Expected # of progeny	Observed # of progeny
msh1 $^{+/+}$ / *hda6-7* $^{-/-}$	31.5	94
msh1 $^{+/-}$ / *hda6-7* $^{-/-}$	63	32
msh1 $^{-/-}$ / *hda6-7* $^{-/-}$	31.5	0
Total	126	126

Supplementary Table 3. Segregation analysis of *msh1* $^{-/-}$ / *met1* $^{+/-}$ (F_3) plants.

	On 0.5 M MS medium	
Progeny genotype	Expected # of progeny	Observed # of progeny
msh1 $^{-/-}$ / *met1* $^{+/+}$	88.75	129
msh1 $^{-/-}$ / *met1* $^{+/-}$	177.5	193
msh1 $^{-/-}$ / *met1* $^{-/-}$	88.75	33
Total	355	355

Supplementary Table 4. Segregation analysis of control *met1* +/- plants.

	On 0.5 M MS medium	
Progeny genotype	Expected # of progeny	Observed # of progeny
met1 $^{+/+}$	77.5	96
met1 $^{+/-}$	155	152
met1 $^{-/-}$	77.5	62
Total	310	310

Supplementary Table 5. Primers used in this study.

Target	Primer name	Sequence 5'-3'
	**Arabidopsis real-time PCR experiment**	
AT5G15710	(Reference gene)	TTTCGGCTGAGAGGTTCGAGT
AT5G15710	(Reference gene)	GATTCCAAGACGTAAAGCAGATCAA
UBQ10 (AT4G05320)	(Reference gene)	GGCCTTGTATAATCCCTGATGAATAAG
UBQ10 (AT4G05320)	(Reference gene)	AAAGAGATAACAGGAACGGAAACATAGT
MSH1(AT3G24320)	Qrt-msh1-F4	TAACCTAGCCGATGGAAAGC
MSH1(AT3G24320)	Qrt-msh1-R4	AGAGATGATCAACTACAAACACAGC
CCA1 (At2g46830)	Qrt-CCA1-PNAS-F	CAGCTCCAATATAACCGATCCAT
CCA1 (At2g46830)	Qrt-CCA1-PNAS-R	CAATTCGACCCTCGTCAGACA
GI (At1g22770)	Qrt- GI -PNAS-F	ACTAGCAGTGGTCGACGGTTTATC
GI (At1g22770)	Qrt- GI -PNAS-R	GCTGGTAGACGACACTTCAATAGATT
TOC1 (At5g61380)	Qrt- TOC1 -PNAS-F	AATAGTAATCCAGCGCAATTTTCTTC
TOC1 (At5g61380)	Qrt- TOC1 -PNAS-R	CTTCAATCTACTTTTCTTCGGTGCT
LHY (At1g01060)	LHY-QF	GACTCAAACACTGCCCAGAAGA
LHY (At1g01060)	LHY-QR	CGTCACTCCCTGAAGGTGTATTT
ERF2(At5g47220)	Qrt -At-ERF2-F	CGGACTCCTCAAAGATGCCT
ERF2(At5g47220)	Qrt -At-ERF2-R	CTCTGCCTCACTCCTCTGTAATG
ABF1(AT1G49720)	Qrt -At-ABF1 -F	TGATCAAGAATCGGGAATCCG
ABF1(AT1G49720)	Qrt -At-ABF1 -R	TCAATCTCAGCTTCCAGTTCC
ERF1(AT3G23240)	Qrt -At-ERF1 –F	TGAGACGGAGAATGACCAATAAG
ERF1(AT3G23240)	Qrt -At-ERF1 –R	GGTACTGTTCTCCCAAATCCTC
APRR3(AT5G60100)	Qrt-APRR3-F	CGAGTTCAAGCAGTGACAATC
APRR3(AT5G60100)	Qrt-APRR3-R	CTTCCTGCTATGGTACCTAACC
CRY2(AT1G04400)	Qrt-CRY2-F	TCGTTTCGCAGTCTTGCTC
CRY2(AT1G04400)	Qrt-CRY2-R	ACACATCTACATGTTAGGCTTTATAAC
PHYA(AT1G09570)	Qrt-PHYA-F	TGGCGGTTGATTCTGATGG
PHYA(AT1G09570)	Qrt-PHYA-R	GAAATGCTTCCCGATTGCTTC
ELF3(AT2G25930)	Qrt-ELF3-F	GTGACGAGAGTGATAAAGGTGG
ELF3(AT2G25930)	Qrt-ELF3-R	GACTGGAAAATTCTGGCAGC
FKF1(AT1G68050)	Qrt-FKF1-F	ACATGAACTCTGCTTGGCTAG
FKF1(AT1G68050)	Qrt-FKF1-R	TGACAGATACAAAACCACAAACC
CDF1(AT5G62430)	Qrt-CDF1-F	GTCCTACTTCTACTCTTGGTAAGC
CDF1(AT5G62430)	Qrt-CDF1-R	CATATCGAACTCTTTGCAGCTTC
ERS1(AT2G40940)	Qrt-ERS1-F	TGTGGATAGAAAGTGAAGGCC
ERS1(AT2G40940)	Qrt-ERS1-R	CGGTCTGGTTTGTGATTTAGC
EBF2(AT5G25350)	Qrt-EBF2-F	GGAGTCTCTTAACCTTGACGG
EBF2(AT5G25350)	Qrt-EBF2-R	CCTTGATTCCGTGATCTGAGAC
EIL1(AT2G27050)	Qrt-EIL1-F	ATGTCCAAAGCAACCAAACG
EIL1(AT2G27050)	Qrt-EIL1-R	ACCGATTGTTAACCCCGTTG
CTR1(AT5G03730)	Qrt-CTR1-F	TGGAAGGAGTGCATTGATGG

CTR1(AT5G03730)	Qrt-CTR1-R	CATCCTTTGGCAATTCGACAG
SRK2D(AT3G50500)	Qrt-SRK2D-F	ATGGATGAGAACCGAATGGG
SRK2D(AT3G50500)	Qrt-SRK2D-R	TCTGCATAATCGTGTCAAGGC
ABF1(AT1G49720)	Qrt-ABF1-F	TTCCTTACGTGTTTGGTCGG
ABF1(AT1G49720)	Qrt-ABF1-R	TTCCAGTTCCAAGGTATAAGCC
BOA(AT5G59570)	Qrt-BOA-F	CTGTTGGTGGTGGTGACGTG
BOA(AT5G59570)	Qrt-BOA-R	CCATAAGCCAAGAACCAGTATCTC
PHYC(AT5G35840)	Qrt-PHYC-F	AAGAAGGCTATGTGGAACTGGA
PHYC(AT5G35840)	Qrt-PHYC-R	TGCTCAGCTCCATCACTTGTT
MET1(AT5G49160)	Qrt-MET1-F	TTGTCAAGCCTGTTGAGCCA
MET1(AT5G49160)	Qrt-MET1-R	TTGCATCAGATACACCCGCC
NRPD1A(AT1G63020)	Qrt-NRPD1A-F	CAAAGGAATCCGGGTCGCTA
NRPD1A(AT1G63020)	Qrt-NRPD1A-R	TGTTGCTCCCAAGACACACT
SAP13(AT3G57480)	Qrt-SAP13-F	TGCCCTGACCTGTCAGTTAAT
SAP13(AT3G57480)	Qrt-SAP13-R	CCTCGGGAGTTGACCACATC
SRR1(AT5G59560)	Qrt-SRR1-F	CCACACTGTGAGGCCAATCT
SRR1(AT5G59560)	Qrt-SRR1-R	GCTGTTCCCAAACAATGCGA
RH40(AT3G06480)	Qrt-SRR1-F	AGTCACAACAACTGGCGAGA
RH40(AT3G06480)	Qrt-SRR1-R	CGGTGTTGGCGATGGAAATC
U4/U6(AT4G22410)	Qrt-U4/U6-F	AGTGCTTCCAGGGTGAGTTG
U4/U6(AT4G22410)	Qrt-U4/U6-R	CGGTGGCAAATCTAGACCGA
	**Arabidopsis bisulfite PCR experiment**	
DDM1(At5g66750)	BS-control_F: (converted DNA):	TGTTTGGTGATTTATTTATTTTTGTTTTTAATG
DDM1(At5g66750)	BS-control_R :(unbiased):	CTCTCACTTTCTATCCCATTCTA
GI (At1g22770)	BS-GI-F7	TGAGTTTAAGAATTTTATGTTAATATTTTTTGG
GI (At1g22770)	BS-GI-R7	CTATCAACRTAACAATCTCATATC
GI (At1g22770)	BS-GI-F2	ATAATAYGGTGTTTTTTATATTTAAGTG
GI (At1g22770)	BS-GI-R2	TCRAAAAACTAAATAAACATAATTATAAAAAAC
XTH16(AT3G23730)	BS-AT3G23730-F1	AAATGAYGATTTTGTTTTTAAATGTGTTGATGAG
XTH16(AT3G23730)	BS-AT3G23730-R1	CTAAACATCATCCCTTAAAATTTAAATATCTTACATC
	**Arabidopsis mutant screening**	
	Met1-MUT-R	GGTTCTTATAGGGTTTCGCTC
	Met1-F	GATTGTGTCTCTACTACAGAGGC
	Met1-WT-R	GTTAAGCTCATTCATAGCCTTGC
	hda6-7-geno-F	GATTCTGAGTGAGAGACGGAG
	hda6-7-geno-F	AGCCATACGGATCCGGTGAGG
	DCL2-1-RP	CTTCACAGGAGTTTTTGGCTG
	DCL2-1-LP	TGAATCATCTGGAAGAGGTGG
	DCL3-p1	CTGAATATGGATAATAAGTTTGAGACATATC
	DCL3-p2	GGACTCAATGCAATATAGAGCTTT
	Salk_LBb1.3	ATTTTGCCGATTTCGGAAC
	DCL4-2-RP	TTTGCCAGTCTTACAAGTGGG

DCL4-2-LP	CAGAAGAGCAATCGAAGAGAACTT
Gabi-8474	ATAATAACGCTGCGGACATCTACATTTT
drm2-2-RP	TTGTCGCAAAAAGCAAAAGAG
drm2-2-LP	AGATCGCTTCCAGAGTTAGCC

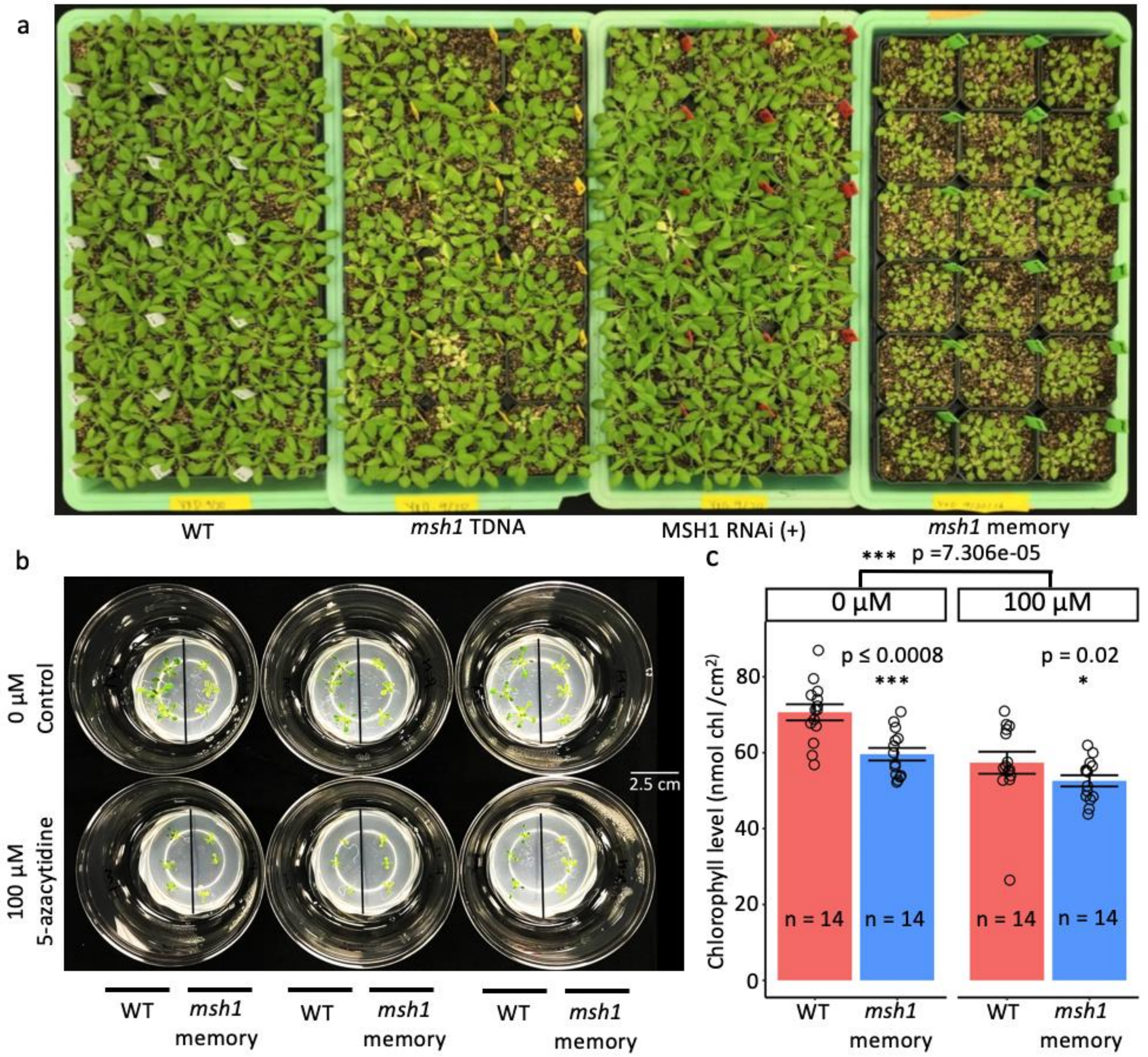

Supplementary Fig. 1. The memory line phenotype. a, Stability of the *msh1* memory line phenotype. **b,** Plants germinated on MS medium containing 0 μM or 100 μM 5-azacytidine; photo at 10 days after germination. **c**, Chlorophyll measurements of wild type (WT) and *msh1* memory plants after 5-azacytidine treatment. Bars represent means ± SD. Plants grown on MS medium with 0 μM or 100 μM 5-azacytidine for 10 days, then moved to normal soil conditions. Chlorophyll level was measured with a SPAD-502 meter two weeks after transplanting. Mann-Whitney tests for the comparison WT versus msh1 memory were accomplished for one and two-sided alternative hypotheses. For the treatment with 0 μM 5-AZA, all the tested differences were found statistically highly significant (p-value $\leq 8.633 \times 10^{-4}$). The significance code "***"

signifies the results of Mann-Whitney test with one-sided alternative hypothesis (the location of WT distribution is shifted to the right of msh1-memory). For the treatment with 100 µM 5-AZA, statistically significant differences were found for the one-sided alternative hypothesis (the location of WT distribution is shifted to the right of msh1-memory) with *p*-value = 0.02405 and for the two-sided test with with *p*-value = 0.04809. Generalized linear mixed model fit by maximum likelihood was used with *msh1* effect as random effect in a model, with factors 5-AZA and Line. The ANOVA type II (Type II Wald Chi-square tests) for the model indicated a highly statistically significant main effects of the both factors. It was not found evidence to support a significant effect of the factor interaction 5-AZA: Line (*p*-value = 0.2293). The code "***" at the top of panel **c** stands for the significance level of the main effect of Line. All the statistical analyses were performed in R software environment for statistical computing and graphics, version 3.6.2. R package "lmerTest" version 3.1.1 was used to implement the generalized linear mixed model model[1] and the ANOVA tests for the models were performed with function "Anova" from the R package "car", version 3.0.7. Source data underlying Supplementary Fig. 1c are provided as a Source Data file.

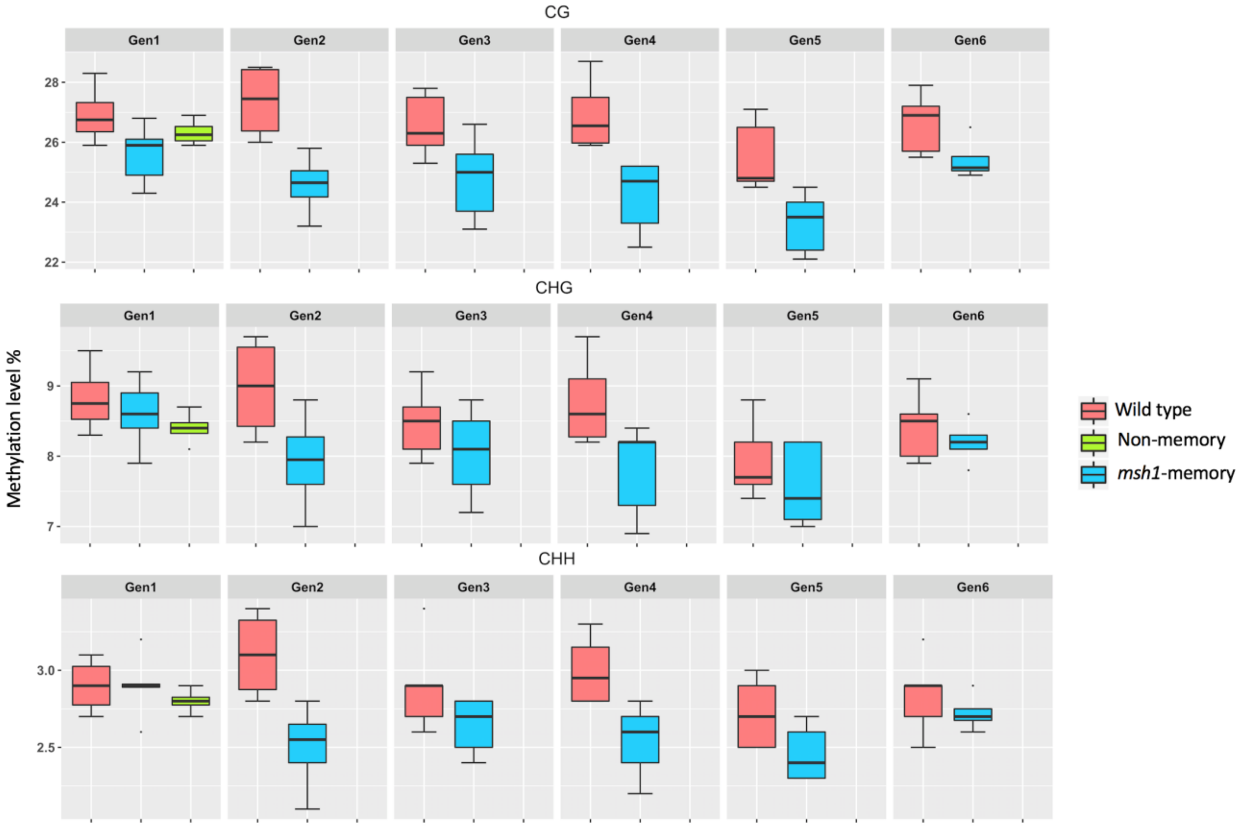

Supplementary Fig. 2. Genome-wide methylation dynamics within the transgenerational *msh1* memory line. Genome-wide weighted methylation levels of wild type control and *msh1* memory line in each sequence context were calculated as the frequency of C base calls at C sites within whole genome divided by the sum of the frequencies of C and T base calls at C sites within whole genome[2]. Data are represented as boxplots where the middle line is the median, the lower and upper hinges correspond to the first and third quartiles, the upper whisker extends from the hinge to the largest value and the lower whisker extends from the hinge to the smallest value, while data beyond the end of the whiskers are outlying points that are plotted individually, sample size n=5(except non-memory, Gen1 WT, Gen2 memory, Gen6 memory, where sample size n=4). Source data are provided as a Source Data file.

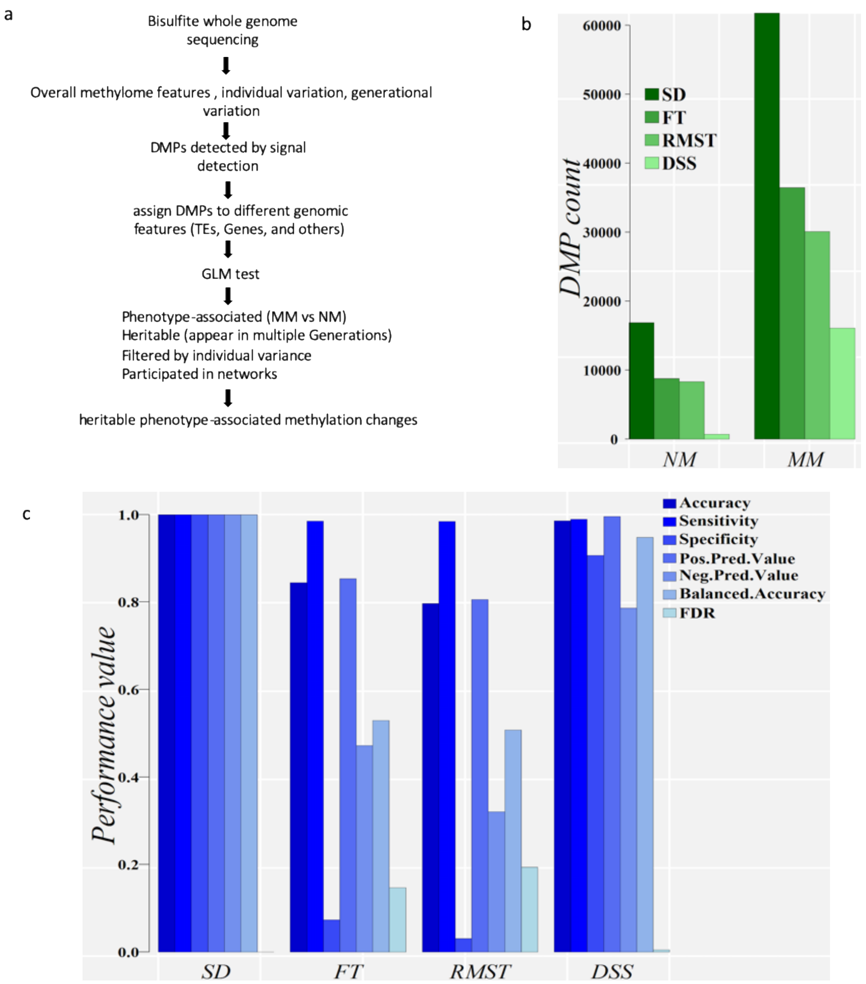

Supplementary Fig. 3. Performance for the control and treatment DMPs obtained by different methodologies. **a**.The methylome analysis pipeline implemented in the study to track heritable phenotype-associated methylation changes. **b**, DMP number identified by four methylation analysis approaches**,** FT: Fisher's exact test, used by methylKit, DSS: an R package that uses generalized linear regression and the Wald Test in the DMP identification, RMST: Root-mean-square test, used by methylpy, SD: Signal detection approach, implemented in MethylIT**.** For each method, the average DMP number for 5 nonmemory (NM) and memory (MM) plants are shown**.** **c**, Seven classifier performance indicators estimated for DMP calling method. The CG, CHG and CHH DMPs from NM and MM comparison were used for **b**. For **b** and **c**, Performance of classifier models built on DMPs (Principal component plus quadratic discriminant analysis, PCA-QDA). To further confirm the discrimination power or accuracy of DMP calling for each comparison, we divided DMPs into two groups: training set (accounting for 60% of total DMPs) and testing set (accounting for 40% of total DMPs). The machine

learning algorithm was applied to the training set, followed by evaluation of the classification performance on the testing set. In this study, DMPs from all memory vs non-memory and WT vs memory comparisons achieved False Discovery Rate (FDR) <0.05 and accuracy > 90% with 999 bootstrap samplings. Results confirm that *p*-values alone are not sufficiently robust for decision-making (DMPs identified with FT, RMST, and DSS).The source data associated with this figure is available on Expression Omnibus database dataset (accession number GSE118874 and GSE129303).The code used to generate this figure is available at https://genomaths.github.io/.

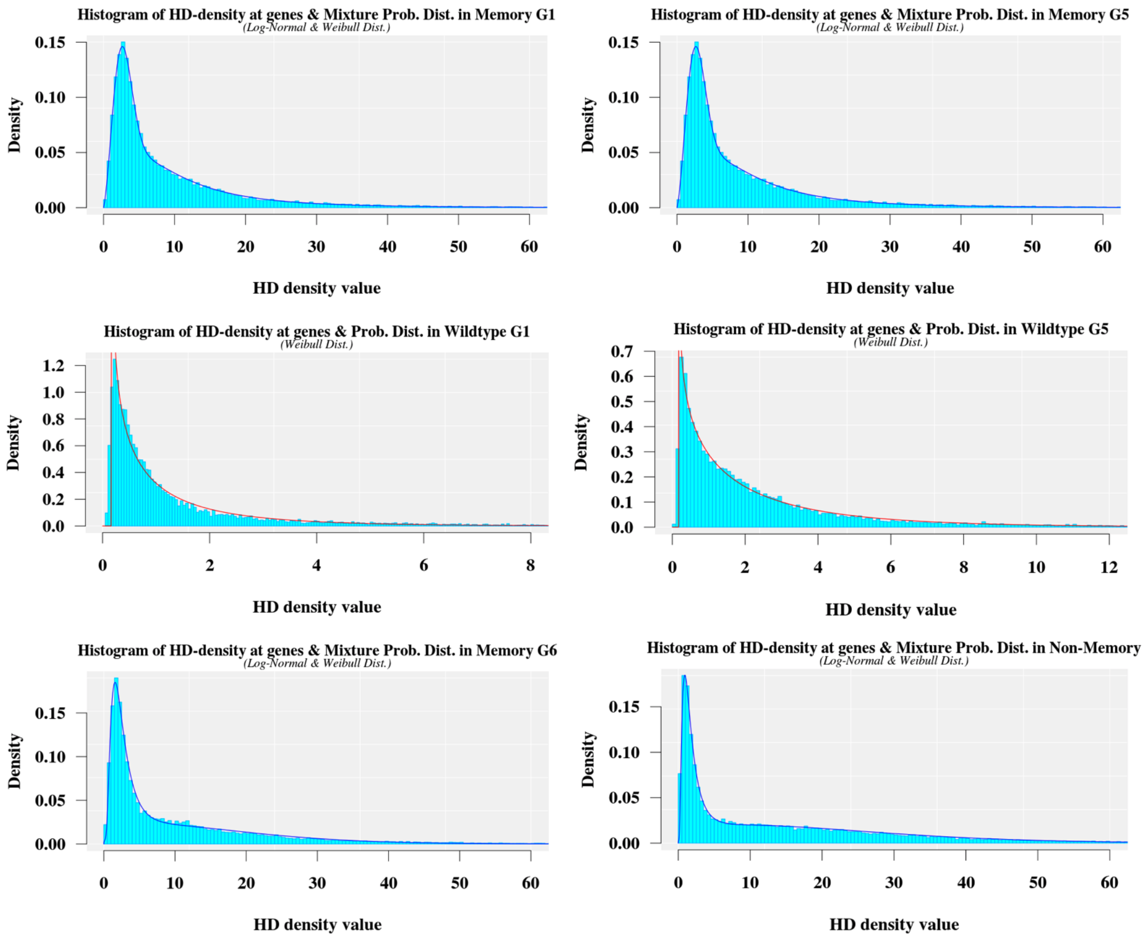

Supplementary Fig. 4. Methylation divergence density probability distribution.

Methylation divergence probability density distribution of memory, non-memory and wild type plants. Methylation divergence (Hellinger divergence) for each sample is computed as described in Sanchez et. al.[3] The distributions from memory and nonmemory can be described by a mixture of Log-Normal and Weibull probability distributions, while the wild type is described by just a Weibull distribution; gen6 has a distribution resembling nonmemory. The source data associated with this figure is available on Expression Omnibus database dataset (accession number GSE118874 and GSE129303). Analysis method is at https://genomaths.github.io/.

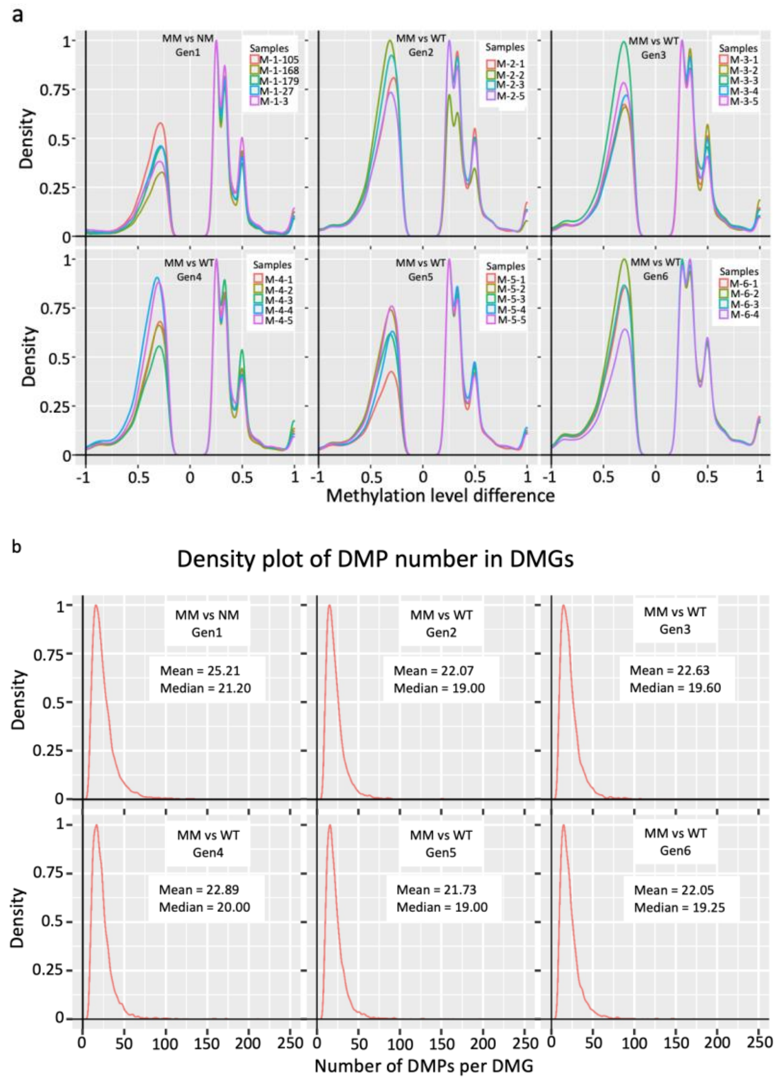

Supplementary Fig. 5. Density plot of DMP methylation level difference and DMP number in DMGs. **a,** Density plot of DMP methylation level difference. Methylation level differences at each cytosine were computed by $(mC/(mC + uC))_{\text{each individual}} - mC/(mC + uC)_{\text{average of all reference plants}}$, with mC denoting methylated cytosine and uC denoting unmethylated cytosine. For gen1, non-memory (NM) plants were used as reference. For gen2 to gen6, wild type plants were used as the reference for each generation. The minimum methylation level difference is 0.2. **b**, Density plot of DMP number in DMGs. The average DMP number in DMGs of memory (MM) plants from each generation (For gen1, non-memory (NM) plants were used as reference. For gen2 to gen6, wild type plants were used as the reference for each generation) were used to generate this density plot. The minimum DMP number for each DMG is 8. For both a and b, scaled density plots using kernel density estimation algorithm were generated by the

geom_density function from ggplot2 (R package). Sample size n=5(except non-memory, Gen2 memory, Gen6 memory, where Sample size n=4). The source data associated with this figure is available on Expression Omnibus database dataset (accession number GSE118874 and GSE129303).

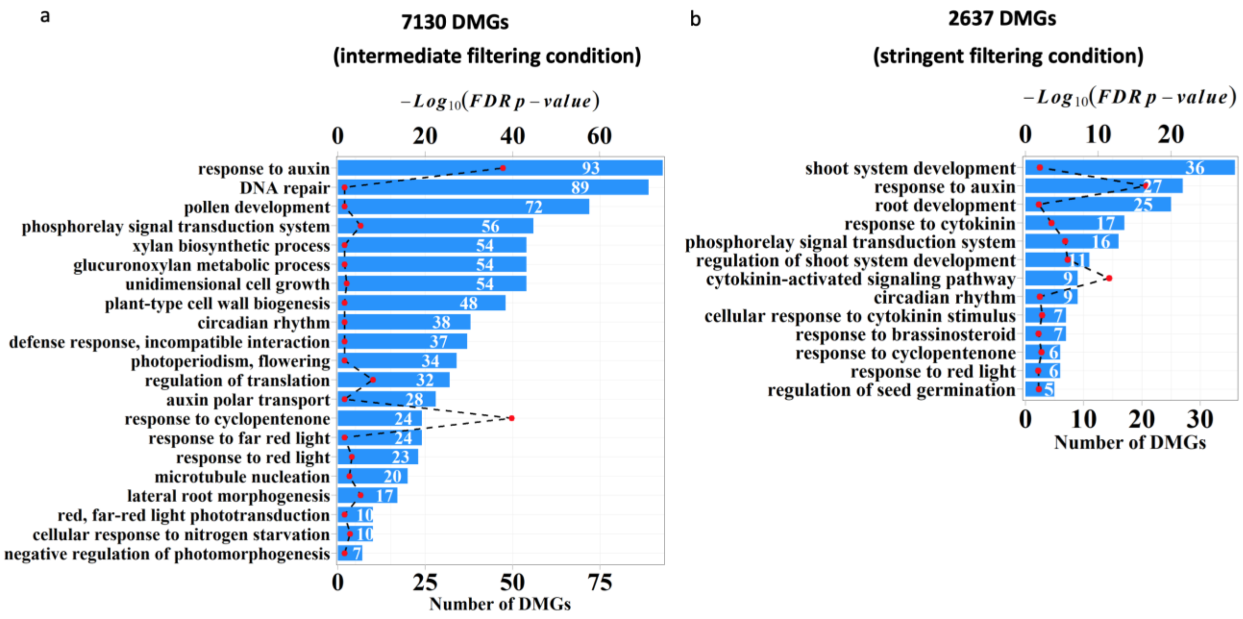

Supplementary Fig. 6. Network-based enrichment analysis. The NBEA analysis of nonmemory (NM) vs memory (MM) DMGs identified by two different filter stringency conditions**. a,** Enriched networks from 7130 DMGs (criteria for intermediate filtering: minimum reads count per cytosine > 4, DMP number per gene > 8, log2FC > 1, *p*-value < 0.05, implemented with *countTest2* function in Methyl-IT). **b**, Enriched networks from 2637 DMGs (intermediate filter plus additional filter of minimum methylated reads count per cytosine > 3, implemented with *estimateDivergence* function in Methyl-IT). The source data associated with this figure is available on Expression Omnibus database dataset (accession number GSE118874 and GSE129303).

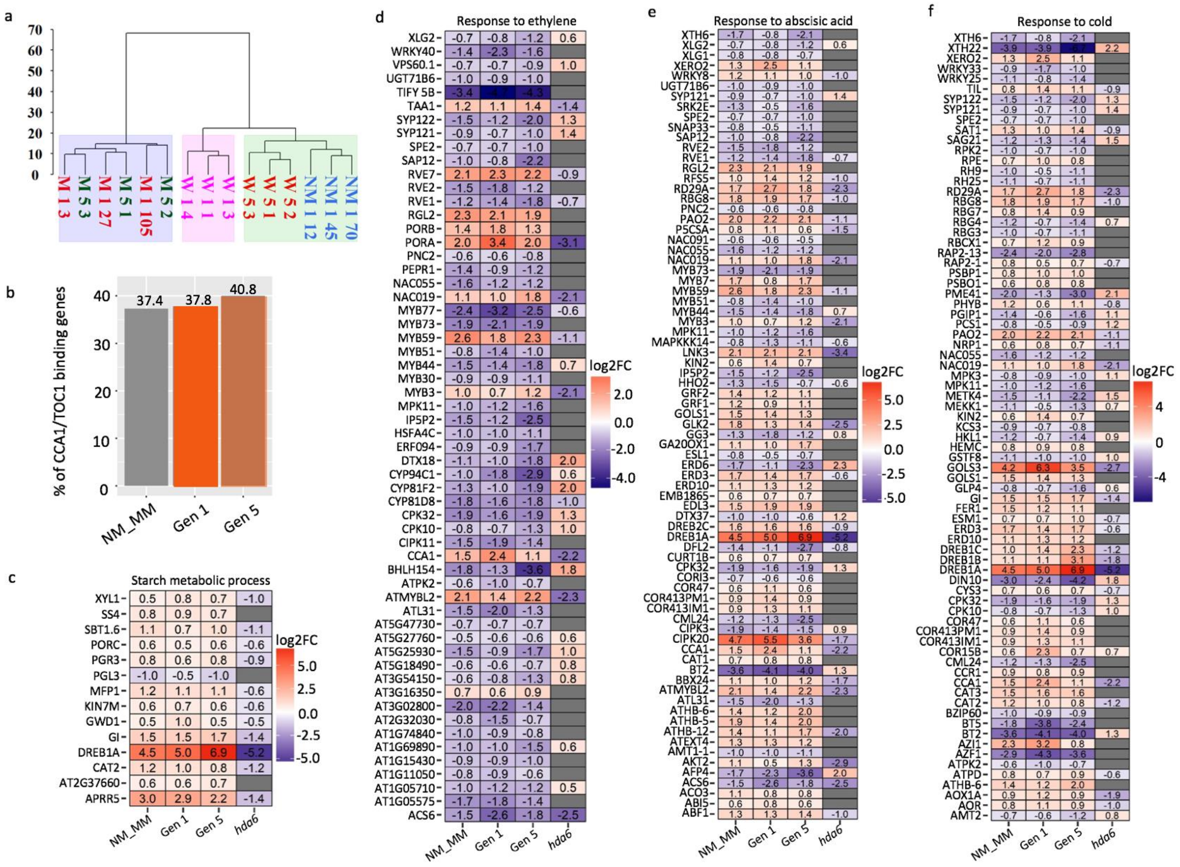

Supplementary Fig. 7. Summary of DEG analysis. **a**, Hierarchical cluster for the RNAseq read counts of 15 samples from gen1, 5 wild type plants (W), 5 non-memory plants (NM) and 5 *msh1* memory (MM) for the set of all annotated and expressed genes. **b**, 37.3%, 37.8% and 40.8% of the 1982 CCA/TOC1-binding genes[4] were identified as DEGs in NM vs MM, gen1 WT vs MM and gen1 WT vs MM comparisons, respectively. Selected circadian clock-regulated downstream networks and individual genes in the *msh1* memory line are present in **c**, Starch metabolic process; **d**, response to ethylene; **e**, response to abscisic acid; and **f**, response to cold. The of read counts for each gene is shown. The source data of this figure, which is the full list of DEGs and their expression level can be found in Supplementary Data 5-7.

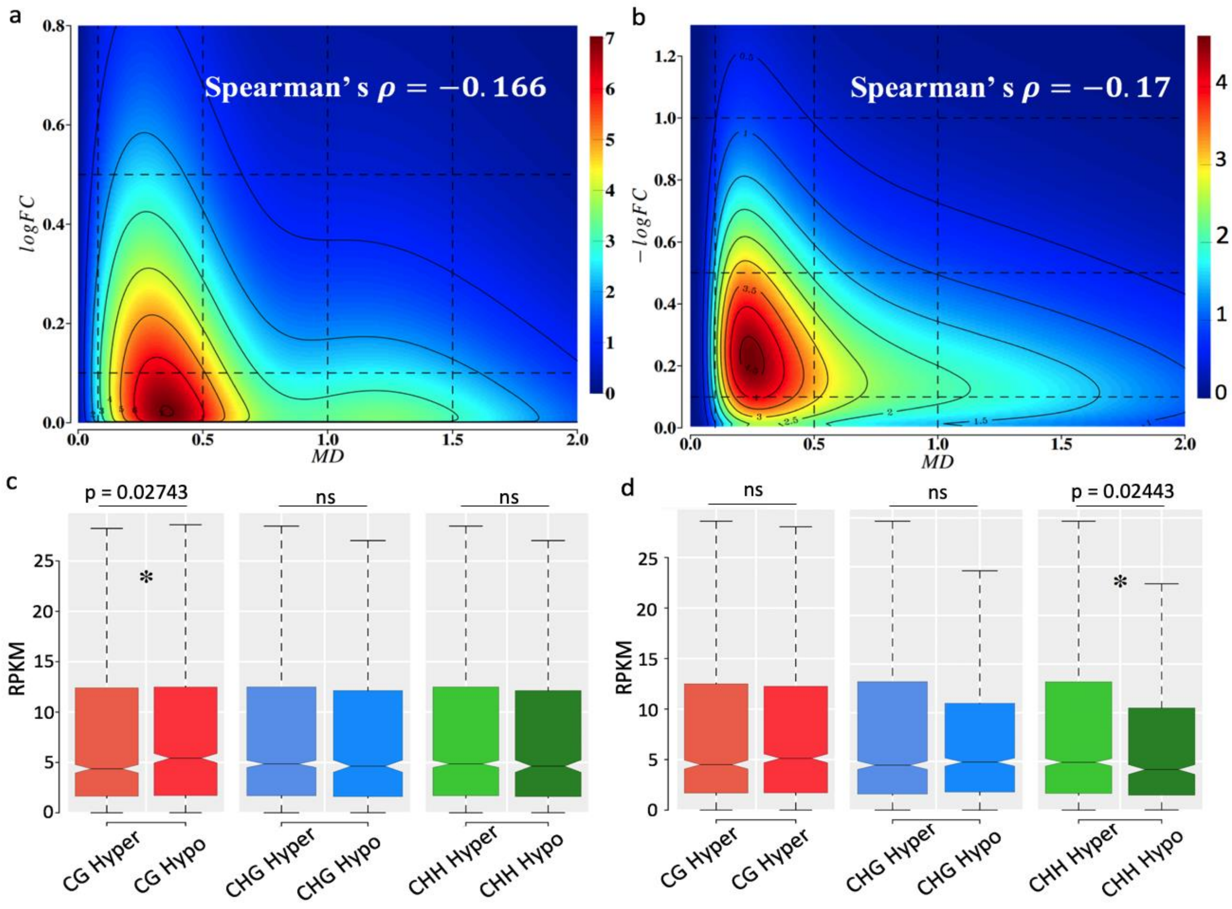

Supplementary Fig. 8. Association of DNA methylation divergence with gene expression. Two-dimensional density plot log_2FC versus methylation divergence (MD) on gene-body regions. **a,** Genes with pairwise values $MD > 0$ and $log_2FC > 0$ (6060 genes). **b,** Genes with pairwise values $MD > 0$ and $log_2FC < 0$ (13962 genes). Results are consistent with negative trends expressed in the Spearman' s $\rho = -0.166$ and $\rho = -0.17$ (both highly significant, with p-value $< 2.2 \times 10^{-16}$). A linear statistical association between log_2FC and MD was confirmed with the application of a linear-by-linear association test (with p-value $< 2.2 \times 10^{-16}$). For data from both panels, **a** and **b**, an association was also found between log_2FC and MD at promoter regions, but weaker than at gene-body, with Spearman' s $\rho = -0.072$ (also significant). The full statistical analysis (with R source code) of the association reported in panels **a** and **b** is available at: https://genomaths.github.io/methylit/articles/GenExp_Methylation_association_hyper_down-regulated.html. **c** and **d**, Notched boxplots of gene expression (RPKM) versus DNA methylation in gen1 *msh1* memory plants. In the Notched boxplots, the middle line is the median, the notch

represent 95% confidence interval of median, the lower and upper hinges correspond to the first and third quartiles, the upper whisker extends from the hinge to the largest value and the lower whisker extends from the hinge to the smallest value.**c**, gene expression vs gene-body DNA methylation. **d**, Gene expression vs DNA methylation at 2kb upstream of TSS (Transcription Start Site). For each gene/2kb-upstream-TSS region, DNA methylation was represented as differences of groups mean methylation levels. Statistically significant differences were calculated by Mann-Whitney U test with one-side alternative hypothesis (*p-value < 0.05). Specifically, the one-sided p-value = 0.02743 (distribution of RPKM values in CG-hypo-methylated DMGs is shifted to the right of the distribution found in CG-hyper-methylated DMGs) in panel **c** and the one-sided p-value = 0.02443 (distribution of RPKM values found in CHH-hyper-methylated DMGs is shifted to the right of the CHH-hypo-methylated DMGs) in panel **d**. ns not significant. RPKM values (Reads Per Kilobase of transcript, per Million mapped reads) were computed by R package edgeR. 821 genes (both DMGs and DEGs) from gen1 *msh1* memory were used. The source data associated with this figure is available on Expression Omnibus database dataset (accession number GSE118874, GSE129303 and GSE129343). For all panel, the methylation and gene expression information from 4 Gen1 WT plants (n=4) and 5 msh1 memory plants (n=5) was used.

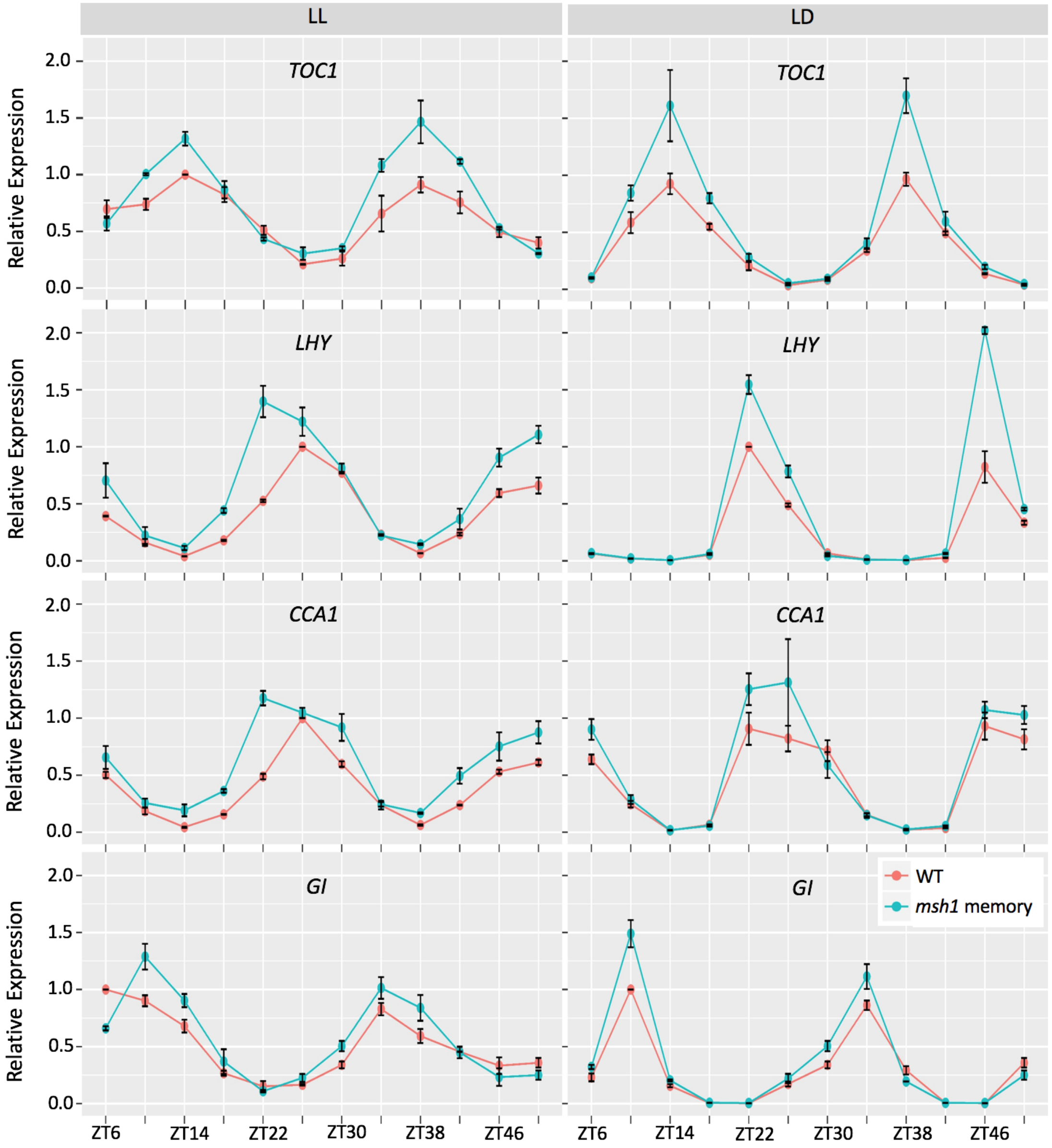

Supplementary Fig. 9. Arabidopsis *msh1* memory line circadian rhythm behavior.

Relative expression levels of indicated genes in wild type (red line) and *msh1* memory (cyan line) grown under LL (24 hours light) after entrainment for 4 weeks under LD (12 hours light,12 hours dark) or remaining under LD. ZT hours used to indicate sampling time (with ZT0 at lights on). Transcript levels were measured by qPCR. Expression levels were normalized to the highest peak of WT control. Error bars represent mean ± SD of three independent biological replicates. Source data are provided as a Source Data file.

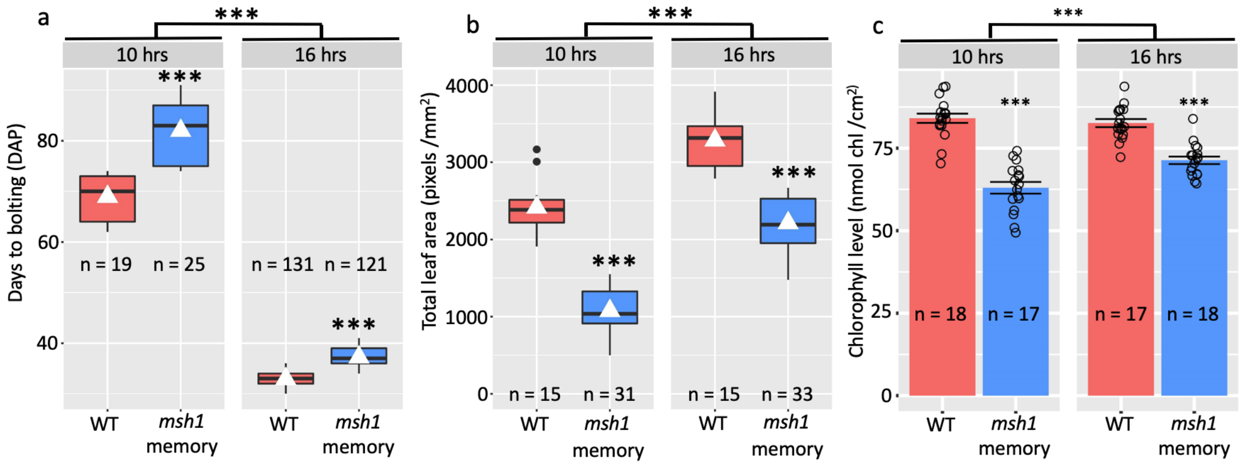

Supplementary Fig. 10. The *msh1* memory line phenotype under different day lengths (10-hour vs 16-hour). Measured parameters in *msh1* memory line included: **a**, Days to bolting. **b**, total leaf area and **c**, chlorophyll level. Bars represent means ± SE. For panel **a** and **b**, data are represented as boxplots where the middle line is the median, white triangle represent the mean, the lower and upper hinges correspond to the first and third quartiles, the upper whisker extends from the hinge to the largest value and the lower whisker extends from the hinge to the smallest value, while data beyond the end of the whiskers are outlying points that are plotted individually. In each panel, for each day-length, Mann-Whitney tests for the comparison WT versus msh1 memory were accomplished for one and two-sided alternative hypotheses. All the tested differences were found statistically highly significant (*p*-value < 0.001). In panel **a**, for each day-length, start code "***" stand for the results of Mann-Whitney test with one-sided alternative hypothesis (the location of msh1-memory distribution is shifted to the right of WT), which found statistically highly significant shifting with *p*-value $\leq 3.167 \times 10^{-8}$. In panel **b** and **c**, for each day-length, the significance code "***" stands for the results of Mann-Whitney test with one-sided alternative hypothesis (the location of WT distribution is shifted to the right of msh1-memory), which found statistically highly significant shifting with *p*-values lesser than 5.44×10^{-8} and 4.754×10^{-7} in **b** and in **c**, respectively. Generalized linear mixed model fit by maximum likelihood was used with *msh1* effect as random effect in a model, with factors Day-Length (DL) and Line (L), for the experiments from panels **a**, **b**, and **c**. The ANOVA type III (Type III Wald Chi-square tests) for the model indicated a highly statistically significant effect of the factor interaction (DL: L) on the three measured response variables (*p*-values: 0.0002803,

2.355×10^{-7}, and 0.0001981 in panels **a**, **b**, and **c**, respectively). That is, although the (main) effect of the factor Line on each one of the response variables was found statistically highly significant (p-values $< 2.2 \times 10^{-16}$), the magnitude of the Line effects on the each one of the response variables depends on the day-length. Significance codes: * (p < 0.05), ** (p < 0.01), *** (p < 0.001). The code "***" at the top of each panel stands for the significance level of the interaction DL: L. All the statistical analyses were performed in R software environment for statistical computing and graphics, version 3.6.2. R package "lmerTest" version 3.1.1 was used to implement the generalized linear mixed model model[1] and the ANOVA tests for the models were performed with function "Anova" from the R package "car", version 3.0.7. Source data are provided as a Source Data file.

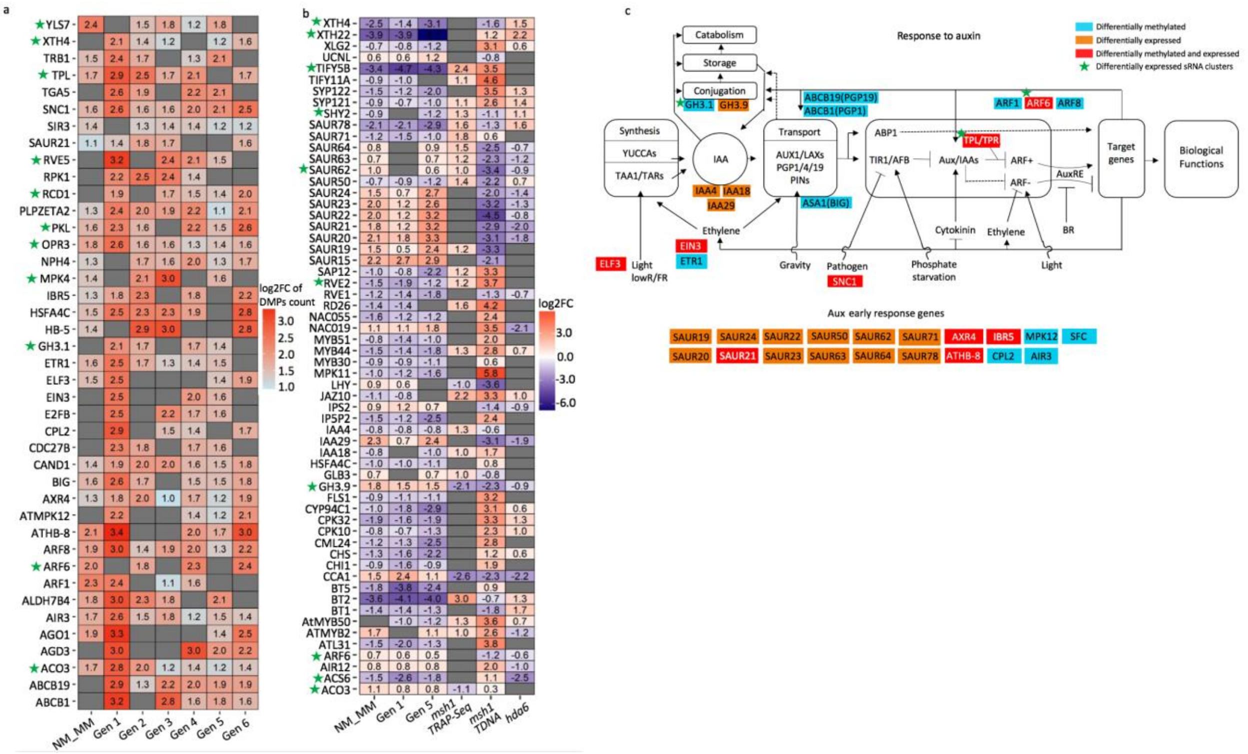

Supplementary Fig. 11. Differential expression and methylation of the auxin response network in *msh1* memory. a, Identified DMGs from the auxin response (GO:0009733) network in *msh1* memory line. Only genes identified as DMGs in at least 4 of 7 comparisons (Gen1 to Gen6 WT vs. MM and Gen1 NM vs MM) are presented. The scale represents the log2 Fold change of DMP number at specific genes in each comparison. **b**, Retained DEGs from the auxin response (GO:0009733) network in *msh1* memory line and *msh1* mutant. Only genes identified as DEGs in at least 4 out of 5 comparisons (Gen1 WT vs. MM, Gen1 NM vs. MM, Gen5 WT vs. MM, WT vs. *msh1* translatome (TRAPseq) and WT vs. *msh1* TDNA) are presented. **c**, DMGs and DEGs presented in panel **a** and **b** are positioned within the network. The auxin response network was adapted from Kieffer *et al.*[5]. Source data are provided as a Source Data file.

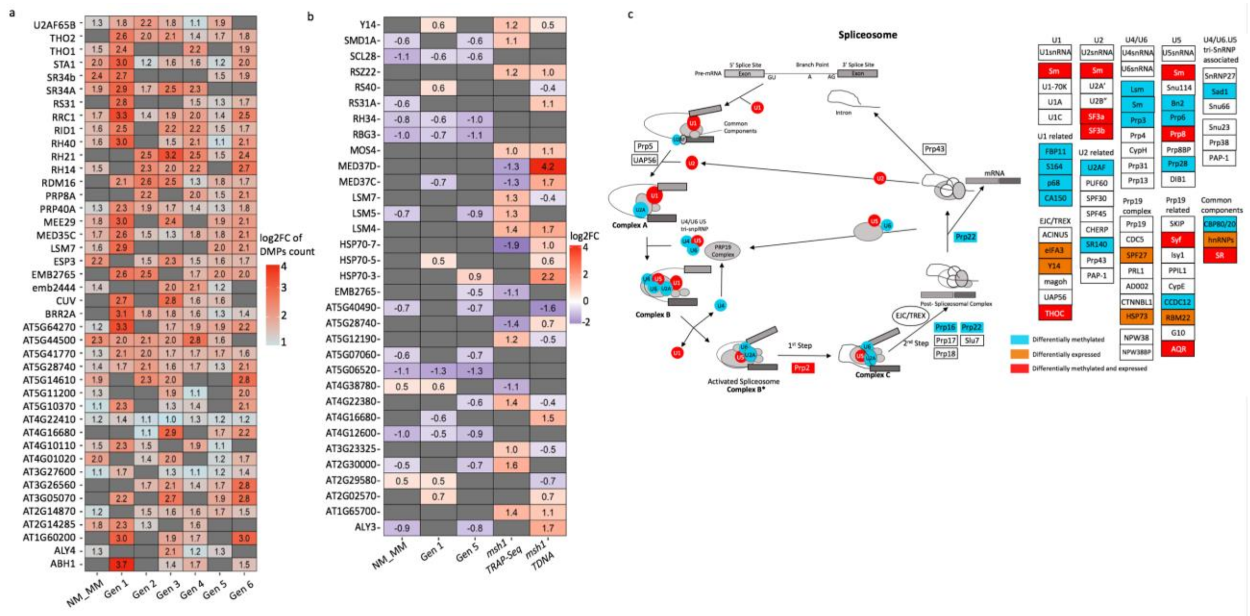

Supplementary Fig. 12. Differential expression and methylation within the spliceosome network in *msh1* memory. **a**, Identified DMGs from the spliceosome (ath03040) network in *msh1* memory line. Only genes identified as DMGs in at least 4 of 7 comparisons (gen1 to gen6 WT vs. MM and gen1 NM vs MM) are presented. The scale represents the log2 fold change of DMP number at specific loci in each comparison. **b**, DEGs from the spliceosome (ath03040) network in *msh1* memory line and *msh1* mutant. Only genes identified as DEGs in at least 4 out of 5 comparisons (Gen1 WT vs. MM, Gen1 NM vs. MM, Gen5 WT vs. MM, WT vs. *msh1* translatome (TRAPseq) and WT vs. *msh1* TDNA) are presented. **c**, DMGs and DEGs presented in panels **a** and **b** are positioned in the network, which is adapted from KEGG database (spliceosome - plant - Arabidopsis thaliana). In many cases, the names in boxes are a general gene group, and do not necessarily match the individual gene name in panels **a** and **b**. Boxes are colored if there is at least one gene in that group differentially expressed or methylated in our study. Source data are provided as a Source Data file.

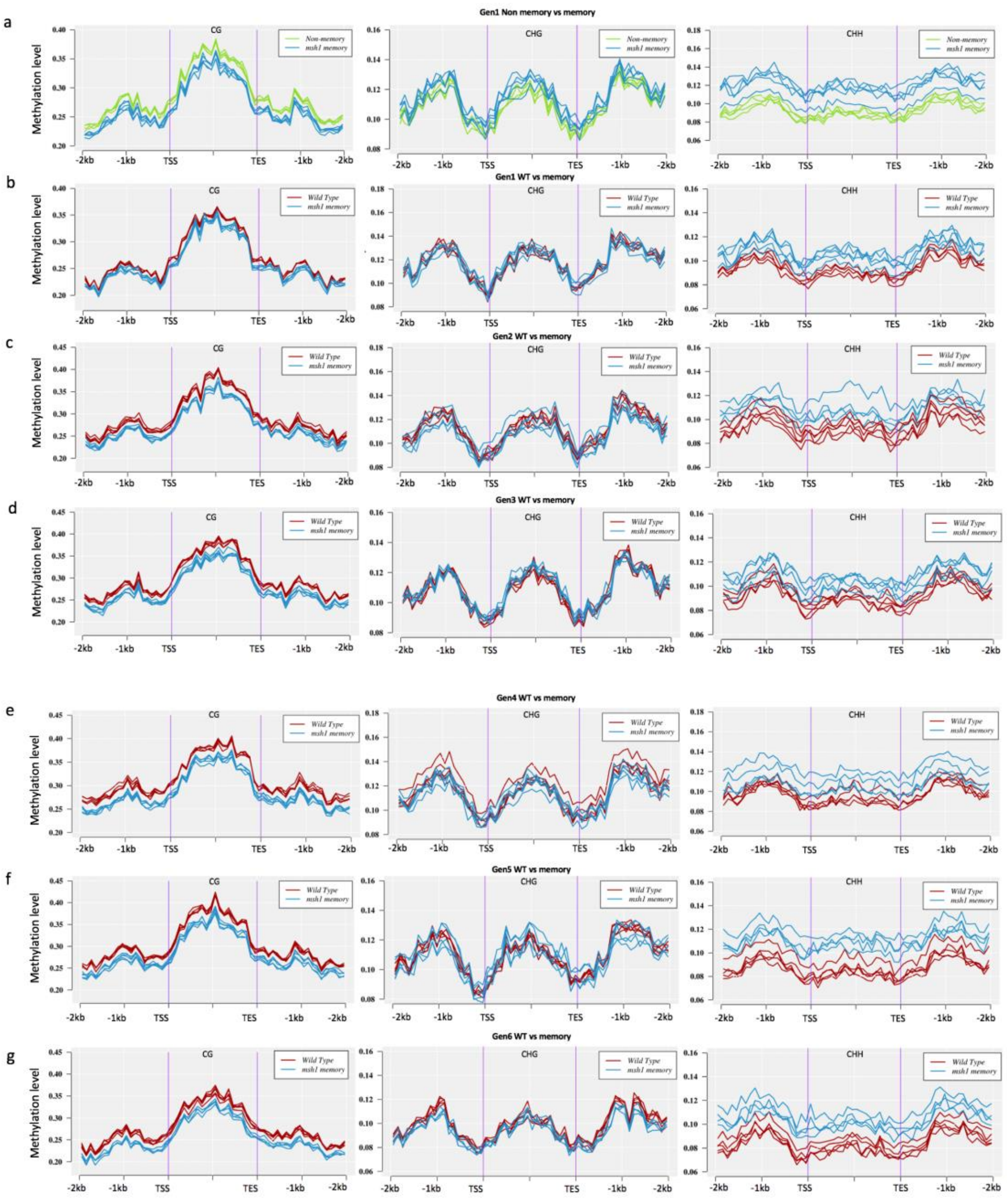

Supplementary Fig. 13. DNA methylation patterns of 954 heritable DMGs. Average methylation levels of all cytosines within 954 heritable DMGs at gene body, 2kb upstream of Transcription Start Site (TSS), and 2kb downstream of Transcription End Site (TES) were computed, divided to 60 bins and plotted. Gen1 non-memory vs memory (**a**), gen1 to gen6 WT vs memory (**b-g**), with each line representing a single plant. The source data associated with this figure is available on Expression Omnibus database dataset (accession number GSE118874 and GSE129303).

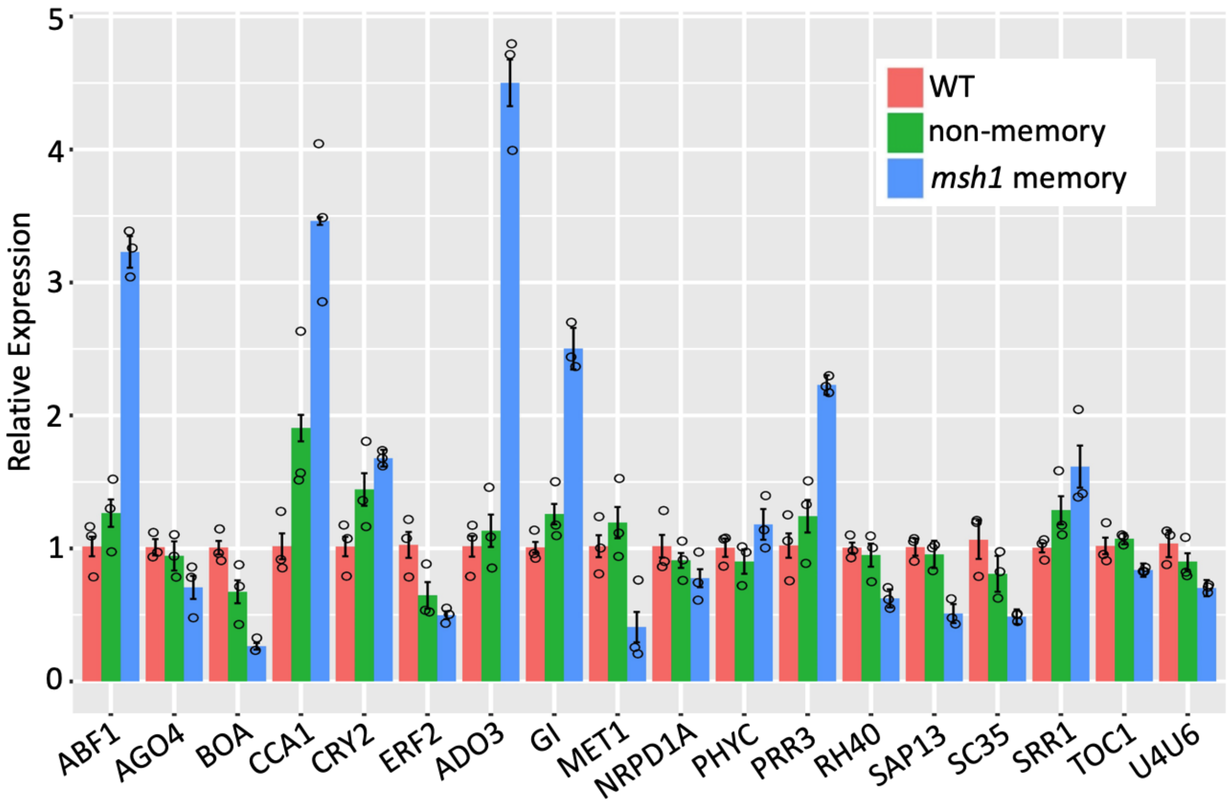

Supplementary Fig. 14. Relative expression of 18 circadian-, hormone-, and spliceosome-related genes assayed by qPCR. Relative expression was calculated by normalizing to wild type under corresponding treatment. Error bars represent mean ± SD of three independent biological replicates. Source data are provided as a Source Data file.

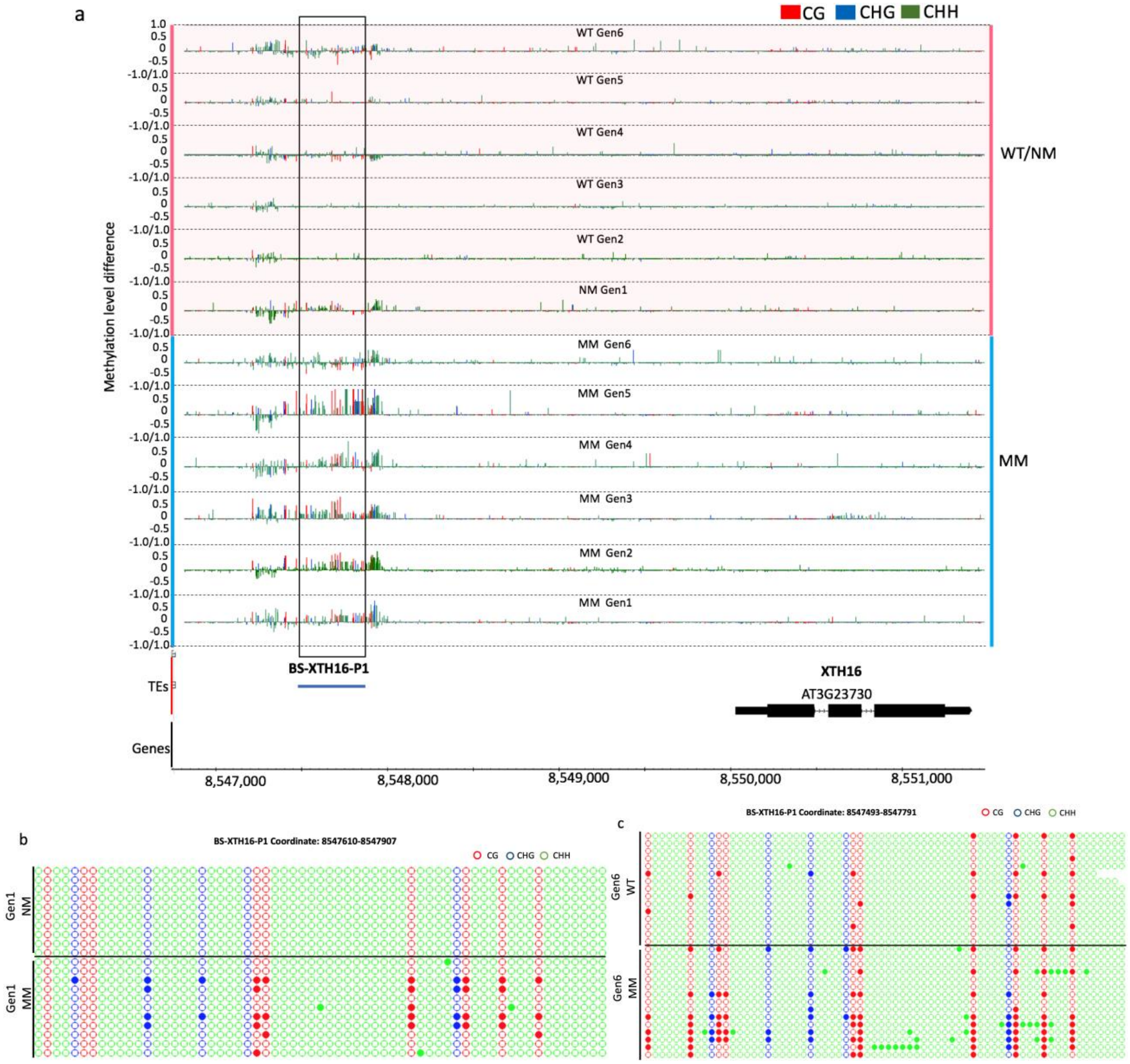

Supplementary Fig. 15. DMP calling by MethylIT was further confirmed by specific bisulfite-PCR sequencing. **a,** A region located in the promoter region of AT3G23730 showed substantial DMPs and general hyper-methylation in every generation of *msh1* memory (MM) lineage. The source data associated with this figure is available on Expression Omnibus database dataset (accession number GSE118874 and GSE129303). A primer set was used to amplify the region highlighted by black box (coordinate: 8547493-8547791). PCR results are presented in **b**, for first generation nonmemory (NM) and memory (MM) plants, and in **c** for the Gen6 WT and MM plants. Dot-plot analysis was applied to bisulfite sequencing results, where red, blue, and green circles represent CG, CHG and CHH respectively (methylation solid, no methylation blank). Each line represents one clone sequenced, and at least 10 clones for each sample in **b** and at least 15 for **c,** were sequenced for each sample. The gen6 wild type and *msh1* memory line plants were used in this experiment. The overall calculated conversion rate was 99.47% for WT and 100% for *msh1* memory.

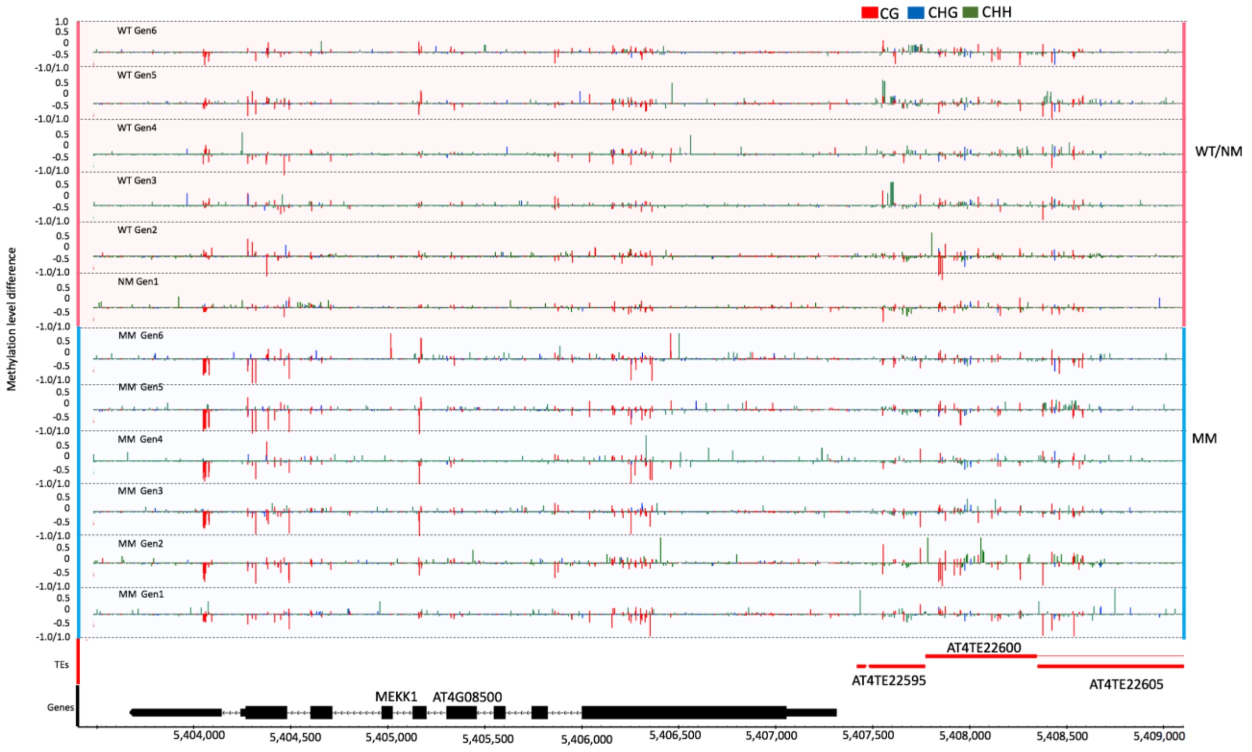

Supplementary Fig. 16. Methylation changes at *MEKK1* locus. Single cytosine methylation level changes in *msh1* memory line (MM) at the MEKK1 (AT4G08500) locus. Methylation level differences at each cytosine were computed by $(mC/(mC+uC))_{each\text{-}individual} - mC/(mC+uC)_{average\text{-}of\text{-}all\text{-}reference\text{-}plants}$, with mC denoting methylated cytosine and uC denoting unmethylated cytosine. For Gen1, non-memory (NM) plants were used as reference. For Gen2 to Gen6, wild type plants were used as the reference for each generation. Only one plant from each generation was selected as representative, so the pattern will differ slightly with different individuals selected due to fluctuation in methylation. Integrated Genome Browser (version 9.0.2) was used to generate the figure. The source data associated with this figure is available on Expression Omnibus database dataset (accession number GSE118874 and GSE129303).

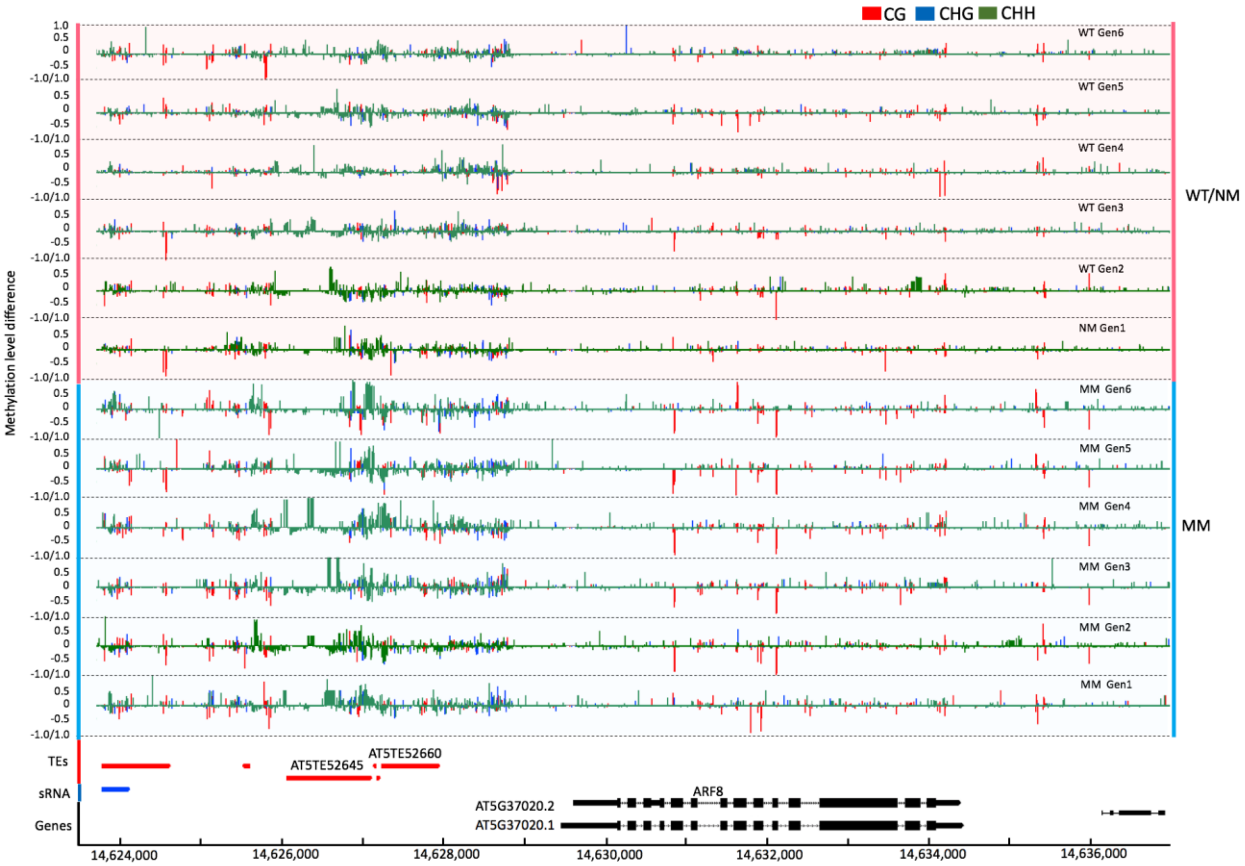

Supplementary Fig. 17. Methylation changes at *ARF8* loci. Single cytosine methylation level changes in *msh1* memory line (MM) in the *ARF8* (AT5G37020) locus. The source data associated with this figure is available on Expression Omnibus database dataset (accession number GSE118874 and GSE129303).

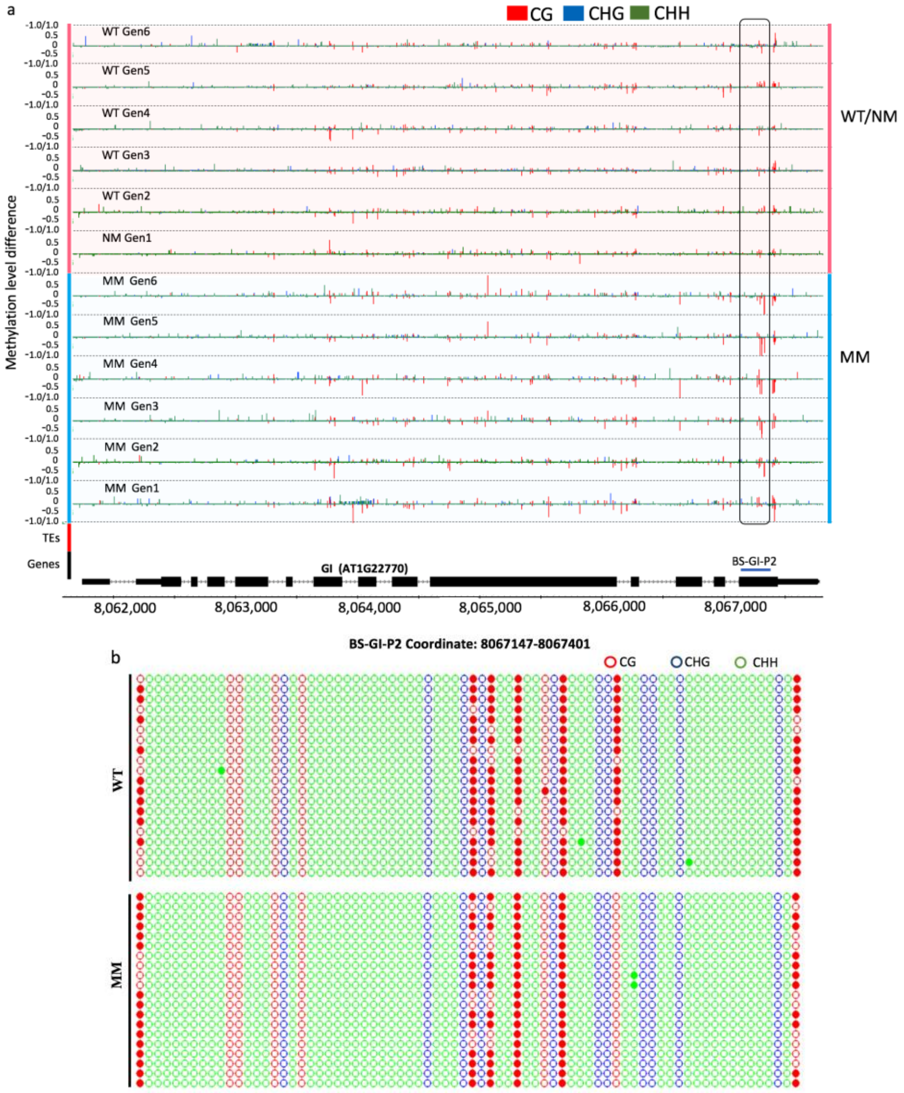

Supplementary Fig. 18. Methylation changes at the *GI* locus and DMP confirmation by bisulfite PCR. a, Single cytosine methylation level changes in the *msh1* memory line (MM) *GI* (AT1G22770) locus. The source data associated with this figure is available on Expression Omnibus database dataset (accession number GSE118874 and GSE129303). **b,** the methylation status of a predicted high DMP density region in *GI* was tested by bisulfite sequencing PCR, with primer binding site indicated in panel a (coordinate: 8067147-80674010). Dot-plot analysis was applied to bisulfite sequencing results, with red, blue, and green circles representing CG, CHG and CHH, respectively (methylation with solid, no methylation with blank). Each line represents one clone sequenced, with at least 15 clones sequenced for each PCR reaction. NM, non-memory.

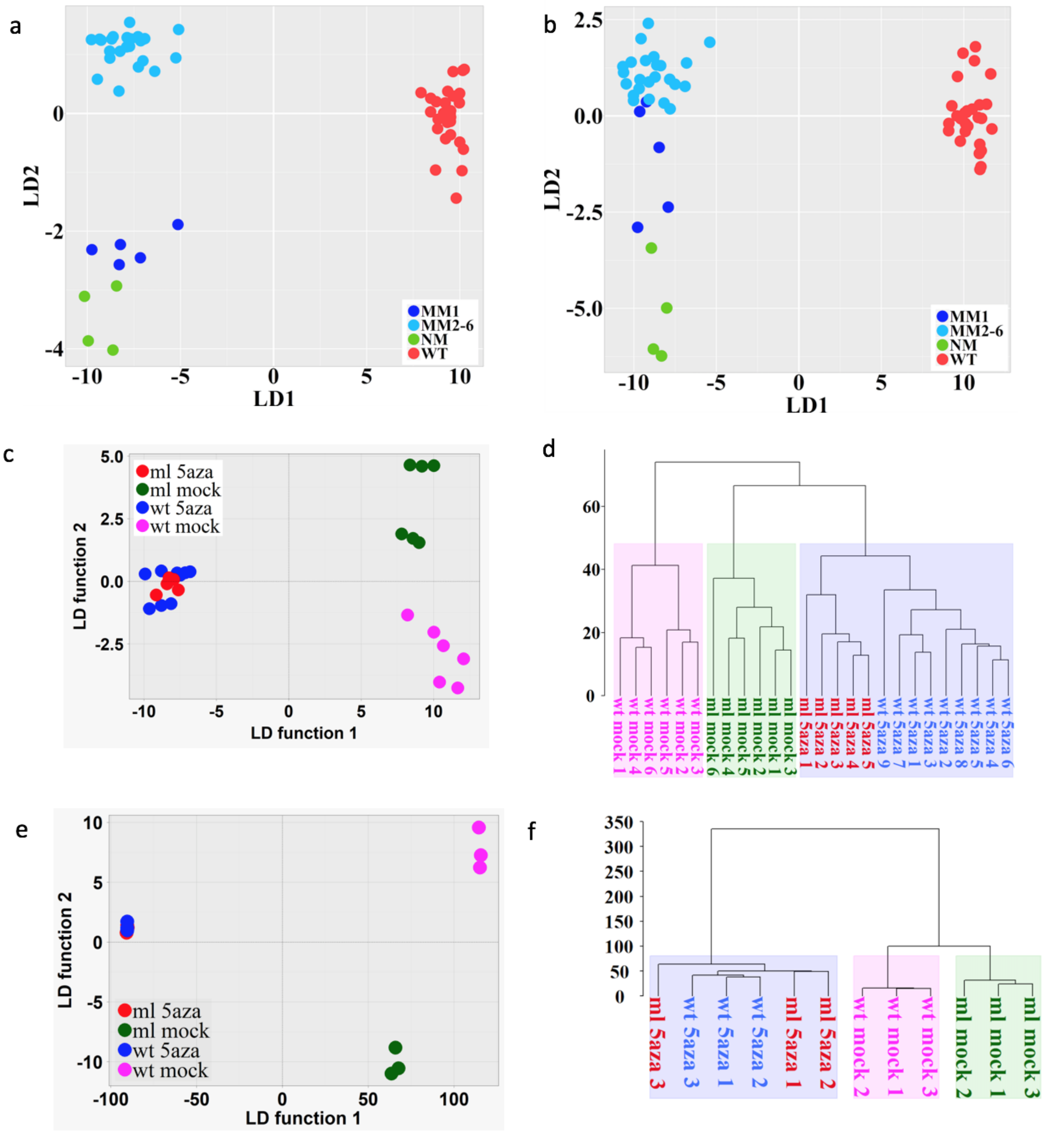

Supplementary Fig. 19. PCA and LDA analysis-based classification of *msh1* memory and wild type gene expression and methylation divergence. a, PCA+LDA analysis performed on 61 whole-genome bisulfite sequence datasets for *msh1* memory and wild type generation 1 through generation 6 samples. Each sample was represented as vector of absolute methylation difference for the 954-gene *msh1* memory line heritable DMG dataset. WT (wild type), NM (non-memory), M1 (*msh1* memory Gen1), M2-6 (*msh1* memory Gen2-Gen6). **b**, PCA+LDA analysis performed on 61 whole-genome bisulfite samples, with each sample represented as vector of absolute methylation difference for 373 DMGs (Supplementary Table 8), representing genes identified in 4 key networks of circadian rhythm (GO0007623), response to auxin (GO0009733), spliceosome (ath03040), and plant hormone signal transduction (ath04075). **c**, PCA+LDA performed for 26 samples on a set of 2660 differentially expressed genes (DEGs). A feature selection approach (see Methods) was applied to identify the 2660 genes with highest discriminatory power in all group comparisons. Individual samples were represented as vectors of log2-of-read-counts obtained from the RNAseq data. **d**, Hierarchical cluster for the 26 samples on the set of 2660 DEGs. **e**,

PCA+LDA analysis performed on 12 whole-genome bisulfite sequencing samples in the 5-azacytidine treatment experiment. Each sample was represented as vector of absolute methylation difference for 954 *msh1* memory line heritable DMGs identified in the *msh1* transgenerational memory bisulfite sequencing experiment. **f**, Hierarchical cluster for the 12 samples on the 954 DMGs. For c-f, wt (wild type), ml (*msh1* memory line), 5aza (5-azacytidine treatment). The source data associated with this figure is available on Expression Omnibus database dataset (GSE118874, GSE129303, GSE129343, GSE134028, GSE109164 and GSE114665). Detail description of Principal Components and Linear Discriminant Analyses with Methyl-IT are available at https://genomaths.github.io/.

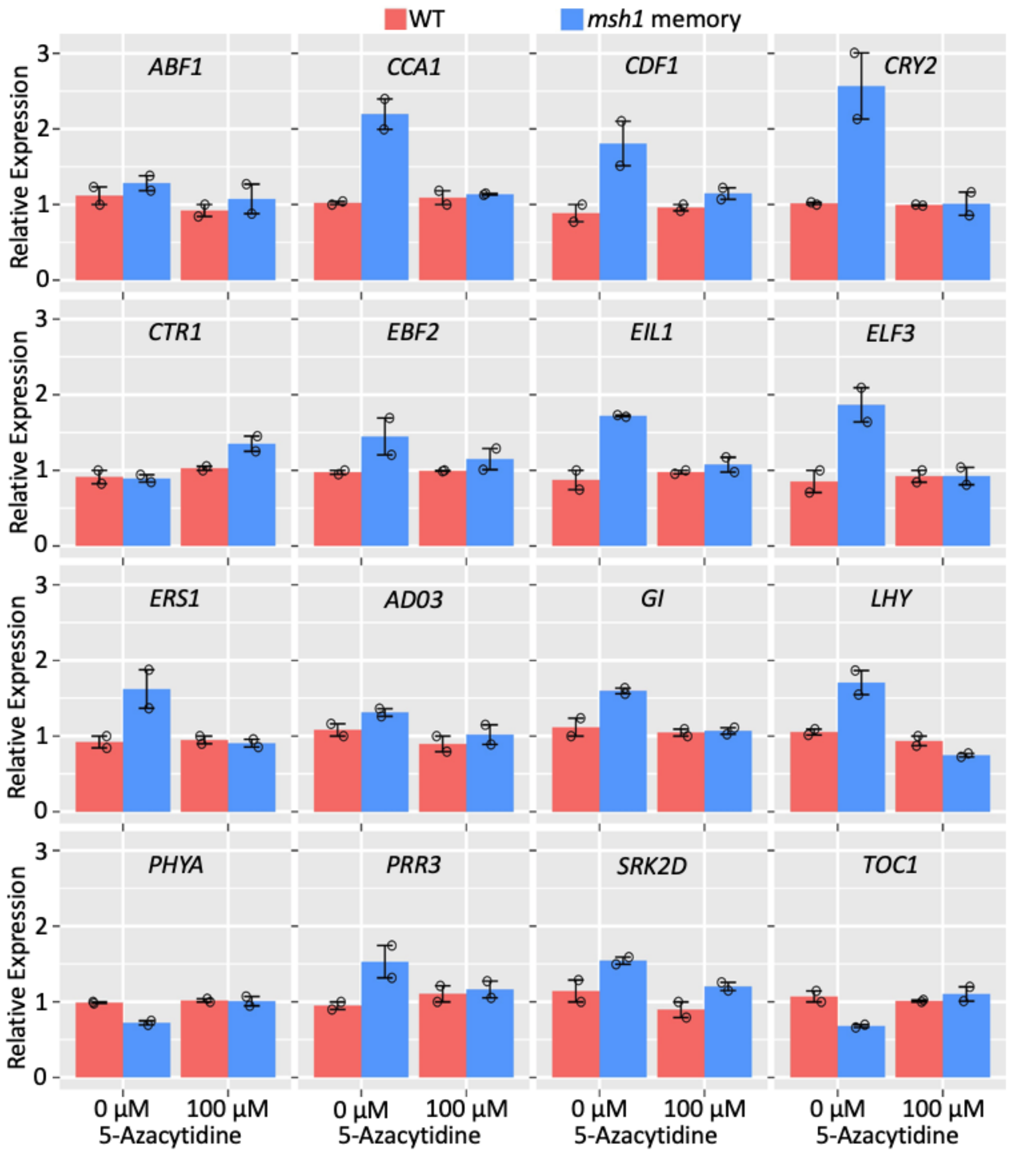

Supplementary Fig. 20. Expression of 16 circadian- and hormone-related genes following 5-azacytidine treatment. Relative expression of 16 genes was assayed by quantitative real-time PCR following 100 µM 5-azacytidine treatment. Relative expression was calculated by normalizing to wild type under corresponding treatment. Bar graph represents mean of two independent biological replicates. Source data are provided as a Source Data file.

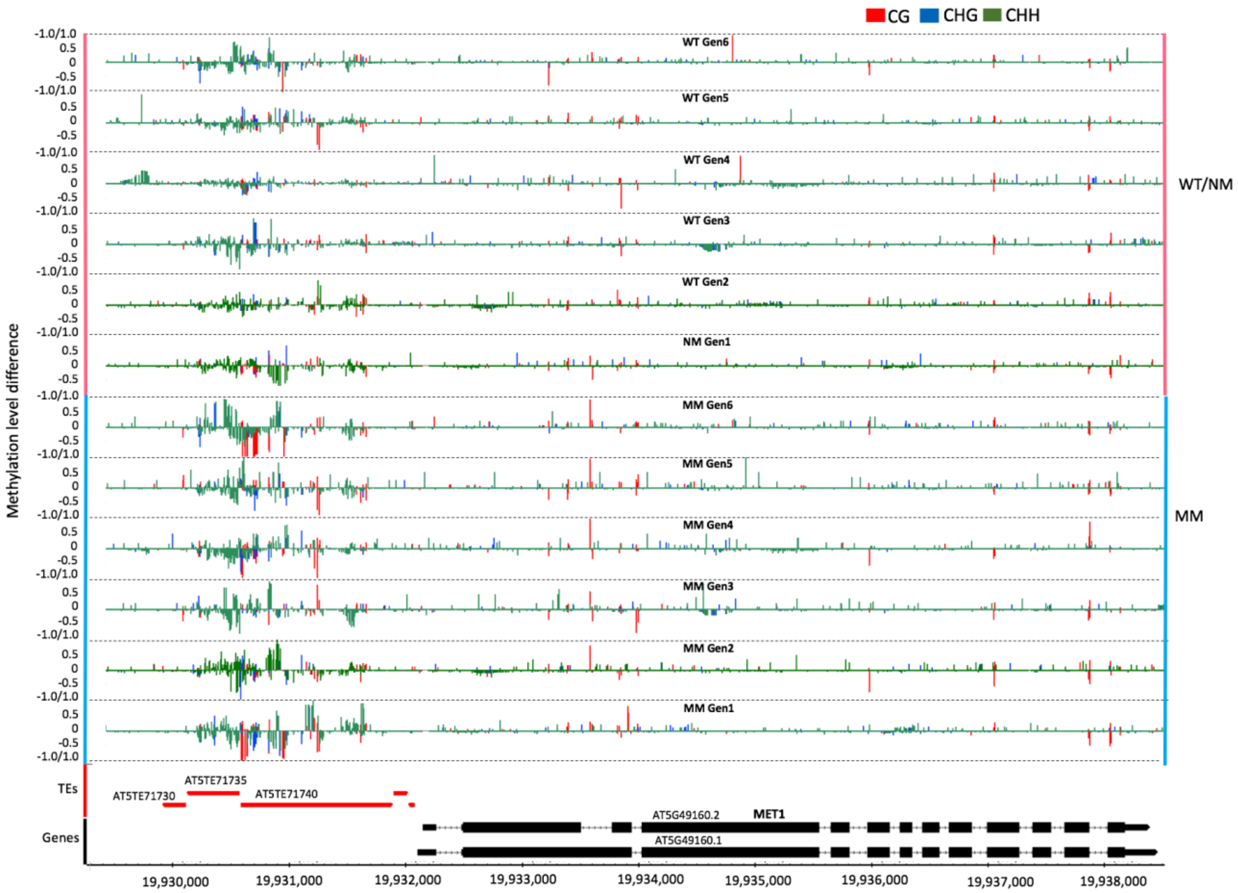

Supplementary Fig. 21. TE association with the methyltransferase gene *MET1*. Single cytosine methylation level changes in the *msh1* memory lineage (MM) for *MET1* (AT5G49160) locus and a neighboring TE (red). The source data associated with this figure is available on Expression Omnibus database dataset (accession number GSE118874 and GSE129303).

Supplementary References

1. Kuznetsova, A., Brockhoff, P. B. & Christensen, R. H. B. lmerTest package: tests in linear mixed effects models . *J. Stat. Softw.* **82,** (2017).
2. Schultz, M. D., Schmitz, R. J. & Ecker, J. R. 'Leveling' the playing field for analyses of single-base resolution DNA methylomes. *Trends Genet.* **28,** 583–585 (2012).
3. Sanchez, R., Yang, X., Maher, T., & Mackenzie, S. A. Discrimination of DNA methylation signal from background variation for clinical diagnostics. *Intl. J. Mol. Sci* **20**, 5343 (2019).
4. Kay, S. A. *et al.* Genome-wide identification of CCA1 targets uncovers an expanded clock network in Arabidopsis . *Proc. Natl. Acad. Sci.USA* **112,** E4802–E4810 (2015).
5. Kieffer, M., Neve, J. & Kepinski, S. Defining auxin response contexts in plant development. *Curr. Opin. Plant Biology* **13,** 12–20 (2010)
