# Supplementary material for: Segregation of an MSH1 RNAi transgene produces heritable non-genetic memory in association with methylome reprogramming
Source: Nat Commun. 2020 May 5;11:2214. doi: 10.1038/s41467-020-16036-8 (PMC7200659; doi:10.1038/s41467-020-16036-8)
Supplement: Supplementary file 3 — Reporting Summary [file 41467_2020_16036_MOESM3_ESM.pdf]

nature research

Corresponding author(s): Sally Mackenzie

Last updated by author(s): 04/03/2020

Reporting Summary

Nature Research wishes to improve the reproducibility of the work that we publish. This form provides structure for consistency and transparency in reporting. For further information on Nature Research policies, seeAuthors & Referees and theEditorial Policy Checklist .

Statistics

For all statistical analyses, confirm that the following items are present in the figure legend, table legend, main text, or Methods section.

n/a	Confirmed	
☐	☒	The exact sample size (*n*) for each experimental group/condition, given as a discrete number and unit of measurement
☐	☒	A statement on whether measurements were taken from distinct samples or whether the same sample was measured repeatedly
☐	☒	The statistical test(s) used AND whether they are one- or two-sided<br>*Only common tests should be described solely by name; describe more complex techniques in the Methods section.*
☐	☒	A description of all covariates tested
☐	☒	A description of any assumptions or corrections, such as tests of normality and adjustment for multiple comparisons
☐	☒	A full description of the statistical parameters including central tendency (e.g. means) or other basic estimates (e.g. regression coefficient) AND variation (e.g. standard deviation) or associated estimates of uncertainty (e.g. confidence intervals)
☐	☒	For null hypothesis testing, the test statistic (e.g. *F*, *t*, *r*) with confidence intervals, effect sizes, degrees of freedom and *P* value noted<br>*Give P values as exact values whenever suitable.*
☐	☒	For Bayesian analysis, information on the choice of priors and Markov chain Monte Carlo settings
☐	☒	For hierarchical and complex designs, identification of the appropriate level for tests and full reporting of outcomes
☐	☒	Estimates of effect sizes (e.g. Cohen's *d*, Pearson's *r*), indicating how they were calculated

Our web collection on statistics for biologists contains articles on many of the points above.

Software and code

Policy information about availability of computer code

Data collection	No software are used in the data collection
Data analysis	Numerous commercial programs were used and listed in Methods: SAMtools(version1.9), edgeR(version 3.10), Integrated Genome Browser(version 9.0.2),JRNA alignment (STAR (version 2.7.0a), sRNA analysis (ShortStack<br>version 3.8.3), RNA read count data (QoRTs software package (version v1.3.0)), RNA gene count normalization (DESeq2 (version 1.20.0).<br>Bisulfite sequence used FastQC (version 0.11.5), trimmed with TrimGalore! (version 0.4.1) and Cutadapt (version 1.15), aligned with<br>Bismark (version 0.19.0)71 with bowtie2 (version 2.3.3.1). Methylome analysis with R package DSS (version 2.30.1)<br>Custom Methyl-IT platform (R package Methyl-IT (version 0.3.1) at https://github.com/genomaths/MethylIT.

For manuscripts utilizing custom algorithms or software that are central to the research but not yet described in published literature, software must be made available to editors/reviewers. We strongly encourage code deposition in a community repository (e.g. GitHub). See the Nature Research guidelines for submitting code & software for further information.

Data

Policy information about availability of data

All manuscripts must include a data availability statement. This statement should provide the following information, where applicable:

- Accession codes, unique identifiers, or web links for publicly available datasets
- A list of figures that have associated raw data
- A description of any restrictions on data availability

Ensemble genomes database is available at http://ensemblgenomes.org/
KEGG database is available at https://www.genome.jp/kegg/kegg1.html
Source data are provided as a Source Data file.
All next-generation sequencing data generated by this study were deposited to Gene Expression Omnibus database under accession numbers listed:
Arabidopsis methylome for msh1 memory and non-memory sibling plants with isogenic Col-0 wild type control (GSE118874).

Arabidopsis msh1 memory 4-week-old plant RNAseq (GSE106536).
Arabidopsis 10 day old seedling 5-azacytidine treatment RNAseq (GSE109164).
Arabidopsis 10-day-old seedling 5-azacytidine treatment methylome (GSE114665)
Arabidopsis methylome for the generation 2 to 6 of msh1 memory line and isogenic Col-0 wild-type control: (GSE129303)
Arabidopsis RNAseq for the generation 1 and 5 of msh1 memory line and isogenic Col-0 wild-type control (GSE129343)
Small RNA sequencing of msh1 memory line and isogenic Col-0 wild-type control in Arabidopsis. (GSE134028)

Fig.3 associated with data GSE118874 and GSE129303
Fig.4 associated with data GSE118874
Fig.5-8 associated with data GSE118874, GSE129303, GSE129343, GSE134028
Supplementary Fig. 2-6,13,16-18,21 associated with data GSE118874 and GSE129303
Supplementary Fig. 7 associated with data GSE129343
Supplementary Fig. 8, 11-12 associated with data GSE118874, GSE129303 and GSE129343
Supplementary Fig. 19 associated with data GSE118874, GSE129303, GSE129343, GSE134028, GSE109164 and GSE114665

Field-specific reporting

Please select the one below that is the best fit for your research. If you are not sure, read the appropriate sections before making your selection.

- [x] Life sciences
- [ ] Behavioural & social sciences
- [ ] Ecological, evolutionary & environmental sciences

For a reference copy of the document with all sections, see nature.com/documents/nr-reporting-summary-flat.pdf

Life sciences study design

All studies must disclose on these points even when the disclosure is negative.

Sample size

Genetic studies ranged from ca. 100-400 determined to provide robust chi-square outcomes (limited by labor intensity)
5 azacytidine treatment plant growth, n=14 according to literature(Yang X. etal, Plant Physiol. 2015 May;168(1):222-32) .
Maintaining environment constant BS-seq memory population, 5 plants/generation/genotype for six generations. Standard in the field is 3 plants for BSseq analysis. Selection of five was to provide extremely robust and novel dataset for the community.
SRNA sequencing, 3 plants per genotype is sufficient to survey the sRNA population according to the literature(Polydore et al., The Plant Journal.2018;https://doi.org/10.1111/tpj.13919)

Data exclusions

excluded 4 out of 65 data from BSseq dataset (one from non-memory types ,4 other replicates remain, one from Gen1 wild type, 4 other replicates remain, one from gen 2 msh1 memory, 4 other replicates remain and one from gen6 msh1 memory, 4 other replicates remain)due to what appeared to be sequencing artifact that rendered data distinctly different pattern than the other four (Based on criteria defined by well accepted software, fastqc v0.11.2).

Replication

All experiments replicated at 2-3 experimental and multiple technical reps successfully as indicated in figure legends. Methylome analysis over six generations, 5 samples per generation, total 65 samples (4 out of 65 samples failed to get qualified data, see detail in data exclusions). All RNAseq and sRNA seq replicated minimum of 3 samples.(all successful)

Randomization

This is not relevant since we have conducted our experiments to assess phenotype-methylome- expression relationship.

Blinding

Lab scientists worked with plant identity and phenotype data; computational biologists worked blind with coded data.

Reporting for specific materials, systems and methods

We require information from authors about some types of materials, experimental systems and methods used in many studies. Here, indicate whether each material, system or method listed is relevant to your study. If you are not sure if a list item applies to your research, read the appropriate section before selecting a response.

Materials & experimental systems

n/a	Involved in the study	
x		Antibodies
x		Eukaryotic cell lines
x		Palaeontology
x		Animals and other organisms
x		Human research participants
x		Clinical data

Methods

n/a	Involved in the study	
x		ChIP-seq
x		Flow cytometry
x		MRI-based neuroimaging
