# Supplementary material for: Segregation of an MSH1 RNAi transgene produces heritable non-genetic memory in association with methylome reprogramming
Source: Nat Commun. 2020 May 5;11:2214. doi: 10.1038/s41467-020-16036-8 (PMC7200659; doi:10.1038/s41467-020-16036-8)
Supplement: Supplementary file 4 — Description of Additional Supplementary Files [file 41467_2020_16036_MOESM4_ESM.docx]

**Description of Additional Supplementary Files**

Name: Supplementary Data 1.

Description: Methylome data bisulfite conversion rate and sequencing statistics

Name: Supplementary Data 2.

Description: Networks identified by NEAT for NM vs MM and Gen1-6 WT vs MM

Name: Supplementary Data 3.

Description: Networks and genes in 1854 DMGs from Gen3 WT vs WT comparison by NEAT analysis.

Name: Supplementary Data 4.

Description: 4231 DMGs from WT VS MM GEN3 specific (excluding 1374 DMGs overlapped with WT vs WT)

Name: Supplementary Data 5.

Description: Total 5045 differentially expressed genes (DEGs) in the in the NM vs MM comparison

Name: Supplementary Data 6.

Description: Total 4509 differentially expressed genes (DEGs) in the Gen1 WT vs MM comparison

Name: Supplementary Data 7.

Description: Total 5777 differentially expressed genes (DEGs) in the Gen5 WT vs MM comparison

Name: Supplementary Data 8.

Description: The 954 DMGs retained through six consecutive generations and NM vs MM comparison

Name: Supplementary Data 9.

Description: Networks identified by NEAT for 954 heritable DMGs

Name: Supplementary Data 10.

Description: Differentially expressed sRNA cluster in the WT vs msh1 memory comparison

Name: Supplementary Data 11.

Description: 373 DMGs in key networks

Name: Supplementary Data 12.

Description: Differentially expressed genes (DEGs) in the WT vs *hda6-6* comparison

Name: Supplementary Data 13.

Description: Total 538 heritable DMGs (out of 954) overlapped with TEs within 1kb upstream and downstream
