# Supplementary material for: Segregation of an MSH1 RNAi transgene produces heritable non-genetic memory in association with methylome reprogramming
Source: Nat Commun. 2020 May 5;11:2214. doi: 10.1038/s41467-020-16036-8 (PMC7200659; doi:10.1038/s41467-020-16036-8)
Supplement: Supplementary file 2 — Peer Review [file 41467_2020_16036_MOESM2_ESM.pdf]

Reviewers' comments:

Reviewer #1 (Remarks to the Author):

This study by Yang, Sanchez et al attempts to explain the differences between sibling progeny of Arabidopsis plants that had expression of MSH1 reduced by an RNAi transgene. Several progeny of MSH1 RNAi plants exhibit a striking msh1 phenotype despite having not inherited any copy of the transgene and exhibiting normal MSH1 expression levels. The authors interpret this phenotype as a heritable stress memory, and focus on compared WT, "memory phenotype" plants and "non-memory phenotype" plants, both of which lack the RNAi transgene.
While multiple replicates and generations were sampled, there are several major flaws in the analysis and concerns with the conclusions drawn that in my view should clearly prevent this paper from being accepted for publication. In addition to the flaws outlined below, many of the experiments and conclusions presented are quite similar to the lab's previous paper on the topic (Virdi et al 2015), so it's also not clear to me whether the conclusions of this study represent sufficient novelty, even if the below concerns could possibly be addressed.

Major Concerns:

- MutS homolog (MSH) proteins are key enzymes in the mismatch repair pathway. Previous work by Mackenzie's group showed that MSH1 is localized to Chloroplasts and mitochondria, both of which encounter high levels of DNA damage and require DNA repair pathways to maintain stable genomes. It is not clear to me that a genetic basis for the very stably inherited phenotypes in MSH1 RNAi progeny has been ruled out. It seems likely that plants lacking normal mismatch repair capacity could accumulate mutations in organellar genomes, which could be passed on stochastically to progeny (depending on which individual organelles are passed on to the female germline). Rather, the authors seem keen to explain this phenomenon by examining changes in nuclear DNA methylation, even though the data poorly support this claim (see below).

- Most of the conclusions of this paper rely on methylation differences identified by two different methods. One of these methods (identifying DMRs) is widely used by many in the DNA methylation field. While not perfect, this method will generally identify the majority of methylation differences between two sample sets. It is troubling to see that there is a huge discrepancy between the results obtained by identifying DMRs, and the authors own method to identify differentially methylated cytosines (called methyl-IT). For example, the authors use fairly low stringency cutoffs in identifying DMRs (min. 3 CGs per window and p = <0.05), and yet still identify 0 CG DMRs between memory and non-memory plants! This suggests that the methylation differences between memory and non-memory plants are few and minor, yet their methyl-IT approach identified 7130 differentially methylated genes! This discrepancy cannot be brushed aside by simply claiming methyl-IT is more sensitive. Given such a wide discrepancy, the burden of proof should be on showing that the new method, methyl-IT, is able to accurately identify biologically meaningful differences, and it not over-fitting the data. The authors found that methyl-IT identified nearly 2000 differentially methylated genes between WT replicates, strongly suggesting that this method has a very high false positive rate and is not reliable. The size of methylation differences reported by this method appears to be very small (around 10%, Fig. 5A), and such differences could be easily created by small differences in coverage in the bisulfite-seq data. For example, a cytosine covered by 3 methylated reads and 3 unmethylated reads could be biologically identical to a sample covered by 3 methylated and 2 unmethylated reads, yet the two samples would exhibit a methylation difference of 10% (50% in the former versus 60% in the latter).

- There are too many additional concerns with the DNA methylation analysis and results presented to explain them all in depth, but together they lead me to conclude that the conclusions drawn from DNA methylation data are highly unreliable and likely unsound. Briefly, these concerns include: the majority of methylation differences not being inherited (e.g. Fig. 3B), the very small effect size of

most methylation differences (e.g. Fig. 5A), no scale to quantify the differences shown in Fig. 5C, single replicates shown for illustrative examples (e.g. Fig. 8), differences plotted instead of raw data (e.g. Fig. 8, Supp. Figures 11-14), single-cytosine changes being concluded to be biologically relevant (Supp. Fig. 14) and several results and bisulfite PCR confirmations being conducted on WT versus memory plants, instead of the more meaningful memory versus non-memory comparison (Fig. 4, Supp. Figures 11-14). All of the above concerns are troubling and suggest that the authors may be over-interpreting minor noisy differences between samples in their data.

- With all of the above technical concerns, I am also concerned that the authors' interpretations of their data largely goes against an emerging consensus within the field. In both mammals and plants, it is becoming increasingly clear that the expression of the vast majority of genes is not affected by even large changes to the DNA methylation state. In severe Arabidopsis mutants that lose almost all methylation, only 1,000-3,000 loci are typically affected (e.g. see Shook & Richards 2014), despite the majority of genes are proximal to at least one methylated region. The idea that thousands of very small effect changes to DNA methylation could have a meaningful effect on the transcriptome therefore goes against the majority of literature in the field.

- The authors' report that growing plants on 5-azacytidine alleviated phenotypic differences between WT and memory plants. However, my interpretation of this is that 5-aza is making both WT and memory plants equally sick, rather than reverting memory plants to a WT state. 5-azacytidine has drastic and pleiotropic effects on plant phenotypes and so is a very crude way of testing whether a phenotype is caused by methylation differences.

- Similarly the authors' over-interpret results from crosses between msh1 and hda6 or met1 mutants. Both hda6 and met1 mutants are very sick and exhibit pleiotropic phenotypes and genetic instability from disrupting almost all methylation and heterochromatin. The fact that double mutants with msh1 are almost all inviable simply suggests to me that the plants can't tolerate two mutations in very important processes. It is a major exaggeration to use these results to claim that met1 and hda6 play a role in the memory or inheritance of MSH1 RNAi phenotypes.

- Lastly, the authors report wide-ranging differences in gene expression, the most notable being differences in the expression of circadian clock genes. Such differences are often reported in diverse studies and are almost always simply explained by the RNA samples not having been collected at the same time of day. Indeed when the authors' sought to confirm these results with time-course qPCR experiments of WT and msh1 mutants (not memory and non-memory plants, which would have been a better comparison), they found mostly minor and unconvincing differences in the amplitude of circadian gene expression (Supp. Fig. 6).

Reviewer #2 (Remarks to the Author):

In this manuscript, Yang et al. dissect the role of MSH1 in epigenetic memory. In particular, they investigate the epigenetic changes occurring in msh1 mutant and how these changes are inherited transgenerationally only in 20% of the cases. By comparing the methylation and expression profiles in WT plants and memory and non-memory plants through 6 generations they are able to show how these changes are inherited stably. The paper is well written and presents interesting results. There are some major points that authors would need to address before I could recommend this paper for publication:

1. One of the main comments is regarding the magnitude of the changes in DNA methylation as reported by the authors. There is no explanation for the increase in methylation in generation 6 in all three contexts. The authors should explain or try to understand why that is the case. Are the bisulfite conversion rates similar for all samples?

2. Moreover, I would like to see an overlap of the DMRs through different generations. Are all DMRs from gen 2 present in gen 3 or not? If not why is that the case?
3. The authors used DSS to detect DMRs and due to its lack of sensitivity for small changes they switched to detect DMCs with Methyl-IT. Have the authors tried to detect DMRs with a different tool that would allow finer control of the DMRs parameters. For example, using DMRcaller (http://bioconductor.org/packages/DMRcaller/; https://doi.org/10.1093/nar/gky602) would allow them to call DMCs and merged them interactively into DMRs.
4. When the authors investigated the relationship between DMG and DEG, but they did not look where the changes in methylation take place. In other words, are DMG in promoter regions more predictive of DEG compared to changes in exons for example?
5. In the examples of individual loci (Figure 8 and the corresponding supplementary figures), the changes are not split per context. The authors need to color code the changes as per context to see if some of the difference observed are context specific or not.
6. The changes in methylation might be a consequence of the silencing of different methylation pathways in msh1 mutant. The authors need to try to disentangle which of the changes we see are direct consequence of MSH1 and which are downstream effects.
7. Interestingly, the authors found that, in msh1 mutant, components of RdDM pathway, but also MET1 and CMT3 are downregulated. While the former does not seem to have a role in this system, the latter is important and msh1/met1 double mutant leads to lethality. We know that MET1 and CMT3 are required to maintain CHG methylation through CCG sites followed by spreading (https://doi.org/10.1093/nar/gkw1330). Given that we know that genes with CmCG can regain methylation in ibm1 mutant, the authors should also look at the DMG genes and see if the have CmCG sites or not.

Small changes.
1. Figure 1d the colors are very similar between non-treated and treated plants. Different colors would make it easier to read the graph.
2. Figure 9ab, the color scheme is misleading. Use red to blue and white at zero.

Reviewer #3 (Remarks to the Author):

In this paper, the authors analyzed the unusual msh1 phenotype induced by RNAi knockdown of MSH1. Subsequent null segregation of the RNAi transgene results in plants that are restored for MSH1 expression but altered in phenotype, with delayed flowering, reduced growth rate, delayed maturity transition and pale leaves. First-generation memory versus non-memory full-sib comparison, combined with six-generation inheritance studies, identified gene-associated, heritable methylation repatterning that may associate with the phenotype. The genome-wide methylome analysis integrated with RNAseq and network-based enrichment studies identified altered gene networks and differentially methylated loci comprising these networks. Furthermore, it was suggested that the msh1 reprogrammed condition is dependent on functional HISTONE DEACETYLASE 6 and the methyltransferase MET1.

My major concern with the manuscript is that the molecular mechanism underline altered gene networks and differentially methylated loci is not defined. Furthermore, more evidence is required to support the authors’ claim that the msh1 reprogrammed condition was dependent on functional HISTONE DEACETYLASE 6 and the methyltransferase MET1. Since the authors were able to obtained the double mutants of msh1/met1, I suggest the authors can analyze the phenotype of the msh1/met1 double mutant to further investigate the relationship between the msh1 phenotype and MET1.

Other comments:

1. Supplementary Fig. 2

It appears that overall genome-wide methylation level was also higher among wild type individuals than non-memory line in Gen1 generation. Please explain.

2. Page 7. Line 155 – "DMRs were associated with 2520 unique genes in generation 1, predominantly in CG context, declining to 10-15% in subsequent generations, with slight increase in generation 6 (Fig. 3b, Supplementary Table 1).

In Figure 3b and 3c, Gen 2 shows more than 90% decrease compared to Gen 1. In contrast, Gen 6 shows 2-fold increase compared to Gen 5. Please explain.

3. Figure3b

Please explain what is NM_MM.

4. Page 14. Line 374 –"Figure 9c shows that double mutants between msh1 and components of the RdDM pathway displayed a phenotype very similar or identical to msh1 mutant alone.

Molecular evidence is required to confirm that double mutants between msh1 and components of the RdDM pathway display a phenotype very similar or identical to msh1 mutant alone. I suggest that the authors can further analyze the drm2/msh1 and dc12,3,4/msh1 mutants

5. Page 14. Line 375 –"The double mutant msh1/hda6 was not recoverable in segregating populations germinated on soil or nutrient media (Table 1). These data indicate that histone deacetylase HDA6 activity is required for initial msh1 reprogramming".

More evidence is required to support the claim that histone deacetylase HDA6 activity is required for initial msh1 reprogramming.

Yang et al., NCOMMS-19-13236-T, Response to reviewers' comments

Editor's comments:
we would require further mechanistic data to support the role of DMRs in in transgenerational and adaptive memory. In addition, as suggested by all three reviewers, the reliability of the DMRs identification methodology needs to be justified.

Response: We wish to express our gratitude to the reviewers for their very thorough and thoughtful review of our work. We have added considerable new data and greater detail, and we have attempted to clarify information to help the reader understand the methodology implemented in the study.
The new data include:

-Gen1 memory differentially expressed sRNA cluster and corresponding genes (Supple Table 10)
-addition of memory sRNA data association with DMGs identified in the study (integrated to Fig. 6b, Fig. 7, Fig. 9, Supple. Fig. 9, Supple. Fig. 15, Supple. Fig. 19)
-hda6 gene expression data (Supple. Table 11, reanalysis of raw data from Yu et.al, 2016)
-additional clarification regarding *hda6* and *msh1* gene expression intersections (in text and integrated to Fig. 6b, Fig. 7b, Fig. 9b, Supple. Fig. 5, Supple. Fig. 9, Supple. Fig. 10)
-addition of *drm2* MSH1-RNAi data to elaborate RdDM influence on memory transition (in text and Fig. 9).
-association between gene expression and methylation (Supple. Fig. 6).
-addition of data addressing cytosine context in the methylome repatterning of memory lines, and showing marked increase in CHH methylation with slight decreases in CG methylation in identified memory-associated DMGs (Supple. Fig. 11).
-addition of further analysis of memory gen6 methylation changes (Supple. Fig. 5b, Supple. Fig. 11).
-citation of a newly accepted publication from our group on the Methyl-IT method.
-editing of Results and Discussion for clarity.

Reviewer 1:
This study by Yang, Sanchez et al attempts to explain the differences between sibling progeny of Arabidopsis plants that had expression of MSH1 reduced by an RNAi transgene. Several progeny of MSH1 RNAi plants exhibit a striking msh1 phenotype despite having not inherited any copy of the transgene and exhibiting normal MSH1 expression levels. The authors interpret this phenotype as a heritable stress memory, and focus on compared WT, "memory phenotype" plants and "non-memory phenotype" plants, both of which lack the RNAi transgene.
While multiple replicates and generations were sampled, there are several major flaws in the analysis and concerns with the conclusions drawn that in my view should clearly prevent this paper from being accepted for publication. In addition to the flaws outlined below, many of the experiments and conclusions presented are quite similar to the lab's previous paper on the topic (Virdi et al 2015), so it's also not clear to me whether the conclusions of this study represent sufficient novelty, even if the below concerns could possibly be addressed.

Response: We wish to make two points of clarification here. First, we do not evaluate several progeny of MSH1 RNAi plants, we investigate the progeny of <u>one</u> single RNAi plant. This is an important distinction, because all of the memory-nonmemory lines that we have analyzed derive *from the same parentage*, so that there is no reasonable explanation for differences in methylome that correspond with phenotype unless they are related. This is as close as one can come to a linkage disequilibrium test

for nongenetic variation. These observations also exclude organellar origins for this phenotype-associated variation, unless it is organellar sorting that is directly causative of the methylome changes.

Secondly, there is no overlap between the work presented in this manuscript and that in Virdi et al. 2015. We presented no data on memory-nonmemory, memory methylome, or the underlying genetics of the system in Virdi et al. Moreover, we had not yet developed the Methyl-IT method in 2015, and were not able to include that level of resolution on methylome analysis. Therefore, we have re-analyzed *msh1* mutant methylome data here in order provide resolution that was lacking in the 2015 work.

Major Concerns:

- MutS homolog (MSH) proteins are key enzymes in the mismatch repair pathway. Previous work by Mackenzie's group showed that MSH1 is localized to Chloroplasts and mitochondria, both of which encounter high levels of DNA damage and require DNA repair pathways to maintain stable genomes. It is not clear to me that a genetic basis for the very stably inherited phenotypes in MSH1 RNAi progeny has been ruled out. It seems likely that plants lacking normal mismatch repair capacity could accumulate mutations in organellar genomes, which could be passed on stochastically to progeny (depending on which individual organelles are passed on to the female germline). Rather, the authors seem keen to explain this phenomenon by examining changes in nuclear DNA methylation, even though the data poorly support this claim (see below).

Response: We have shown in our previous work that msh1 participates in recombination surveillance. We have no evidence of mismatch repair by msh1 in the organellar genomes (this work is published in Davila et al. 2011, Xu et al. 2011 as cited). To hypothesize that memory phenotype is merely the consequence of organellar genetic variation induced by random mutation, one would need to assume (1) that differences could be observed in reciprocal crosses to wild type; they are not (Xu et al. 2012; Virdi et al. 2015); (2) that the organellar mutation would be discernable by mitochondrial and chloroplast genome sequencing following removal of the transgene, particularly because this mutation would be assumed to arise by aberrant recombination; this is not observable (Virdi et al. 2015; Xu et al. 2011). (3) One would not expect to observe evidence of enhanced growth fitness that is reversible by generation 6 simply by crossing an organellar mutation reciprocally; this is consistently the case in all species tested (Virdi et al 2015; Santamaria et al. 2014; Yang et al. 2015; Kenchanmane et al. 2018). Therefore, data to date are not consistent with the hypothesis of an organellar genetic lesion underlying msh1 behavior. However, we have not formally eliminated the possibility that plastid physiological (redox) effects may accompany memory and may contribute to its sustainability. This is a subject of current investigation that reaches beyond the scope of the current report.

- Most of the conclusions of this paper rely on methylation differences identified by two different methods. One of these methods (identifying DMRs) is widely used by many in the DNA methylation field. While not perfect, this method will generally identify the majority of methylation differences between two sample sets. It is troubling to see that there is a huge discrepancy between the results obtained by identifying DMRs, and the authors own method to identify differentially methylated cytosines (called methyl-IT). For example, the authors use fairly low stringency cutoffs in identifying DMRs (min. 3 CGs per window and p = <0.05), and yet still identify 0 CG DMRs between memory and non-memory plants! This suggests that the methylation differences between memory and non-memory plants are few and minor, yet their methyl-IT approach identified 7130 differentially methylated genes! This discrepancy cannot be brushed aside by simply claiming methyl-IT is more sensitive. Given such a wide discrepancy, the burden of proof should be on showing that the new method, methyl-IT, is

able to accurately identify biologically meaningful differences, and it not over-fitting the data. The authors found that methyl-IT identified nearly 2000 differentially methylated genes between WT replicates, strongly suggesting that this method has a very high false positive rate and is not reliable.

Response: One reason that we do not observe the same trends in DMR versus DMG analysis is the different means of establishing a cut off in the two methods. In DMR analysis, one cut off is applied to all generations in keeping with accepted convention. In DMG (Methyl-IT) analysis, we use a more sophisticated approach to establish the cut off that maximizes discrimination power of DMPs. Thus, each generation has its own optimized cut point to define DMPs.
We estimate the optimized cut point based on signal detection and classification to maximize the discrimination power of DMPs (a full elaboration is at https://genomaths.github.io/Cutpoint_estimation_with_Methyl-IT.html). To further confirm the discrimination power or accuracy of DMP calling in each generation, we divided DMPs into two groups: a training set (accounting for 60% of total DMPs) and testing set (accounting for 40% of total DMPs). A machine learning algorithm is applied to the training set, followed by performance of classification on the testing set. The performance of classification is verified by a cross check with sample ID. In this study, DMPs from all memory vs non-memory and WT vs memory comparisons achieved False Discovery Rate (FDR) <0.05 and accuracy > 90% with 500 Monte Carlo sampling (Gen6 WT vs memory showed a slightly lower accuracy; Supplementary Fig. 3b). These approaches provide strong evidence for our ability to predict memory-associated methylation changes. We also confirmed DMP calling by empirical testing (Supplementary Fig. 13, Supplementary Fig. 16).

The reviewer is quite right that the Methyl-IT method is measuring methylation using a different approach than the standard DMR analysis. We would argue, however, that this method was developed precisely because the previous DMR methods did not, in our view, provide adequate resolution of biologically meaningful differential methylation signal beyond high density silencing marks, residing largely within TE and noncoding regions. It should be noted that much of our analysis here did not rely exclusively on Methyl-IT; we detect methylation differences between memory and wild type without the use of Methyl-IT. We have conducted conventional DMR analysis and have identified related pathways, but with very low resolution. One of the key limitations of conventional DMR analysis is that it does not sufficiently address background variation existing within both treatment and control samples. The surprise expressed by this reviewer in our identification of DMGs among the wild types, which we show to be consistent with expectations based on growth stage, is that the DMR methods commonly used simply do not supply this information. Use of signal detection, and treatment of wild type and memory methylome datasets as overlapping distributions, permits us to resolve memory-specific methylome DMPs without the application of arbitrary parameters that dull signal to noise ratio. We have applied this methodology to progeny from a single parent, using 5 samples rather than the 2 that is standard in the field, and we have incorporated numerous tests of our data that go far beyond what is common to the literature. In these ways, we believe that we provide an unequivocally robust dataset.

-The size of methylation differences reported by this method appears to be very small (around 10%, Fig. 5A), and such differences could be easily created by small differences in coverage in the bisulfite-seq data. For example, a cytosine covered by 3 methylated reads and 3 unmethylated reads could be biologically identical to a sample covered by 3 methylated and 2 unmethylated reads, yet the two samples would exhibit a methylation difference of 10% (50% in the former versus 60% in the latter).

Response: Firstly, 10% methylation differences genome-wide is a significant difference. In the case of Fig.5A, each column represents at least 41 million cytosine sites, so that the example given by the reviewer would be irrelevant here. But we do agree with the reviewer that 10% at a single locus is not significant, which is why we used 20% methylation difference and minimum 8 coverage as the cut off in our DMP analysis. These details are found in the Methods section. We have added Supplementary Fig 11 to provide a visualization of how memory line differs from non-memory at gene regions (954 DMGs). The hypo-methylated CG and hyper-methylated CHH patterns clearly suggest that the difference detected between memory and non-memory is biologically meaningful and not accounted for by sequencing coverage differences.

- There are too many additional concerns with the DNA methylation analysis and results presented to explain them all in depth, but together they lead me to conclude that the conclusions drawn from DNA methylation data are highly unreliable and likely unsound. Briefly, these concerns include: the majority of methylation differences not being inherited (e.g. Fig. 3B), the very small effect size of most methylation differences (e.g. Fig. 5A), no scale to quantify the differences shown in Fig. 5C, single replicates shown for illustrative examples (e.g. Fig. 8), differences plotted instead of raw data (e.g. Fig. 8, Supp. Figures 11-14), single-cytosine changes being concluded to be biologically relevant (Supp. Fig. 14) and several results and bisulfite PCR confirmations being conducted on WT versus memory plants, instead of the more meaningful memory versus non-memory comparison (Fig. 4, Supp. Figures 11-14). All of the above concerns are troubling and suggest that the authors may be over-interpreting minor noisy differences between samples in their data.

Response: We intentionally use moderate stringency in our initial cutoff so that we can provide total transparency for the various steps of our analysis. It is important to note, however, that we include hyper- and hypo-methylation changes in our assessment, greatly increasing the DMP dataset. We do not suggest that all methylation changes observed are associated with memory and, therefore, heritable. We provide as complete a picture of the data as possible so that the reader can appreciate transgenerational methylome behavior and its natural stochasticity. This is a unique dataset that we believe to be extremely valuable to studies of natural transgenerational epigenomic behavior. The fact that only a proportion of the methylome variation is heritable should not be surprising to the reviewer; that is the nature of methylation variation if one accepts chromatin features as components in the regulation of system stochasticity (Joseph et al. 2015). The “very small size of most methylation differences” is, likewise, the nature of methylation variation when one is pooling heterogeneous cell types. Because we use Methyl-IT, we are able to visualize this variation where standard DMR analysis simply filters it out. We believe that there is value in this resolution and that discounting it as background noise without evidence can lead to biased analyses.

We appreciate the reviewer noting the lack of a scale in Figure 5C; this was an inadvertent error in final printing and we have included that scale in the revised version.

With regard to Fig. 8 and Supple. Figures 11-14, both methylation level and methylation difference are computed from raw cytosine counts. Therefore, we did use raw data. The reason that we have used methylation difference as well as methylation level is that methylation difference provides the direction of methylation changes (hyper, hypo) and information about individual variation, so is simply more informative. It is not physically feasible to plot all individuals, so we have selected one representative plant for each generation. But, to address this reviewer’s concern, we have added a new figure (Supple. Fig. 11) with all samples for every generation included.

We have included comparisons between memory and nonmemory as well as memory versus wildtype in order to make the point that the memory condition is discernable. One important outcome of this study is the opportunity to identify biomarkers for the epigenetic reprogramming phenomenon; consequently, the memory versus wildtype comparison is relevant.

- With all of the above technical concerns, I am also concerned that the authors' interpretations of their data largely goes against an emerging consensus within the field. In both mammals and plants, it is becoming increasingly clear that the expression of the vast majority of genes is not affected by even large changes to the DNA methylation state. In severe Arabidopsis mutants that lose almost all methylation, only 1,000-3,000 loci are typically affected (e.g. see Shook & Richards 2014), despite the majority of genes are proximal to at least one methylated region. The idea that thousands of very small effect changes to DNA methylation could have a meaningful effect on the transcriptome therefore goes against the majority of literature in the field.

Response: it is not possible for us to meaningfully address this comment regarding the work of others. To investigate the msh1 memory phenomenon, we have taken what we believe to be a methodical approach to understand what can only, to date, be explained as likely epigenetic effects. The approach that we have taken goes beyond what is standard to the field in sample numbers, sequence depth (RNA and methylome), number of generations, and inclusion of highly related materials to conduct experiments that are as well controlled as we believe have been conducted to date in studies of plant methylome behavior on a genome-wide scale. We do not make the assumption that methylation changes are causative to gene expression changes; in fact, we state clearly that we may be observing methylome changes that derive from perturbation of gene expression, a means of reestablishing homeostasis. It is quite possible (and our current lab working model) that memory initiates at the level of histone composition and modification, leading to methylation changes as a secondary effect. What we point out is that, regardless of causation, we observe striking correlation between gene networks altered in methylation and gene expression, and these correlations provide what we believe to be fundamentally important information regarding plant memory behavior. Many of these pathways are consistent with reports by others on shorter term memory and stress effects. We show that one cannot account for the observed heritability of gene expression and methylome data as random outcomes, and we cannot account for the correspondence of observed phenotypes with both methylome and gene expression unless a true relationship exists. We have added an additional supplementary figure (supple Fig. 6) to address this evidence of relationship. In this figure, we carried out a correlation analysis of DNA methylation divergence and gene expression divergence on 20,022 genes (total genes with methylation divergence >0) in gen1 *msh1* memory plants. A linear statistical association between |log_2 FC| and MD was confirmed with the application of a linear-by-linear association test (p-value << 0.0001), with the Spearman' s $\rho = \ -0.166$ for up-regulated genes and $\rho = -0.17$ down-regulated genes, indicating that gene expression and methylation processes are not independent in the memory line first generation (Supplementary Fig. 6a, b). We further investigated the impact of methylation change location and direction on gene expression. Using 821 genes that represent both DMGs and DEGs in generation 1 *msh1* memory, we identified significant difference between CG gene-body hypo-methylated and hyper-methylated DMG expression levels, with higher CG gene-body methylation level associated with lower gene expression level (Supplementary Fig. 6c). A significant difference was also seen between CHH promoter region hypo- and hyper-methylated DMG expression levels, such that higher CHH methylation in promoter regions associated with lower gene expression levels (Supplementary Fig. 6d)

- The authors' report that growing plants on 5-azacytidine alleviated phenotypic differences between WT and memory plants. However, my interpretation of this is that 5-aza is making both

WT and memory plants equally sick, rather than reverting memory plants to a WT state. 5-azacytidine has drastic and pleiotropic effects on plant phenotypes and so is a very crude way of testing whether a phenotype is caused by methylation differences.

Response: The reviewer makes a good point that it is difficult to assess all of the effects of 5-aza aside from its impact on methylation. This is, of course, why we include wild type, and why we include multiple measurements on growth following removal from 5-aza. We include these results because this is one (imperfect) standard in the field for testing association of phenotype effects, both visual and molecular, with DNA methylation. However, this is also why we have included a broad time course for growth to show that after plants are no longer being impacted by 5-aza (the chemical has a very short half-life) and have been removed to potting medium, the growth between wildtype and memory remain even. We accompany these data with both methylome and gene expression analysis to confirm that we have obviated the methylome and gene expression changes that characterized memory from wild type (Supplementary Fig. 17). I don't know how we could improve these experiments any further, and we believe that these data are important to underlining the multifaceted experimental approaches that were used to understand the memory state.

- Similarly the authors' over-interpret results from crosses between *msh1* and *hda6* or *met1* mutants. Both *hda6* and *met1* mutants are very sick and exhibit pleiotropic phenotypes and genetic instability from disrupting almost all methylation and heterochromatin. The fact that double mutants with msh1 are almost all inviable simply suggests to me that the plants can't tolerate two mutations in very important processes. It is a major exaggeration to use these results to claim that met1 and hda6 play a role in the memory or inheritance of MSH1 RNAi phenotypes.

Response: A genetic approach incorporating mutations within the pathways that control DNA methylation and chromatin remodeling seems to us to be the most powerful way to test for a relationship between *msh1* phenomena and epigenomic factors. We show below that, contrary to the reviewer's argument, the *hda6* mutant is quite healthy and shows no overt phenotype other than delayed flowering, and that *met1* is a manageable mutant as well. However, we have now added considerably more data to the genetic mutant section. We show that the pattern of altered gene expression in *hda6* shares 50% of its DEGs and 80% of its gene networks with the DEG and network datasets for *msh1* mutant and overlaps 34-44% with altered expression in memory generations (Supplementary Table 11). Together with the genetic data for *msh1/hda6* lethality, these data make a compelling argument for a relationship between HDA6 and MSH1 activities.

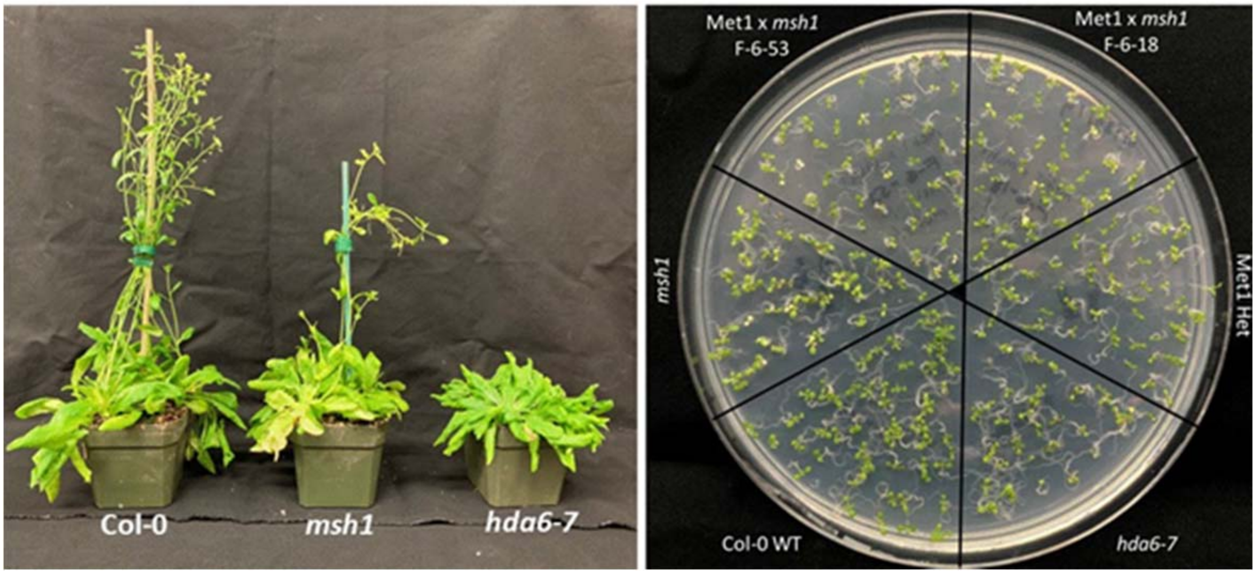

With regard to RdDM, we have now introduced the MSH1-RNAi construct to a *drm1/2* mutant; DRM1 and DRM2 participate as methyltransferases in maintaining CHH methylation through the RdDM pathway. Subsequent segregation of the RNAi transgene permitted testing for evidence of memory in progeny. We screened 547 progeny from a *drm1/2* MSH1-RNAi hemizygous plant, and found 170 transgene-null plants, each displaying no similarity with *msh1* memory phenotype (Fig.9d, e); we would expect 20% (34 plants) to show the *msh1* memory phenotype. These data appear consistent with newly added sRNA datasets in supporting the role of RdDM on memory methylation repatterning. We believe that these data greatly strengthen this section of the manuscript, and we thank the reviewers for prompting us to elaborate further.

- Lastly, the authors report wide-ranging differences in gene expression, the most notable being differences in the expression of circadian clock genes. Such differences are often reported in diverse studies and are almost always simply explained by the RNA samples not having been collected at the same time of day. Indeed when the authors' sought to confirm these results with time-course qPCR experiments of WT and msh1 mutants (not memory and non-memory plants, which would have been a better comparison), they found mostly minor and unconvincing differences in the amplitude of circadian gene expression (Supple. Fig. 6).

Response: The reviewer is correct that many of the gene expression changes that we observe, and the gene networks that they belong to, display circadian properties and we make this case in the manuscript. This is, of course, why all of our sampling is done at the same time of day from tissues

grown to the same stage of development (as well as can be approximated). Therefore, we are confident that the changes we observe, and demonstrate by RNAseq and multiple individual gene assays, are accurate and consistent. The data we show for the LD and LL time courses show significant, unambiguous differences with multiple replicates, so we are not sure why the reviewer considers these to be minor and unconvincing. These are the data that emerge from the memory phenotype in our hands, and I am not aware of any other assay that is more accurate than those we have implemented here. We think that it is important to point out, however, that the system that we are investigating is novel, and better approximates natural methylome and gene expression behaviors than one might observe with genetic mutants or plants under intense stress conditions, which comprises a large amount of the founding literature in both circadian and methylome studies. The fact that we do not observe extremely strong signals in many of our assays may simply be a reflection of the subtle nature of *msh1* memory.

Reviewer #2 (Remarks to the Author):

In this manuscript, Yang et al. dissect the role of MSH1 in epigenetic memory. In particular, they investigate the epigenetic changes occurring in msh1 mutant and how these changes are inherited transgenerationally only in 20% of the cases. By comparing the methylation and expression profiles in WT plants and memory and non-memory plants through 6 generations they are able to show how these changes are inherited stably. The paper is well written and presents interesting results. There are some major points that authors would need to address before I could recommend this paper for publication:

1. One of the main comments is regarding the magnitude of the changes in DNA methylation as reported by the authors. There is no explanation for the increase in methylation in generation 6 in all three contexts. The authors should explain or try to understand why that is the case. Are the bisulfite conversion rates similar for all samples?

Response: The reviewer raises an important question that we also found confusing. We had eliminated the possibility of bisulfite conversion rates (which are >99% each generation, and the average coverage per bp after alignment is about 20X in gen6, which is the same as other generations (Supplementary Table 3). We had also eliminated the possibility of variation in growth conditions. So, we have been conducting more detailed analysis of the genome-wide methylome features of gen 6. We do not detect differences in DMP and DMG numbers based on Methyl-IT analysis, suggesting that the “memory” features remain stable through each generation. The changes in question related to gen6 reside within

the DSS dataset for DMRs, and so imply a more general change in methylome behavior within gen 6. To assess this more fully, we examined probability density distribution of Methylation Divergence of memory gen 1 and memory gen 5 datasets, comparing their profiles with wild type gen 1 and gen 5 to reveal that they are consistent over generations, and quite distinct between memory and wild type. We then compared memory gen 6 with nonmemory to show that what appears to have occurred in gen 6 is a genome-wide patterning change to more closely resemble nonmemory. This observation, together with others we report in the Fig. 5 description of nonmemory, is consistent with the concept that some interchangeability exists between the memory and nonmemory states. However, analysis of the 954 memory-associated DMGs shows no such change in gen 6, implying that the identified memory "core" DMGs remain intact. A new supplementary figure (Supplementary Fig 11) has been added to address these new data. Clearly, future research by our group will focus on understanding this memory-nonmemory relationship more fully once we have developed a multi-generational lineage for nonmemory as well.

2. Moreover, I would like to see an overlap of the DMRs through different generations. Are all DMRs from gen 2 present in gen 3 or not? If not why is that the case?

Response: We have tracked the heritability of DMR-associated genes. Among 278 DMR-associated genes in gen2, 149 (53.6%) are DMR-associated genes in gen3. This is a significant amount of overlap in comparison to about 1% predicted with random sampling simulation.
However, through gen1 to gen6, there are only 13 DMR-associated genes shared. Our analysis suggests that only a relatively small proportion of DMRs/DMGs are heritable through all generations. In the case of DMR analysis, this could be due to technical details, since many cut-offs are applied to DMR identification, and adjusting these would produce better "heritability" of DMRs, but reduce likely biological relevance. Having identified 954 DMGs that are retained gen1 to gen6 by Methyl-IT, accounting for 13.8% of Gen1 DMGs, we believe that tracking methylation changes at the single-cytosine level is a better approach. We speculate that *msh1* memory as a heritable state is largely regenerated each life cycle, as we show in Fig.7, with many DMGs shared in multiple generations but not every generation. The slight difference in patterning and individual variation observed in Supplementary Fig 11 supports this speculation.

3. The authors used DSS to detect DMRs and due to its lack of sensitivity for small changes they switched to detect DMCs with Methyl-IT. Have the authors tried to detect DMRs with a different tool that would allow finer control of the DMRs parameters. For example, using DMRcaller (http://bioconductor.org/packages/DMRcaller/; https://doi.org/10.1093/nar/gky602) would allow them to call DMCs and merged them interactively into DMRs.

Response: We have compared more than one currently available method for DMR calling (BiSeq, methylpy, DSS), and opted for DSS because it is common within the plant literature and is relatively user-friendly, so that our results could be directly reproduced by any reader. One of the problems we faced in this earlier, unpublished preliminary study, was that DMR outputs for the three DMR methods overlapped only slightly, largely because of varying DMR criteria and over-stringent filtering. We are, therefore, not convinced that the lack of resolution is simply the consequence of DMR parameters. We developed Methyl-IT to address the limitations we have found with many programs that presume that "true" methylation signal is a function of its magnitude and density. We aimed to visualize all methylation variation that resides outside the wildtype control distribution, and to implement signal

detection to discriminate signal from background noise with little regard to DMP density or magnitude. In this way we are able to observe as much variation as possible that discriminates the treatment samples from control, which is particularly relevant when control is non-memory full sibs from the same parentage. We recognize that the analysis presented here is distinct from what has been conventionally used, and so we have employed an uncommonly robust dataset to validate our data. It would certainly be interesting to compare the Methyl-IT output with a number of additional methylation analysis procedures, but that is not our focus in this study.

4. When the authors investigated the relationship between DMG and DEG, but they did not look where the changes in methylation take place. In other words, are DMG in promoter regions more predictive of DEG compared to changes in exons for example?

Response: As suggested by the reviewer, we carried out a correlation analysis of DNA methylation divergence and gene expression divergence on 20,022 genes and describe this approach above in our response to Reviewer 1. We have also attempted to provide examples of the variation we observe in DMG patterning within a DEG, involving both promoter effects (and likely TE neighboring effects) as well as intragenic methylation(Fig. 8, Supplementary Fig. 15, Supplementary Fig. 16,Supplementary Fig. 19), and we have tried to make clear in the text that we cannot presume that the methylation we observe is necessarily causative of gene expression changes, merely related. It is entirely possible (and likely) that some methylation changes influence gene expression, while some methylation changes may be response to gene expression changes occurring in the parental line, likely during reestablishment of homeostasis. Therefore, we provide summary data on a genome-wide scale, but these studies will need to be followed by much more detailed investigation of chromatin changes occurring at identified memory-associated loci as well as detailed transcript splicing datasets to resolve this question more fully.

5. In the examples of individual loci (Figure 8 and the corresponding supplementary figures), the changes are not split per context. The authors need to color code the changes as per context to see if some of the difference observed are context specific or not.

Response: we are in total agreement and have now added this information. Beyond this, however, we have now added more information on the nature (context) of repatterning that discriminates memory from nonmemory, and we show that CHH hypermethylation and more minor CG hypomethylation appear to be important. Therefore, we have elaborated on this concept to provide what we consider a slightly more comprehensive picture of memory effects.

6. The changes in methylation might be a consequence of the silencing of different methylation pathways in msh1 mutant. The authors need to try to disentangle which of the changes we see are direct consequence of MSH1 and which are downstream effects.

Response:
In Supplementary Fig. 6, we show that a large proportion of DEGs in the memory line are CCA1/TOC1 binding genes (37.9% in Gen1 and 40% in Gen5), and selected groups of these genes are also presented. It is reasonable to assume that the expression changes observed in pathways such as starch metabolic process, response to ethylene, response to abscisic acid and response to cold are the consequence of changes in circadian clock network in the memory line. Fig 7 and Supplementary Fig.6 provide evidence

of alteration in both expression and methylation of circadian rhythm genes, and we suggest that circadian clock response is a likely direct consequence of MSH1 based on these data.We now add more clarification to the result section. We have also added additional data to indicate a role of RdDM processes in the memory transition, and to further support our observation that HDA6 participates in the msh1 reprogramming phenomenon.

7. Interestingly, the authors found that, in msh1 mutant, components of RdDM pathway, but also MET1 and CMT3 are downregulated. While the former does not seem to have a role in this system, the latter is important and msh1/met1 double mutant leads to lethality. We know that MET1 and CMT3 are required to maintain CHG methylation through CCG sites followed by spreading (https://doi.org/10.1093/nar/gkw1330). Given that we know that genes with CmCG can regain methylation in ibm1 mutant, the authors should also look at the DMG genes and see if they have CmCG sites or not.

Response: We have conducted further investigation of the influence of all three cytosine contexts in DMGs. In general, we observe that 954 DMGs show hypo-methylation in CG and hyper-methylated in CHH, but similar patterns in CHG to WT (Supplementary Fig 11). At the individual gene level, however, we observe substantial CHG change (Fig.8, Supplementary Fig. 11, Supplementary Fig 12, Supplementary Figure 17), although CHG seems to play a less significant role than CG and CHH in *msh1* memory. We have added additional information regarding RdDM influences on this system, by including sRNA data and *drm2* mutant data, and we have elaborated MET1/HDA6 information. We recognize that there are several layers of detail that will still require further dissection to understand the memory transition, nonmemory state, and the effects of the initial MSH1 suppression in conditioning progeny for memory. Our primary goal with this report is (1) to elaborate the novel, previously undescribed condition of msh1 memory, (2) to present a novel approach for methylome analysis that we believe will be useful in studies of “natural” methylome variation, and (3) to provide unambiguous evidence that the msh1 and memory phenomena involve epigenomic changes that align with, and inform us about, phenotypic plasticity and underlying stochasticity of epigenetic processes.

Small changes.
1. Figure 1d the colors are very similar between non-treated and treated plants. Different colors would make it easier to read the graph.

2. Figure 9ab, the color scheme is misleading. Use red to blue and white at zero.

Response: we have improved figure visuals as the reviewer suggests.

Reviewer #3 (Remarks to the Author):

In this paper, the authors analyzed the unusual msh1 phenotype induced by RNAi knockdown of MSH1. Subsequent null segregation of the RNAi transgene results in plants that are restored for MSH1 expression but altered in phenotype, with delayed flowering, reduced growth rate, delayed maturity transition and pale leaves. First-generation memory versus non-memory full-sib comparison, combined with six-generation inheritance studies, identified gene-associated, heritable methylation repatterning that may associate with the phenotype. The genome-wide methylome analysis integrated with RNAseq and network-based enrichment studies identified altered gene networks and differentially methylated loci comprising these networks. Furthermore, it was suggested that the msh1 reprogrammed condition is dependent on functional HISTONE

DEACETYLASE 6 and the methyltransferase MET1.

My major concern with the manuscript is that the molecular mechanism underline altered gene networks and differentially methylated loci is not defined. Furthermore, more evidence is required to support the authors' claim that the msh1 reprogrammed condition was dependent on functional HISTONE DEACETYLASE 6 and the methyltransferase MET1. Since the authors were able to obtained the double mutants of msh1/met1, I suggest the authors can analyze the phenotype of the msh1/met1 double mutant to further investigate the relationship between the msh1 phenotype and MET1.

Response: we are grateful for the reviewer's candid assessment of the genetics as previously presented. In response to this critique, we have now included additional cytosine context data on memory DMG methylation repatterning to reflect the marked CHH changes, data showing sRNA association with memory DMGs and *drm2* mutant influence on memory transition. We have also included more information on the *hda6* mutant and its likely intersection with *msh1* reprogramming networks. These data help clarify our hypothesis for processes underlying msh1 reprogramming and memory effects.

Other comments:

1. Supplementary Fig. 2

It appears that overall genome-wide methylation level was also higher among wild type individuals than non-memory line in Gen1 generation. Please explain.

Response: We hope that we have made clear in the text that the nonmemory state is not comparable to wildtype. Non-memory may resemble more closely the wild type control in plant phenotype and gene expression pattern, but non-memory is much more closely aligned with memory full sibs for methylome repatterning. This is to be expected, since they derive from the same parent as memory. Thus, non-memory is a wonderfully stringent (and unique) control for our studies of memory: we can assume that those methylome changes discriminating the two phenotypes, in plants deriving from a single parent, must be associated with phenotype. We have attempted to clarify this point in the text.

2. Page 7. Line 155 – "DMRs were associated with 2520 unique genes in generation 1, predominantly in CG context, declining to 10-15% in subsequent generations, with slight increase in generation 6 (Fig. 3b, Supplementary Table 1).

In Figure 3b and 3c, Gen 2 shows more than 90% decrease compared to Gen 1. In contrast, Gen 6 shows 2-fold increase compared to Gen 5. Please explain.

Response: Regarding the 90% DMR decrease in Gen 1 to Gen2, we state in the text, "we interpret the striking peak in generation 1 to represent variation inherited from the MSH1-RNAi parental type (>10,000 DMRs were identified in the *msh1* TDNA mutant) and memory transition effects." This transition is likely to be significant, and gens 2-6 likely represent a stabilization following transition. With regard to the increase in gen 6, we address this in a response to Reviewer 1, and Supplementary Fig 11 has been added.

3. Figure3b

Please explain what is NM_MM.
Response: Thank you. We have attempted to be consistent with use of NM as nonmemory and MM as memory. We have now added this clarification.

4. Page 14. Line 374 –"Figure 9c shows that double mutants between msh1 and components of the RdDM pathway displayed a phenotype very similar or identical to msh1 mutant alone.

Molecular evidence is required to confirm that double mutants between msh1 and components of the RdDM pathway display a phenotype very similar or identical to msh1 mutant alone. I suggest that the authors can further analyze the drm2/msh1 and dc12,3,4/msh1 mutants

Response: As suggested, we have investigated the relationship between the RdDM pathway and msh1 memory transition and this information has been described in an earlier response to Reviewer 1.

5. Page 14. Line 375 –"The double mutant msh1/hda6 was not recoverable in segregating populations germinated on soil or nutrient media (Table 1). These data indicate that histone deacetylase HDA6 activity is required for initial msh1 reprogramming".

More evidence is required to support the claim that histone deacetylase HDA6 activity is required for initial msh1 reprogramming.

Response: We have attempted to address this result in more detail by showing the striking intersection of *msh1* and *hda6* effects on gene expression, with particular reference to the networks of relevance to memory. The *hda6* mutant grows well and displays only a slight delay in flowering time as its most prominent change in phenotype, so we are confident that the double mutant lethality effect is relevant to the msh1 reprogramming and is important to include here.

Reviewers' comments:

Reviewer #1 (Remarks to the Author):

In this revised manuscript, Yang, Sanchez et al have provided multiple additional experiments and data points, but have failed to address the core concern of my previous review (also echoed by other reviewers), which is that the vastly different results obtained by their new analysis pipeline and methodology need to be fully justified in the context of both existing methylation analysis methods and the conclusions and emerging consensus of other studies in the field. Until the methodology of this paper is shown to be accurate, it is very hard to judge the biological significance of the authors' findings.

I shall therefore focus the majority of my review on questioning the authors' analysis methodology, beginning with a defense of existing DMR calling methods and why they were developed and adopted by most in the field in the first place.

Bisulfite-sequencing estimates the methylation level of individual cytosines by counting the number reads covering each cytosine in the genome, and comparing the number of converted (T) and unconverted (C) reads for each position. A cytosine covered by 10 converted and 10 unconverted reads would therefore have a methylation level of 50% across all cells in the sample. It is important to note that bisulfite-sequencing therefore has a considerable error in how accurately the "true" methylation level can be portrayed, which is exacerbated at lower read depths. For example, if a cytosine were covered by 2 converted and 4 unconverted reads in one sample (67% methylation level calculated), and 4 converted and 2 unconverted reads in a second sample (33% methylation level calculated), the two samples would display a methylation difference of 33%. However, statistically it is highly likely that these two samples have the same true methylation level, and that the sampling of reads at low depth has created a false positive.

In order to therefore claim that a methylation difference of 10% or lower (such as those reported in Figure 5a) can be accurately measured with statistical confidence, the authors' would therefore need extraordinary read depth. If a conventional statistical test such as the Chi squared were chosen, the read depth of an individual cytosine would need to be >200 to call a methylation difference of 10% with statistical significance.
As such read depths are largely unobtainable, various statistical methods have been developed to identify differentially methylation regions (DMRs) with greater confidence. These methods are not perfect, as they can still generate several false positive or negative results across a large genome, depending on stringency. DMR analyses typically enforce that cytosines must have large differences in methylation level (e.g. 35-40% for a CG dinucleotide) and that they pass thresholds of statistical significance using a test such as Fisher's exact test. Importantly, DMRs also identify regions of the genome where multiple cytosines in direct proximity show similar changes in methylation level, as this is much less likely to occur by chance than sampling a single cytosine. The reason for this is also based on biology – changes in the methylation of single cytosines have never been shown to have a biologically relevant effect in vivo, whereas changes to the methylation state of larger regions have been shown to affect expression of some genes and TEs in several species.

It is therefore troubling that the results of DMR analysis are dismissed by the authors as not sufficiently sensitive, without careful consideration as to why their sensitivity was purposefully developed to be stringent in the first place. From my understanding of the methods outlined in the PloS One methods paper by Sanchez and Mackenzie (although I am not a mathematician), and the information presented in this manuscript, Methyl-IT does not seem to take into account the inherent inaccuracy of bisulfite sequencing as a technology. It is commendable that the authors have generated data for several genetically identical replicates, but this can still lead to false positives if replicates were processed in batches (i.e. biases in PCR amplification or sequencing shared by all replicates in a batch).

It is worth re-emphasizing that the results obtained using methyl-IT are not an incremental improvement or slight alteration to those obtained using DMR analysis, rather methyl-IT is providing results so divergent that a completely different interpretation of the underlying biology would be required to explain them. In comparing memory and non-memory plants using DMR analysis, the authors identified 0 DMRs associated with genes CG context (and less than 50 in CHG and CHH contexts). By contrast, comparing the same samples with methyl-IT identified 7,130 genes (roughly a quarter of all the genes in the genome!). This discrepancy is too large to avoid suspicion that methyl-IT is identifying false positives – differences in methylation that are too small to be likely biologically relevant or indeed differences that are due to the inherent inaccuracy of bisulfite-sequencing.

In order to move towards a clearer understanding of these methylation data, I raise the following points/suggestions:

1) The authors must provide some reasonable explanation as to how methyl-IT identifies 7,130 differentially methylated genes between memory and non-memory whereas DMR analysis identifies <50. The differences in methylation at differentially methylated genes shown in Supplementary Figure 11 appear to be approximately 1-2%. The authors must demonstrate how this could possibly be biologically important or underpin the memory phenotype. Methylation differences of this size have never been shown to have an effect on expression or phenotype in any organism, so the burden of proof for making this claim is high.

2) The authors should report the sizes of the methylation differences of DMPs identified by methyl-IT. This should be shown as a distribution, so that reviewers can judge what proportion of DMPs have a methylation difference large enough to be reliably quantified by BS-seq and to be likely biologically relevant. The authors must also show the distribution of coverage at cytosines called as DMPs. As explained above, high coverage is essential in order to accurately call smaller methylation differences.

3) The authors must make sure that PCR duplicates are removed in silico after mapping and prior to quantifying the methylation level of each cytosine. PCR amplification of bisulfite libraries is a further source of inaccuracy due to polymerase preferentially amplifying methylated DNA (see Ji et al 2014, Front. Genet.). The average coverage reported in Supp. Table 3 is about twice as high as that reported by other studies with similar numbers of reads per sample, and as the methods do not mention removing duplicates, I assume this was not performed.

4) The authors must report if replicate samples were processed in batches, either in library construction or sequencing.

5) Given that methyl-IT calls roughly a quarter of all genes in the genome as differentially methylated, the authors must show that association with differentially expressed genes are statistically significant (correcting for multiple hypotheses). With such a large number of differentially methylated genes identified, random association of a subset of these genes with expression changes is almost inevitable.

6) Bisulfite PCR validations of methylation differences should be performed using memory and non-memory plants, rather than memory and WT. Ultimately, if we are to believe that the methylation differences reported are associated with the memory phenotype, these differences should be clearly present between memory and non-memory plants.

7) The authors should provide examples of methylation differences using browser tracks that report raw data rather than methylation differences. It is hard to evaluate whether methylation differences reflect the state of all cytosines at the locus unless raw data is shown.

Other comments and concerns in response to the rebuttal:
1) In my first review I raised a concern that the majority of methylation differences between memory

plants and wild-type were not stably inherited. The authors' rebuttal letter suggested that this was not surprising. However, many studies in the field have shown that the vast majority of methylation patterns are stably inherited over many generations and are even stable over 100-year timescales (e.g. Becker et al 2011, Schmitz et al, 2011, Hagmann et al 2015). The exception is rarely occurring epi-mutations (see Johannes & Schmitz 2019). It is not clear why methylation that is lost in memory lines would be regained in subsequent generations, and why methylation that is gained would not be maintained (particularly in the CG context, which is faithfully maintained by MET1). The authors must present some sort of mechanistic explanation as to why methylation changes associated with memory would not be faithfully inherited.

2) The authors write in the abstract: "First-generation memory versus non-memory full-sib comparison, combined with six-generation inheritance studies, identified gene-associated, heritable methylation repatterning that underpins phenotype". Two claims have been made for which I would argue there is no clear evidence for in the data presented. First, it is not convincingly shown that methylation re-patterning observed is heritable (see point 1) and second, it is not convincingly shown that methylation changes underpin phenotype. In their rebuttal letter, the authors' more cautiously suggest an interesting correlation between methylation and expression, but this is not reflected in the wording of the abstract or the main text.

Reviewer #2 (Remarks to the Author):

I am happy with the response provided by the authors to points 1(gen 6 acts as an outlier), 4 (about the relationship between DMG and DEG), 5 (about the split of methylation per context), 6 (about the silencing of genes involved in DNA methylation pathways in msh1 mutant) and 7 (about the non-memory of CHG methylation).

Point 2. The overlap between the DMRs and DMGs among different generations. The authors have not provided any additional figures or tables in the manuscript to show this overlap (or I could not see them). Only 13 DMR-associated genes are shared between all generation, what are these genes? The authors need to explain this better because it is important to know what are the key genes that truly have a stable memory. Regarding the DMG analysis, do the authors mean that some DMGs appear between gen 1 and gen2 and then disappear and reappear later or that some of the 1st generation DMGs start disappearing with time. This was not clear from the authors response.

3. If the authors used other methods to detect DMRs and the results obtained with these methods overlap only partially, then I would think it would be essential to include this information in the supplementary material at least. This would provide the reader with a better image of potential biases in the analysis. Would the other tools perform more robustly or not compared to DSS? The authors claim "It would certainly be interesting to compare the Methyl-IT output with a number of additional methylation analysis procedures, but that is not our focus in this study." I disagree with the authors. When someone proposes a new method that diverges significantly from others, they need to do the comparisons properly so the reader can evaluate whether the method is robust or not.

Reviewer #3 (Remarks to the Author):

Although the revised manuscript was improved, my major concern is not addressed. In particular, the molecular mechanism underline altered gene networks and differentially methylated loci is not defined. Overall, I admit that the experiments conducted in this study is carefully designed with a lot of data. However, I'd like to point out that the presented analyses are mostly descriptive and

insufficient to give new insights to the biology of Arabidopsis. The conclusions are too generalised with limited novel contribution and mechanistic insight.

Point by point response to comments by reviewers:
We wish to express our gratitude to the reviewers for their thorough and thoughtful review of our work. We understand the concerns of Reviewer 1 and Reviewer 2 regarding our use of a novel methylome analysis methodology in this study. To fully address both reviewers' concerns in a systematic way, we have recently developed a series of publications to elaborate the rationale of our methodology, and to provide detailed instruction about the applications of our methodology. These include:

Sanchez R, Yang X, Maher T, Mackenzie S. Discrimination of DNA Methylation Signal from Background Variation for Clinical Diagnostics. Int J Mol Sci, 2019, 20:5343.

Sanchez R and Mackenzie S. Integrative Network Analysis of Differentially Methylated and Expressed Genes for Biomarker Identification in Leukemia. Scientific Reports (in Press), 2020.

Yang X and Mackenzie SA. Approaches to Whole Genome Methylome Analysis. In: Plant Epigenetics and Epigenomics: Methods and Protocols, Second Edition. C. Spillane and P. McKeown, Eds. Springer Publ. (in press), 2020

Reviewer #1 (Remarks to the Author):

(1) In this revised manuscript, Yang, Sanchez et al have provided multiple additional experiments and data points, but have failed to address the core concern of my previous review (also echoed by other reviewers), which is that the vastly different results obtained by their new analysis pipeline and methodology need to be fully justified in the context of both existing methylation analysis methods and the conclusions and emerging consensus of other studies in the field. Until the methodology of this paper is shown to be accurate, it is very hard to judge the biological significance of the authors' findings.

Response
The methylation analysis approach applied in our manuscript rests on two methods that we consider to be state of the field for analysis of complex data: signal detection and machine learning. Our approach does not involve development of new methodology for data analysis, we have applied well tested analytical approaches. The details for implementation of signal detection and machine learning in Methyl-IT are covered in:

1) Sanchez R, Yang X, Maher T, Mackenzie S. Discrimination of DNA Methylation Signal from Background Variation for Clinical Diagnostics. Int J Mol Sci, 2019, 20:5343.

In this report, we elaborate on the rationale for the application of these methods to methylation data. Simulation studies and analyses of publicly available methylome datasets in human

demonstrate the robustness and feasibility of the approach. We show that a signal detection-based approach provides unbiased estimation of methylation signal that considers the natural background variation in the control population. Signal detection relies on knowledge of the probability distribution of background noise in a system, which can be inferred from the experimental datasets, control and treatment. We also show that Methyl-IT addresses not only identification of methylation signal (DMPs), but whether these statistically significant changes occur with high probability (under fixed experimental conditions) in only the treatment group. Identification of treatment-induced DMPs is not a statistical problem, but a classification or machine learning problem. Conventional methods are not designed or adequate to solve this binary classification problem.

2) Sanchez R and Mackenzie S. Integrative Network Analysis of Differentially Methylated and Expressed Genes for Biomarker Identification in Leukemia. Scientific Reports (in Press), 2020.

In this paper, we describe the integration of Methyl-IT results with network analysis (preprint version available at https://www.biorxiv.org/content/10.1101/658948v1). We show that a signal detection-machine learning approach to methylation analysis of whole genome bisulfite sequencing data permits a high level of methylation signal resolution in cancer-associated genes and pathways. Results obtained with our approach support its application for identification of reliable and stable biomarkers. Were we to be identifying false positives, this would not be feasible.

3) Yang X and Mackenzie SA Mackenzie SA. Approaches to whole genome methylome analysis in plants. Methods in Molecular Biology (in press) 2020.

This chapter describes numerous aspects of whole genome bisulfite sequence data that must be contemplated as well as the various steps of methylome data analysis that can impact biological interpretation of the final output. We point out three major challenges encountered by the current conventional methodologies, 1. The need to distinguish stochastic spontaneous variation within the methylome from treatment-associated signal. 2. The arbitrary nature of data filtering or subjective user settings, given the difficulty of optimizing parameters for particular experiments: settings that are too stringent cause loss of information, while overly relaxed settings cause unacceptable levels of false positives. 3. The nature of methylation heterogeneity: DNA methylation levels vary considerably in samples experiencing changes during development or in response to environmental change, and these effects can show measurable spatio-temporal differences. We also elaborate on why a signal detection and machine learning approach helps to address these challenges.

(2) I shall therefore focus the majority of my review on questioning the authors' analysis methodology, beginning with a defense of existing DMR calling methods and why they were developed and adopted by most in the field in the first place. Bisulfite-sequencing estimates the methylation level of individual cytosines by counting the number reads covering each cytosine in the genome, and comparing the number of converted (T) and unconverted (C) reads for each position. A cytosine covered by 10 converted and 10 unconverted reads would therefore have a methylation level of 50% across all cells in the sample. It is important to note that bisulfite-sequencing therefore has a considerable error in how accurately the "true" methylation level can be portrayed, which is exacerbated at lower read depths. For example, if a cytosine were covered by 2 converted and 4 unconverted reads in one sample (67% methylation level calculated), and 4 converted and 2 unconverted reads in a second sample (33% methylation level calculated), the two samples would display a methylation difference of 33%. However, statistically it is highly likely that these two samples have the same true methylation level, and that the sampling of reads at low depth has created a false positive.

Response

Cytosine methylation is a stochastic process that we have earlier argued cannot be reduced to statistics (Sanchez and Mackenzie, 2016, ref in text). Current statistical approaches applied to methylome analysis ignore the molecular biophysics of the methylation process. Methyl-IT is designed to estimate the probability distribution of noise (plus signal), expressed in terms of Hellinger divergences. As we report in Sanchez and Mackenzie (2019), unless DMR calling methods include information on the probability distribution of methylation background variation, their conclusions are biased and can be taken as reliable only at heavily methylated regions such as transposable element sites.

It is worth mentioning that there are numerous reports of an individual methylation site change proving sufficient to prevent binding of a transcription factor to an enhancer DNA region (eg. Sobiak B. , 2019 doi: 10.3390/ijms20040914), or altering gene expression (eg. Cuomo et al. Clin Epigenetics. 2019,11:149). DMR analysis will disregard such a position, but this type of variation can be picked up by the machine learning approach implemented in Methyl-IT.

Regarding the example discussed by the reviewer, Methyl-IT provides a Bayesian estimation of methylation levels, a first step to reduce the negative effect of low coverage (Methods section from Sanchez and Mackenzie, 2019). For each individual, information on the methylation process retrieved from the experimental data is used to infer the probability distribution of methylation levels. The difference of methylation level is termed total variation distance (TVD). In general, depending on the sample, corrected TVD will be significantly lower than 33%. In

addition, Methyl-IT works with two information divergences: TVD and Hellinger divergence (HD). The default function to compute HD includes a correction based on the coverage (total counts) from each sample (Basu et al. Stat Probab Lett, 2010, 80:206–14).

(3) In order to therefore claim that a methylation difference of 10% or lower (such as those reported in Figure 5a) can be accurately measured with statistical confidence, the authors' would therefore need extraordinary read depth. If a conventional statistical test such as the Chi squared were chosen, the read depth of an individual cytosine would need to be >200 to call a methylation difference of 10% with statistical significance. As such read depths are largely unobtainable, various statistical methods have been developed to identify differentially methylation regions (DMRs) with greater confidence. These methods are not perfect, as they can still generate several false positive or negative results across a large genome, depending on stringency. DMR analyses typically enforce that cytosines must have large differences in methylation level (e.g. 35-40% for a CG dinucleotide) and that they pass thresholds of statistical significance using a test such as Fisher's exact test.

Response

Figure 5a shows only a general overview of methylation variation in each of our sample populations based on raw data without any statistical test. This is the start point of our analysis, and we do not claim any statistical or biological significance based on Figure 5a. In this figure, we merely indicate that there are detectable methylation changes occurring in memory lines that may be associated with the memory phenotype and merit further investigation. All results from subsequent analysis omit cytosine sites with methylation differences lower than 10%. The minimum methylation difference cut off used was 20% as stated in the Methods section.

It should be noted that the DMR analyses referenced by the reviewer are based entirely on direct comparison of control and treatment samples without a reference. Comparison of two magnitudes is valid if, and only if, they were measured relative to the same reference, the same origin of coordinates. Yet, this is not done for the conventional methylation analysis process. Each individual from the same population and even each chromosome follows an independent stochastic methylation process. As we show in Sanchez and Mackenzie (2019), DMPs are found in the control population as well and are not accounted for (or subtracted) by conventional methods.

To consider natural variation in the control population with Methyl-IT, methylation levels and information divergences (TVD and HD) are computed with respect to a reference individual (or pool), which serves as the centroid of the control sample or the centroid of an independent set of

individuals from the control population. The probability distributions of TVD and HD are then used in the downstream analyses. This is detailed in our recent publications.

3.1) Importantly, DMRs also identify regions of the genome where multiple cytosines in direct proximity show similar changes in methylation level, as this is much less likely to occur by chance than sampling a single cytosine. The reason for this is also based on biology – changes in the methylation of single cytosines have never been shown to have a biologically relevant effect in vivo, whereas changes to the methylation state of larger regions have been shown to affect expression of some genes and TEs in several species.

Response

We are not in agreement with the reviewer on this point. There are several reports on changes in the methylation of single cytosines that have biologically relevant effects *in vivo*. We point out only a few of the reviews on this topic with several examples described within each:

1. Liyanage VRB, Jarmasz JS, Murugeshan N, Bigio MRD, Rastegar M, Davie JR. DNA Modifications: Function and Applications in Normal and Disease States. Biology (Basel), 2014, 3:670–723
2. Leenen FAD, Muller CP, Turner JD. DNA methylation: conducting the orchestra from exposure to phenotype? Clin Epigenetics, 2016, 8:1–15
3. Tirado-Magallanes R, Rebbani K, Lim R, Pradhan S, Benoukraf T. Whole genome DNA methylation: Beyond gene silencing. Oncotarget, 2017, 8:5629–37
4. Sobiak B, Leśniak W. The Effect of Single CpG Demethylation on the Pattern of DNA-Protein Binding. Int J Mol Sci, 2019, 20

The signal detection-machine learning approach implemented in Methyl-IT permits the estimation of classification probability for each DMP from each individual into two classes, control and treatment DMPs. Table 2 from Sanchez and Mackenzie (2019) shows that in spite of the high natural background variation detected in placental samples (from autistic children), model classifiers that are built in training sets of one group of patients, independently analyzed with respect to control samples, could be applied to predict the entire set of individual DMPs (control and patient) from the other group (cases “G2 pred. G1” and “G1 pred. G2”). Such a level of resolution is simply not attainable by the conventional methylation analysis methods because they are based on statistical tests like Fisher exact test, generalized linear regression, etc. These tests are not designed to confront a classification problem. In this manuscript, the performance of machine learning classification models built for different generations of the memory line are presented in Supplementary Fig. 3b.

3.2) It is therefore troubling that the results of DMR analysis are dismissed by the authors as not sufficiently sensitive, without careful consideration as to why their sensitivity was purposefully developed to be stringent in the first place.

Response

In the manuscript, when mentioned, DMRs refer to the output derived with DSS. Methyl-IT does not disregard DMRs but, rather, includes them. What would be a DMR detected with DSS, or any other approach, should be detected by Methyl-IT as well. For example, Fig. 8 shows a DMR located on the last exon of a DMG locus identified with Methyl-IT. This DMR was also detected with DSS.

The poor DMR detection accomplished with DSS in the memory samples is due to a lack of sensitivity in detection of DMPs that are subsequently used in the DSS pipeline to build DMRs. We discuss this extensively in our recent publications. At each cytosine site, DSS estimates a Wald statistic (group comparison, mathematically equivalent to the Wald test for coefficients of a Beta-regression) and uses its asymptotic approach to a normal distribution to evaluate whether there are statistically significant differences. Simulations suggest that the problem resides with a high frequency of error type II, since even a Fisher exact test proves more sensitive than DSS (Sanchez and Mackenzie, 2019). For small sample sizes, the assumption for Wald test can be easily violated, leading to error type II. DSS identification of DMPs can be emulated with a simple Beta-binomial regression implemented in R. The reviewer can check the low sensitivity and classification performance of DSS on simulation data in Fig 3 of the Sanchez and Mackenzie (2019) publication, and for a patient with leukemia sample in Fig.4 of the same reference.

The Methyl-IT DMR approach is able to detect irregular distribution of methylation signal, hyper- and hypo- methylated (see Fig. 6 of Sanchez and Mackenzie, 2020: https://www.biorxiv.org/content/10.1101/658948v1.full.pdf, which corresponds to Fig. 7 in the final galley version). Traditional DMR-based approaches fail to detect these types of variation.

3.3) From my understanding of the methods outlined in the PloS One methods paper by Sanchez and Mackenzie (although I am not a mathematician), and the information presented in this manuscript, Methyl-IT does not seem to take into account the inherent inaccuracy of bisulfite sequencing as a technology.

Answer

The problems of bisulfite sequencing technology are generally addressed before the application of the Methyl-IT pipeline, e.g., after the deduplication function from Bismark (to remove potential PCR duplicates) and methylation extractor function (additional filtering option) from Bismark. In Methyl-IT, the function *estimateDivergence* can also provide optional parameter

settings that permit an additional control of these issues (see https://genomaths.github.io/MethylIT_HTML_Manual/estimateDivergence.html). This information is published.

The Sanchez and Mackenzie (2016) paper addressed the statistical biophysics of genome-wide methylation changes. The theoretical distributions for the information divergences of methylation levels were deduced, setting the null hypothesis for the application of the Methyl-IT pipeline. Although that report sets the theoretical basis for a new approach to methylation analysis, it was never intended to describe the pipeline implementation details.

3.4) It is commendable that the authors have generated data for several genetically identical replicates, but this can still lead to false positives if replicates were processed in batches (i.e. biases in PCR amplification or sequencing shared by all replicates in a batch).

Answer

We address the issue of false positive signal in Sanchez and Mackenzie, 2019. The signal detection method of Methyl-IT is combined with a machine learning procedure. This combined assessment is expressly designed to discriminate true biological signal from random background noise and random false positives. Methyl-IT is focused not only on the identification of the methylation signal (DMPs), but also on whether these statistically significant changes occur with high probability (under the fixed experimental conditions) only in the treatment group (machine learning). Specific to this study, however, was also the design of experiments that include full-sib memory and non-memory progeny for comparison. The memory and non-memory classes are unambiguously discriminated in our analysis. This would be absolutely infeasible based on data with a high false positive level. The reviewer does not appear to take note of either of these crucial details.

(4) It is worth re-emphasizing that the results obtained using methyl-IT are not an incremental improvement or slight alteration to those obtained using DMR analysis, rather methyl-IT is providing results so divergent that a completely different interpretation of the underlying biology would be required to explain them. In comparing memory and non-memory plants using DMR analysis, the authors identified 0 DMRs associated with genes CG context (and less than 50 in CHG and CHH contexts). By contrast, comparing the same samples with methyl-IT identified 7,130 genes (roughly a quarter of all the genes in the genome!). This discrepancy is too large to avoid suspicion that methyl-IT is identifying false positives – differences in methylation that are too small to be likely biologically

relevant or indeed differences that are due to the inherent inaccuracy of bisulfite-sequencing.

Response

The fact that conventional analysis methods are not able to find DMRs in the memory line relative to non-memory is not an indication of the absence of methylation differences (which are clearly observable in Fig.5 and Supplementary Fig. 2). Such an outcome reflects an algorithmic limitation based on the dogma that only genomic regions with high density DMPs with a difference in average methylation levels "high enough" (>30%) are considered DMRs (see e.g., Gaspar JM, Hart RP. DMRfinder: Efficiently identifying differentially methylated regions from MethylC-seq data. BMC Bioinformatics, 2017, 18). Regions containing a mixture of hyper- and hypo-methylated significant sites and low average methylation levels are common, and there exists no biological/biophysical reason to disregard their effect on the local mechanical properties of the DNA region. We point to several references supporting this view.

Ngo TTM, Yoo J, Dai Q, Zhang Q, He C, Aksimentiev A, Ha T. Effects of cytosine modifications on DNA flexibility and nucleosome mechanical stability. Nat Commun, 2016, 7:10813 1. Ngo TTM, Yoo J, Dai Q, Zhang Q, He C, Aksimentiev A, Ha T. Effects of cytosine modifications on DNA flexibility and nucleosome mechanical stability. Nat Commun, 2016, 7:10813

Osakabe A, Adachi F, Arimura Y, Maehara K, Ohkawa Y, Kurumizaka H. Influence of DNA methylation on positioning and DNA flexibility of nucleosomes with pericentric satellite DNA. Open Biol, 2015, 5: 150128

Severin PMD, Zou X, Gaub HE, Schulten K. Cytosine methylation alters DNA mechanical properties. Nucleic Acids Res, 2011, 39:8740–51

Choy JS, Wei S, Lee JY, Tan S, Chu S, Lee TH. DNA methylation increases nucleosome compaction and rigidity. J Am Chem Soc, 2010, 132:1782–3

For this study, we intentionally relaxed Methyl-IT pipeline constraints to obtain the number of 7,130 DMGs. They include genes with low density DMPs, which normally would be excluded by DMR-finder algorithms. We had three reasons for this approach:

a) Cytosine methylation affects the DNA binding of transcription factors at small DNA sequence motifs, which do not classify as DMRs. See, for example:

   Yin Y et al. Impact of cytosine methylation on DNA binding specificities of human transcription factors. Science (80-), 2017, 356).

b) Cytosine methylation affects alternative splicing and the site-specific positions on exon or intron do not classify for DMRs. See for example:

1. Shayevitch R, Askayo D, Keydar I, Ast G. The importance of DNA methylation of exons on alternative splicing. RNA, 2018, 24:1351–62
2. Maunakea AK, Chepelev I, Cui K, Zhao K. Intragenic DNA methylation modulates alternative splicing by recruiting MeCP2 to promote exon recognition. Cell Res, 2013, 23:1256–69.
3. Wang X, Hu L, Wang X, Li N, Xu C, Gong L, Liu B. DNA Methylation Affects Gene Alternative Splicing in Plants: An Example from Rice. Mol Plant, 2016, 9:305–7

Pathways involved in alternative splicing were detected in other studies of the *msh1* translatome by our group (Beltrán, J. et al. Specialized Plastids Trigger Tissue-Specific Signaling for Systemic Stress Response in Plants. Plant Physiol. 178, 672–683 (2018)).

c) We, instead, applied downstream filtering by using network analyses and transgenerational intersection of DMGs in networks, which produced 373 DMGs for memory phenotype-associated genes (Supplementary Fig. 17).

(5) In order to move towards a clearer understanding of these methylation data, I raise the following points/suggestions:

5.1) The authors must provide some reasonable explanation as to how methyl-IT identifies 7,130 differentially methylated genes between memory and non-memory whereas DMR analysis identifies <50.

Response
We address this point above.

While we appreciate that our approach is novel, the manuscript makes clear that the initial output of ca. 7000 DMGs in the memory sample analysis is interpreted to mean that 7000 gene regions show high probability of undergoing methylation changes in the memory plants that were assayed. It is from these data that we carry out additional steps of verification that serve to distill this number to 954 memory-associated DMGs that are stable transgenerationally, and to identify 373 DMGs that are sufficient to discriminate memory from non-memory. It stands to reason that numerous methylation changes take place that are not carried transgenerationally (as is shown for CG methylation in the same publications referenced by Reviewer 1), and many of the changes detected may have little or no impact on phenotype. The apparent lack of detected biological impact by a detected DMG does not negate its validity as true signal; it merely provides a comprehensive picture of the behavior of methylation variation across the genome, which calls for further studies. The analysis we provide here involves arguably the most robust dataset yet available for investigating “natural” methylome behavior in association with phenotype (memory-nonmemory) and transgenerational stability. Our recent publications on

Methyl-IT make clear, with both simulated and human methylome datasets, that considerable care and explanation goes into each step of our analysis. In contrast to comments by Reviewer 1 implying that the approach we have used is unconventional and likely inappropriate, the implementation of signal detection theory and machine learning is completely consistent with the state of the field for addressing datasets prone to stochasticity, as referenced in our Methyl-IT publications. We would argue that it is not useful here to re-litigate the Methyl-IT procedure following previous rigorous reviews by computational scientists for its publication. All of the issues raised by the reviewers regarding the methodology are directly addressed in the Methyl-IT publications referenced here and in the revised manuscript.

5.2) The differences in methylation at differentially methylated genes shown in Supplementary Figure 11 appear to be approximately 1-2%. The authors must demonstrate how this could possibly be biologically important or underpin the memory phenotype. Methylation differences of this size have never been shown to have an effect on expression or phenotype in any organism, so the burden of proof for making this claim is high.

Response

Supplementary Figure 11 is just an overview of the methylation changes over the 954 heritable DMGs region, each point on a line shown in this figure represents the average methylation level across DMGs in a bin, about 100 bp in length, and many positions don't have any methylation. The figure shows the expected behavior/methylation-patterning up to 2kb upstream of the transcription start site (TSS), and 2kb downstream of the transcription end site (TES).

At a given bin, some DMGs show significant variation in methylation level and others do not. The point of this figure is to show that visibly significant methylation changes in these 954 heritable DMG regions occur. We do not claim that all of the 954 heritable DMGs are associated with phenotype changes.

5.3) The authors should report the sizes of the methylation differences of DMPs identified by methyl-IT. This should be shown as a distribution, so that reviewers can judge what proportion of DMPs have a methylation difference large enough to be reliably quantified by BS-seq and to be likely biologically relevant. The authors must also show the distribution of coverage at cytosines called as DMPs. As explained above, high coverage is essential in order to accurately call smaller methylation differences.

Response

The minimum difference in methylation levels is 20% in our DMPs, as mentioned above. Supplementary Fig. 3b provides the classification performance of DMPs for wildtype vs memory

and memory vs non-memory comparisons. The accuracies, sensitivities and FDRs reported leave no reasonable doubt about the robustness of DMP identification.

5.4) The authors must make sure that PCR duplicates are removed in silico after mapping and prior to quantifying the methylation level of each cytosine. PCR amplification of bisulfite libraries is a further source of inaccuracy due to polymerase preferentially amplifying methylated DNA (see Ji et al 2014, Front. Genet.). The average coverage reported in Supp. Table 3 is about twice as high as that reported by other studies with similar numbers of reads per sample, and as the methods do not mention removing duplicates, I assume this was not performed.

Answer:

All of our methylome data present in this study have been run through PCR duplicate removing procedures, including:

1. deduplicate_bismark function in Bismark, with default parameters.
2. A coverage filter, cytosine sites with more than 500 reads were removed.

We assume these are standardly accepted procedures, with no need for mention in the Methods section, but we have now added these details to the Methods section in the current version of manuscript.

The coverage shown in Suppl. Table 3 is the coverage after mapping but before the filtering procedures. It was our intent to show the raw clean reads coverage rather than the filtered coverage, because different research groups use different filtering procedures. If reviewer still has doubts, we have uploaded both raw data and processed data to the NCBI GEO database.

5.5) The authors must report if replicate samples were processed in batches, either in library construction or sequencing.

Response:

This information is present in our Data Availability section, and even more details are available at the Gene Expression Omnibus database by using the Secure tokens provided for reviewers.

5.6) Given that methyl-IT calls roughly a quarter of all genes in the genome as differentially methylated, the authors must show that association with differentially expressed genes are statistically significant (correcting for multiple hypotheses). With such a large number of differentially methylated genes

identified, random association of a subset of these genes with expression changes is almost inevitable.

Response:
Our report makes clear that the initial output of ca. 7000 DMGs in memory sample analysis can be interpreted to mean that 7000 gene regions show high probability of undergoing methylation changes in the memory plants that were assayed. We do not claim that all of them are associated gene expression. It is from these data that we carry out additional steps of verification that serve to distill this number to 954 memory-associated DMGs that are stable transgenerationally, identify 373 DMGs sufficient to discriminate memory from non-memory, and provide evidence supporting association of methylation changes at these genes with the phenotype (Supplementary Fig. 17).

5.7) Bisulfite PCR validations of methylation differences should be performed using memory and non-memory plants, rather than memory and WT. Ultimately, if we are to believe that the methylation differences reported are associated with the memory phenotype, these differences should be clearly present between memory and non-memory plants.

Response:
The region-specific bisulfite PCR validations that are presented serve as an additional method to confirm the accuracy of our DMP calling procedure. They are not used as a way to confirm association between memory phenotype and specific DMPs for following reasons:

1. Bisulfite PCR results are subject to PCR bias. This is true for region-specific bisulfite PCR as well.
2. Region-specific bisulfite PCR is time consuming, low efficiency, and difficult for large regions and large sample sizes. Whole-genome bisulfite sequencing data are more reliable.
3. As suggested in Supplementary Fig. 17, we speculated that memory phenotype is likely a quantitative effect that may involve numerous DMGs with spatio-temporal specificity. This would be impossible to confirm by region-specific bisulfite PCR.

5.8) The authors should provide examples of methylation differences using browser tracks that report raw data rather than methylation differences. It is hard to

evaluate whether methylation differences reflect the state of all cytosines at the locus unless raw data is shown.

Response:

All regions shown in Fig. 8. And Supplementary Figures 13, 14, 15, 16 and 19 are selected by rigorous procedures, including conventional procedures used in methylome data analysis (deduplication, removal of extreme high coverage reads, removal of low methylation difference cytosine positions), signal detection and machine learning procedures, and confirmation by biological relevance (presence in memory plants). We consider these to be significant and biologically meaningful. All raw data are made available to the reader.

(6) Other comments and concerns in response to the rebuttal:

6.1) In my first review I raised a concern that the majority of methylation differences between memory plants and wild-type were not stably inherited. The authors' rebuttal letter suggested that this was not surprising. However, many studies in the field have shown that the vast majority of methylation patterns are stably inherited over many generations and are even stable over 100-year timescales (e.g. Becker et al 2011, Schmitz et al, 2011, Hagmann et al 2015). The exception is rarely occurring epi-mutations (see Johannes & Schmitz 2019). It is not clear why methylation that is lost in memory lines would be regained in subsequent generations, and why methylation that is gained would not be maintained (particularly in the CG context, which is faithfully maintained by MET1). The authors must present some sort of mechanistic explanation as to why methylation changes associated with memory would not be faithfully inherited.

Response:

It stands to reason that numerous methylation changes take place that are not carried transgenerationally (as is shown for CG methylation in the publications referenced by the reviewer), and many of the changes detected may have little or no impact on phenotype. The apparent lack of detected biological impact by a detected DMG does not negate its validity as true signal. The analysis we provide here involves arguably the most robust dataset yet available for investigating "natural" methylome behavior in association with phenotype (memory-nonmemory) and transgenerational stability. There is an important point to make here, and that is that the concept of "memory" in plants is still emerging as a phenomenon. We cannot conclude from our study that memory is carried fully in the methylome pattern. Rather, it is likely that memory is also associated with RdDM-dependent components and may be reset each generation. This is a study that continues. However, what we do show is that methylation repatterning, as detected by our study and in combination with gene expression analysis, provides greater resolution of the gene pathways associated with memory than gene expression alone.

6.2) The authors write in the abstract: “First-generation memory versus non-memory full-sib comparison, combined with six-generation inheritance studies, identified gene-associated, heritable methylation repatterning that underpins phenotype”. Two claims have been made for which I would argue there is no clear evidence for in the data presented. First, it is not convincingly shown that methylation re-patterning observed is heritable (see point 1) and second, it is not convincingly shown that methylation changes underpin phenotype. In their rebuttal letter, the authors’ more cautiously suggest an interesting correlation between methylation and expression, but this is not reflected in the wording of the abstract or the main text.

Response

We do not agree with this statement by the reviewer, and we have provided substantial evidence to support our conclusion of methylation re-patterning in memory that is heritable and associated with genes influencing phenotype:

Fig. 4. Shows a similar pattern of DMP distribution for all six generations of wt vs memory and memory vs non-memory comparison,
Fig. 6. Shows similar statistically-significant over-enrichment of gene networks for all six generations of wt vs memory and memory vs non-memory comparison.
Fig. 7 , Supplementary Figures 9 and 10 show that significant DMGs identified in non-memory vs memory are heritable and enriched in phenotype-related pathways.
Fig. 8, **Supplementary Figures 14, 15, 16, and 19** show examples of heritable phenotype-related individual genes
Supplementary Fig. 17 shows the methylation pattern of the 945 heritable DMGs identified is sufficient to discriminate the memory plants from WT plants and non-memory plants from WT plants.
To disregard these data without an alternative explanation, or to attribute this to random false positives is not a reasonable or critical evaluation of the data.

Reviewer #2 (Remarks to the Author):

I am happy with the response provided by the authors to points 1(gen 6 acts as an outlier), 4 (about the relationship between DMG and DEG), 5 (about the split of methylation per context), 6 (about the silencing of genes involved in DNA methylation pathways in msh1 mutant) and 7 (about the non-memory of CHG methylation).

Point 2. The overlap between the DMRs and DMGs among different generations. The

authors have not provided any additional figures or tables in the manuscript to show this overlap (or I could not see them). Only 13 DMR-associated genes are shared between all generation, what are these genes? The authors need to explain this better because it is important to know what are the key genes that truly have a stable memory. Regarding the DMG analysis, do the authors mean that some DMGs appear between gen 1 and gen2 and then disappear and reappear later or that some of the 1st generation DMGs start disappearing with time. This was not clear from the authors response.

Response

As we point out above, the initial output of ca. 7000 DMGs in memory sample analysis can be interpreted to mean that 7000 gene regions show high probability of undergoing methylation changes in the memory plants that were assayed. It is from these data that we carry out additional steps of verification that serve to distill this number to 954 memory-associated DMGs that are stable transgenerationally, and to identify 373 DMGs that are sufficient to discriminate memory from non-memory. The 13 DMRs identified by DSS were omitted from further study because there were no meaningful DMR differences identified between memory and non-memory, and the 13 DMRs showed no overlap with the 954 DMGs identified by Methyl-IT. The reason for poor overlap in DMR and DMG data is strong filtering in DSS that removes from the dataset signal that is detected by Methyl-IT.

In the current work, the gene regions considered for methylation analysis to identify DMGs included gene-body plus 2kb upstream and 2kb downstream. A DMR within this region was considered associated to the given gene. DMRs outside of these regions were not considered.

3. If the authors used other methods to detect DMRs and the results obtained with these methods overlap only partially, then I would think it would be essential to include this information in the supplementary material at least. This would provide the reader with a better image of potential biases in the analysis. Would the other tools perform more robustly or not compared to DSS? The authors claim "It would certainly be interesting to compare the Methyl-IT output with a number of additional methylation analysis procedures, but that is not our focus in this study." I disagree with the authors. When someone proposes a new method that diverges significantly from others, they need to do the comparisons properly so the reader can evaluate whether the method is robust or not.

Answer:

To fully address this issue, we developed and published the manuscript, ***Discrimination of DNA Methylation Signal from Background Variation for Clinical Diagnostics. Int. J. Mol. Sci. 2019, 20(21), 5343***, in which we fully explained the foundation of the signal detection and

machine learning approach we used in Methyl-IT and compared it with 3 other approaches commonly used in methylome analysis, **Fisher's exact test** (used by methylKit), **Wald Test** (used by DSS) and **Root-mean-square test** (used by methylpy).

With simulation studies and two available methylome datasets from autism and leukemia patients, we demonstrate that among the four approaches, signal detection and machine learning used in Methyl-IT produces the highest classification performance. Only signal detection and machine learning provided high discriminatory power for the methylation signal induced by disease.

Reviewer #3 (Remarks to the Author):

Although the revised manuscript was improved, my major concern is not addressed. In particular, the molecular mechanism underline altered gene networks and differentially methylated loci is not defined. Overall, I admit that the experiments conducted in this study is carefully designed with a lot of data. However, I'd like to point out that the presented analyses are mostly descriptive and insufficient to give new insights to the biology of Arabidopsis. The conclusions are too generalised with limited novel contribution and mechanistic insight.

Response:

In our view, these opinions could reasonably be leveled at nearly any study that involves genome-level datasets and the analysis of network disruption. However, based on these comments we have re-edited the Discussion to make more clear the salient points of our study that represent important advancements to understanding transgenerational epigenomic memory (about which plant biologists know almost nothing on a genome-wide scale) and about the networks that participate in this phenomenon. To summarize these advancements:

a. We have demonstrated that Arabidopsis, following epigenomic disruptions by MSH1 suppression, can enter a sustained, heritable, nongenetic state that does not undergo reversion. Included with our previous publications on MSH1, it is reasonable to assume that all plants can similarly enter this state.

b. The msh1 memory state involves changes in methylome and gene expression that, with careful scrutiny, reveal 373 genes that, alone, are sufficient to discriminate memory plants from their non-memory full-sib progeny members. This is an important finding from a novel approach.

c. These 373 genes belong to networks for mRNA spliceosome, as well as circadian rhythm, auxin response, and hormone signal transduction. Assembled network figures and extensive literature indicates that the circadian rhythm, auxin response, ethylene, brassinosteroid,

and cytokinin pathways (all significant msh1 signals) are interlinked, and it is reasonable to assume that they undergo co-regulation and cross-talk in the msh1 system.

d. A known key regulator of the interlinking phytohormone-circadian clock networks is HDA6, as referenced in the text. HDA6 influences local histone modifications as well as recruitment of MET1, a methyltransferase that, together with HDA6, has been reported to be involved in site-directed methylation. This is referenced in the text.

e. The centrality-lethality rule is a relatively long-standing (15 yr?) premise of network behavior that states that members of a network that serve as central hubs can be so essential that their disruption is not tolerable, leading to lethality (Coulomb, S., Bauer, M., Bernard, D. and Marsolier-Kergoat, M. C. (2005). Gene essentiality and the topology of protein-interaction networks. Proc. R. Soc. Lond. B. Biol. Sci. 272, 1721-1725; Batada, N. N., Hurst, L. D. and Tyers, M. (2006). Evolutionary and physiological importance of hub proteins. PLoS Comp. Biol. 2, 0748; Almaas E. Biological impacts and context of network theory. J Exp Biol 2007 210: 1548-1558; doi: 10.1242/jeb.003731). These references have now been added to the text. The observation of *msh1/hda6* lethality is, on hindsight, predictable given the evidence of targeted alteration of the phytohormone-circadian clock networks during MSH1 disruption. We have presented evidence of *msh1/hda6* lethality, supporting HDA6 as a central hub to the msh1-altered networks, and have provided evidence of circadian rhythm dysregulation in the msh1 memory line. Outside of this study, we have also confirmed contribution of the auxin response pathway to msh1 phenotype in data that will be included in a subsequent report to maintain continuity to that study.

Reviewers' comments:

Reviewer #1 (Remarks to the Author):

The authors write: "Identification of treatment-induced DMPs is not a statistical problem". I vehemently disagree with this assertion. Due to the costs and technological constraints of short read sequencing, estimating the methylation level of a cytosine in the genome by bisulfite sequencing will always involve a considerable amount of error, particularly at sites with lower coverage, as there are only a limited number of data points for any given cytosine (I illustrated this with a simple example in my previous review). Due to the fact that bisulfite sequencing assays millions of sites across the genome, a strict consideration of multiple hypothesis testing and statistical confidence for any given depth is crucial when identifying DMPs/DMCs between wild-type and control groups. It is worth noting that this problem is one that has troubled the field for a long time, and there are a huge number different methods continually published to try and improve our ability to detect methylation differences. For example, Shafi et al - Briefings in Bioinformatics, 2018 discuss 22 different differential methylation analysis methods used by the field. Since then, there have been yet more methods, such as Catoni et al - Nucleic Acids Research, 2018, and Srivastava et al – BMC Bioinformatics, 2019, which also include machine learning to help optimize how to place DMPs into regions. These challenges are an inherent accuracy of bisulfite sequencing as a technology and are to an extent, unfixable, which is partly why so many different approaches have been attempted.

These limitations cannot truly be fixed in silico as the authors suggest, and any approaches that claim to circumvent the problem of read depth and the inherent inaccuracy of bisulfite sequencing will cause eyebrows to be raised. To the authors' credit, sequencing multiple replicates is extremely valuable in confidently identifying DMPs, and as I mentioned in my last review this is a strength of the manuscript. However, I still believe the authors would require far greater sequencing depth to confidently support some of the conclusions they are making.

I can understand the authors' frustration, as I have been very skeptical of the claims made on the back of the Methyl-IT analysis. However, while I agree that a full re-litigation of the method may be an inefficient time-sink for all involved, I still strongly believe that the authors' need to provide much greater room for doubt in their interpretation of conclusions drawn from methyl-IT, a clear-cut explanation to the reader as to why methyl-IT provides such drastically different results to other methods and crucially, validation of some differentially methylated genes (in memory versus non-memory) by bisulfite PCR. The authors' criticisms of bisulfite PCR are not sufficient to dismiss it as an additionally validating the biologically important findings. Bisulfite sequencing also suffers from PCR amplification bias (Li et al, Front. Genet. 2014) and bisulfite PCR would permit authors to validate a few of the methylation differences present in their memory line plants at much higher depth.

The authors' have put a lot of time and effort into responding to my points in my first two reviews, but I do not see a clear intent to improve the manuscript and make the data and conclusions more accurate and easier to interpret for the reader on the basis of reviewer's concerns. I have made a number of suggestions already, such as showing the authors a distribution of DMP methylation differences (e.g. what proportion of DMPs have high/low differences in methylation levels?), showing readers the number of DMPs for each DMG (again, a frequency distribution would be most informative) and alerting the reader to whether batch effects could be possible (were all the libraries made at the same time and multi-plexed on the same flow cell?). I hope that the reviewers might consider implementing some of these suggestions.

I am also concerned that at several points in the first two rounds of review I have pressed the authors for further justification to certain claims, and the authors have responded by walking-back some points, or claiming that certain results are not intended to represent biological significance. However, these milder interpretations have not been updated in the manuscript or made clear to the reader. For example, if the methylation differences in Fig. 5a are not intended to represent biologically significant

differences, why show them?

To provide two more examples in detail:

1) The authors' state: "Our report makes clear that the initial output of ca. 7000 DMGs in memory sample analysis can be interpreted to mean that 7000 gene regions show high probability of undergoing methylation changes in the memory plants that were assayed" – I disagree that the report makes this clear to the reader, and the authors' should be providing results that are as close as possible to robust, replicable biologically significant findings. If only a much smaller subset of regions are thought to heritably contribute to phenotype, why present this larger, less certain result at all?

2) The abstract states: "First-generation memory versus non-memory full-sib comparison, combined with six-generation inheritance studies, identified gene-associated, heritable methylation repatterning that underpins phenotype" – I reiterate from my previous review that heritable methylation patterns have not been shown to underpin phenotype. When I pressed the authors on this in my first review, they responded that the association between DMPs and gene expression were an interesting correlation, but this is not reflected in what is presented to the reader.

One last point: I acknowledge that there is certainly some evidence that methylation of single cytosines can be biologically impactful, and I think that this is likely a debate that will continue for a long time in the field. I therefore see value in publishing differences in the methylation of single cytosines and letting the broader readership form their own view. However, I do think it needs to made very clear to the reader how many cytosines are typically differentially methylated in the DMGs identified by the analysis. If (for example) 50% of DMGs were classified based on a single differentially methylated position, the readers should be informed of this so that they can make up their own minds. I only grasped that some of methylation differences were at the single cytosine level from looking at the supplemental figures, and this is something that should be clearly presented up front to the reader.

Reviewer #2 (Remarks to the Author):

Response to point 2.

I requested a heatmap showing the overlap between DMRs/DMGs in different generation. The authors claim that that there are 7000 DMGs but only 954 are stable. It should be straightforward to produce a heatmap showing that overlap between the DMRs/DMGs in different generations.

Furthermore, they claim that none of the 13 DMRs identified by DSS overlap with the 954 DMGs identified by Methyl-IT and subsequent filtering and justify that this because of the stringency of DSS. If DSS is indeed more stringent, then it would capture the very strong stable DMRs, but why does Methyl-IT misses these really strong DMRs. This makes me worried about the claim that this is a better tool, it if cannot even detect the strong DMRs detected by other less sensitive tools.

Response to point 3.

I appreciate that the authors published the tool in a different publication. However, if they have done this analysis on this dataset with other tools, I fail to understand why they do not want to include it (or show it to the readers) so we can make our mind about it. In the paper mentioned by the authors, the analysis is done in humans, where there is no transgenerational inheritance due to erasure of methylation in early embryo development. The worrying thing about this is that methylation patterns and mechanisms can differ significantly between plants and mammals and I have not seen any comparison between the new tool and the existing ones in plants. I am not trying to be difficult, but I am worried about the validity of the results. In the paper suggested by the authors, their new method

seems to recover twice more DMPs compared to DSS and one third more compared to methylKit (figure 4; Sanchez et al., 2019), which is something I would believe as being an improvement in methodology. In this paper they seem to identify approximately 10 times more DMRs/DMGs with their new method compared to DSS and manage not to recover any of the DSS DMRs, which is worrying.

Yang et al. Response to reviewers:

We appreciate the time and attention that reviewers have given to our manuscript. We have introduced several significant changes to the manuscript, listed here. In addition, we reply to each reviewer comment point-by-point below.

1. To fully address the concerns expressed by Reviewer 2, we have replaced all of the DMR-related data from the previous version with a direct comparison of classification performance for DMPs obtained by multiple conventional methodologies, including Fisher's exact test (used in methylKit), Wald Test (used in DSS) and Root-mean-square test (used in methylpy). We also carried out such analysis in Sanchez et al., 2019 with simulation data and human methylome data. Results from the current comparison are presented in Supplemental Fig. 3 to provide the reader with definitive rationale for selection of Methyl-IT in our present analysis.

2. As requested by Reviewer 1, we have now included additional BS-PCR confirmation of our methylome data signal, comparing Generation 1 memory vs non-memory samples. Results are presented as Supplementary Figure 15b. We purposely selected, for this figure, a region that was also compared for memory and wild type. Supplementary Figures 15b and c now show that hypermethylation DMPs exist in the memory state when compared to either non-memory or wild type plants within the *XTH16* gene region (identified as a DMG in our genome-wide analysis), confirming DMP calling accuracy for the non-memory vs memory comparison (first generation) and wild type vs memory (sixth generation). Related comments are added to the Results section and are marked.

3. As requested by Reviewer 1, we have made a density plot of DMP methylation level difference and of DMP number in DMGs, presented as Supplementary Figure 5. Supplementary Fig. 5a density plot of DMP methylation level difference shows a majority of DMPs in every generation with methylation level difference greater than 0.5 in non-memory vs memory and wild type vs memory comparisons (hyper or hypo). Supplementary Fig. 5b Density plot of DMP number in DMGs shows memory DMGs from all six generations contain substantial DMPs, with the average DMP number in DMGs of each memory individual 25.21 for Gen1 NM vs MM, 22.07 (Gen 2), 22.63 (Gen 3),22.89 (Gen 4),21.73 (Gen 5) and 22.05 (Gen 6) for WT vs MM. Related comments are added to the Results section Page 8 Line 302 to 306.

4. We have removed Fig. 5a and related comments as suggested.

The rationale for making all of these changes can be found in our point-by-point response to reviewer comments below:

Reviewer #1 (Remarks to the Author):

The authors write: "Identification of treatment-induced DMPs is not a statistical problem". I vehemently disagree with this assertion. Due to the costs and technological constraints of short read sequencing, estimating the methylation level of a cytosine in the genome by bisulfite sequencing will always involve a considerable amount of error, particularly at sites with lower coverage, as there are only a limited number of data points for any given cytosine (I illustrated this with a simple example in my previous review). Due to the fact that bisulfite sequencing assays millions of sites across the

genome, a strict consideration of multiple hypothesis testing and statistical confidence for any given depth is crucial when identifying DMPs/DMCs between wild-type and control groups. It is worth noting that this problem is one that has troubled the field for a long time, and there are a huge number different methods continually published to try and improve our ability to detect methylation differences. For example, Shafi et al - Briefings in Bioinformatics, 2018 discuss 22 different differential methylation analysis methods used by the field. Since then, there have been yet more methods, such as Catoni et al - Nucleic Acids Research, 2018, and Srivastava et al – BMC Bioinformatics, 2019, which also include machine learning to help optimize how to place DMPs into regions. These challenges are an inherent accuracy of bisulfite sequencing as a technology and are to an extent, unfixable, which is partly why so many different approaches have been attempted.

Response: We stated in our previous response that we do not consider discrimination of treatment-associated DMPs to be so much a statistical problem as a classification problem. We make this statement to clarify our efforts to implement signal detection and machine-learning. We do not imply beyond this point. As you will see in the below citation, this is increasingly a sentiment expressed by others as well.

For example, in *Martínez-Camblor et al.(The role of the p-value in the multitesting problem. J Appl Stat, 2019:1–14),* the authors highlight that the original p-values resulting from a given test must be used as “markers”, and they proposed estimation of an optimal p-value cutpoint from the ROC curve. In other words, they transform the multitesting problem into a binary classification problem. Additionally, *Srivastava A, et al. (HOME: a histogram based machine learning approach for effective identification of differentially methylated regions. BMC Bioinformatics, 2019, 20:253)* also confronts methylation analysis as a classification problem. Unfortunately, in this latter case, the authors did not take into account the natural variation of the control population, but it does incorporate more powerful discrimination.

These limitations cannot truly be fixed in silico as the authors suggest, and any approaches that claim to circumvent the problem of read depth and the inherent inaccuracy of bisulfite sequencing will cause eyebrows to be raised. To the authors’ credit, sequencing multiple replicates is extremely valuable in confidently identifying DMPs, and as I mentioned in my last review this is a strength of the manuscript. However, I still believe the authors would require far greater sequencing depth to confidently support some of the conclusions they are making.

Response: We have provided more information on DMP classification as described above.

I can understand the authors’ frustration, as I have been very skeptical of the claims made on the back of the Methyl-IT analysis. However, while I agree that a full re-litigation of the method may be an inefficient time-sink for all involved, I still strongly believe that the authors’ need to provide much greater room for doubt in their interpretation of conclusions drawn from methyl-IT, a clear-cut explanation to the reader as to why methyl-IT provides such drastically different results to other methods and crucially, validation of some differentially methylated genes (in memory versus non-memory) by bisulfite PCR. The authors’ criticisms of bisulfite PCR are not sufficient to dismiss it as an additionally validating the biologically important findings. Bisulfite sequencing also suffers from PCR amplification bias (Li et al, Front. Genet. 2014) and bisulfite PCR would permit authors to validate a few of the methylation differences present in their memory line plants at much higher depth.

Response: As suggested, we have added an additional experiment for BS-PCR confirmation (memory versus non-memory), with results in Supplementary Fig. 15b. Related comments are added to the Results section **Page 13 Line 490 to 492**.

The authors’ have put a lot of time and effort into responding to my points in my first two reviews, but I do not see a clear intent to improve the manuscript and make the data and conclusions more

accurate and easier to interpret for the reader on the basis of reviewer's concerns. I have made a number of suggestions already, such as showing the authors a distribution of DMP methylation differences (e.g. what proportion of DMPs have high/low differences in methylation levels?), showing readers the number of DMPs for each DMG (again, a frequency distribution would be most informative) and alerting the reader to whether batch effects could be possible (were all the libraries made at the same time and multi-plexed on the same flow cell?). I hope that the reviewers might consider implementing some of these suggestions.

Response: As suggested, we have incorporated a density plot of DMP methylation level difference and of DMP numbers per DMG, now presented in Supplementary Fig. 5. Related comments are included in the Results section **Page 8 Line 302 to 303**.

All 65 sample libraries were processed and loaded onto 4 different HiSeq X-ten flow cells, with a majority of them (38/65) on the same cell. The whole process was carried out under a well-controlled and stringent protocol. Based on the output reads number from each sample (shown in Supplementary Table 1), variation among samples is very low, indicating that batch effect within our samples is negligible. We have added this additional information to the Methods section **Page 26 Line 856 to 860**.

I am also concerned that at several points in the first two rounds of review I have pressed the authors for further justification to certain claims, and the authors have responded by walking-back some points, or claiming that certain results are not intended to represent biological significance. However, these milder interpretations have not been updated in the manuscript or made clear to the reader. For example, if the methylation differences in Fig. 5a are not intended to represent biologically significant differences, why show them?

Response: Our intention with Figure 5a was to provide evidence of memory/non-memory differences without reliance on the Methyl-IT procedure, which is considered to be a novel procedure by the plant community. We have now removed Figure 5a and will rely on the remainder of this figure to make this point.

To provide two more examples in detail:
1) The authors' state: "Our report makes clear that the initial output of ca. 7000 DMGs in memory sample analysis can be interpreted to mean that 7000 gene regions show high probability of undergoing methylation changes in the memory plants that were assayed" – I disagree that the report makes this clear to the reader, and the authors' should be providing results that are as close as possible to robust, replicable biologically significant findings. If only a much smaller subset of regions are thought to heritably contribute to phenotype, why present this larger, less certain result at all?

Response: We have rewritten this section for clarity and added the comment: "Designation of DMGs indicates that a substantial number of DMPs with significant methylation difference (Supplementary Fig.5) are present within the gene, which is considered to display high probability of undergoing methylation change in the memory plants that were assayed. We do not expect all DMGs reported to necessarily be associated with the memory phenotype" (Page 8 Line 302 to Page 9 line 306).

There are several reasons that we use a larger set of DMGs at the start point of our analysis:

1. As alluded to by the reviewer, we have a larger population sample size and better data quality than most published methylome datasets (6 generations, 65 samples, 4 Gb reads per sample).

2. We have the ability to further screen the DMG datasets using two biologically meaningful filters (network enrichment analysis and heritability)

3. We are able to obtain large DEG datasets in Gen1 WT vs MM (4509) and Gen5 WT vs MM, so that a similarly sized DMG dataset facilitates identification of potential pathway associations between DMG and DEG data.

To more fully address the reviewer's concern, we have also conducted network enrichment analysis with a dataset of 2637 DMG for NM vs MM. This dataset was obtained from more stringent initial filtering. Results show a very similar enriched network profile as identified from the 7130 DMG dataset (intermediate filter conditions). While the more highly filtered dataset also identified *circadian rhythm, response to auxin* and *phytohormone signal transduction pathways*, we obtained fewer individual genes in each pathway, thus reducing pathway resolution (now included in Supplementary Fig. 6). These observations imply that the enriched network profile identified is robust and biological meaningful regardless of whether we use the full 7130 dataset or filtered 2637 dataset. For better resolution of DMG network intersection with gene expression changes, we have retained the DMG dataset identified with intermediate filtering. Related comments have been added to the text (**Page 8 Line 311 to Page 9 Line 335**).

2) The abstract states: "First-generation memory versus non-memory full-sib comparison, combined with six-generation inheritance studies, identified gene-associated, heritable methylation repatterning that underpins phenotype" – I reiterate from my previous review that heritable methylation patterns have not been shown to underpin phenotype. When I pressed the authors on this in my first review, they responded that the association between DMPs and gene expression were an interesting correlation, but this is not reflected in what is presented to the reader.

Response: Our report identifies, via methylome and gene expression analysis, gene pathways that discriminate between memory and nonmemory phenotypes. In fact, our study identifies 373 loci with methylation change that is sufficient to discriminate these phenotypes. These pathways are similarly represented for six generations of memory. To our knowledge, this is the first study of its kind to utilize progeny from a single parent to discriminate phenotype with underlying methylation and gene expression pathways at such resolution. We do not consider this to be merely interesting correlation, but evidence that the phenotypic plasticity observed following msh1 mutation can be traced to specific intersecting pathways. We have attempted to clarify these points in the text.

One last point: I acknowledge that there is certainly some evidence that methylation of single cytosines can be biologically impactful, and I think that this is likely a debate that will continue for a long time in the field. I therefore see value in publishing differences in the methylation of single cytosines and letting the broader readership form their own view. However, I do think it needs to made very clear to the reader how many cytosines are typically differentially methylated in the DMGs identified by the analysis. If (for example) 50% of DMGs were classified based on a single differentially methylated position, the readers should be informed of this so that they can make up their own minds. I only grasped that some of methylation differences were at the single cytosine level from looking at

the supplemental figures, and this is something that should be clearly presented up front to the reader.

Response: We now address this directly in Supplementary Fig. 5b with a density plot of DMP number in DMGs, showing that each memory DMG from all six generations contains substantial DMP numbers, with average DMP number per DMG in each memory individual at 25.21 for Gen1 NM vs MM, 22.07 (Gen 2), 22.63 (Gen 3),22.89 (Gen 4),21.73 (Gen 5) and 22.05 (Gen 6) for WT vs MM. Related comments are added to the Results section **Page 8 Line 302 to 303**.

Reviewer #2 (Remarks to the Author):

Response to point 2.

I requested a heatmap showing the overlap between DMRs/DMGs in different generation. The authors claim that that there are 7000 DMGs but only 954 are stable. It should be straightforward to produce a heatmap showing that overlap between the DMRs/DMGs in different generations.

Furthermore, they claim that none of the 13 DMRs identified by DSS overlap with the 954 DMGs identified by Methyl-IT and subsequent filtering and justify that this because of the stringency of DSS. If DSS is indeed more stringent, then it would capture the very strong stable DMRs, but why does Methyl-IT misses these really strong DMRs. This makes me worried about the claim that this is a better tool, it if cannot even detect the strong DMRs detected by other less sensitive tools.

Response: The reason why we have not provided such a figure is that DMR-associated genes and DMGs are two completely different concepts defined by two distinct sets of parameters; the two datasets are not truly comparable, representing two different approaches to dataset analysis. For the reviewer's information, we include below the heatmap as requested:

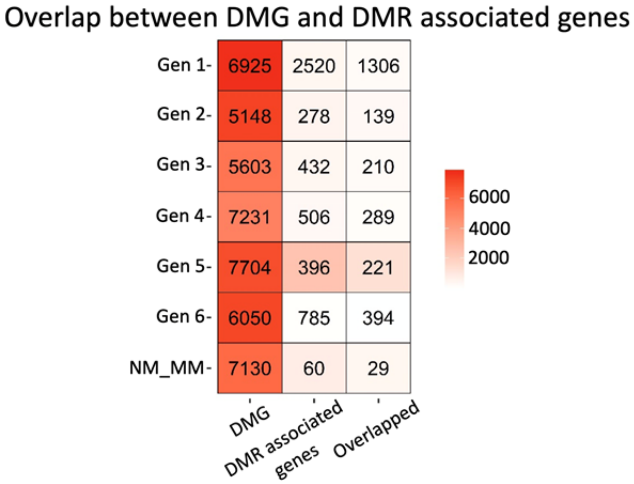

As one sees from the heatmap, the percentage of DMR-associated genes that overlap with DMGs is 48.3%, 51.8%, 50%, 48.6%, 57.1%, 55.8%, and 50.2% for gen1 to gen 6 WT vs MM and NM vs MM, respectively. A DMR (within treatment sample) is a region that displays significant overall methylation difference relative to its control sample. DMRs vary from 50bp to kb in size, and its associated gene is a gene within 1kb in our analysis. The associated gene itself may not show significant change. The reason that many DMR-associated genes do not define as DMGs is that they lack sufficient DMPs within the gene. As an example, a DMR-associated gene in memory/non-memory comparison is AT1G23330. The gene locates on chromosome 1 at

position (8278554:8283421), displaying one DMR with a length of 85bp (8282978:8283060) but only 9 DMPs. The size of AT1G23330 is 4868bp, so the density of DMPs over the gene will be 0.0018, which filters out in the Methyl-IT procedure. For Methyl-IT, DMP density cut off is at least 2.5 DMP/kb (> 0.0025).

Response to point 3.
I appreciate that the authors published the tool in a different publication. However, if they have done this analysis on this dataset with other tools, I fail to understand why they do not want to include it (or show it to the readers) so we can make our mind about it. In the paper mentioned by the authors, the analysis is done in humans, where there is no transgenerational inheritance due to erasure of methylation in early embryo development. The worrying thing about this is that methylation patterns and mechanisms can differ significantly between plants and mammals and I have not seen any comparison between the new tool and the existing ones in plants. I am not trying to be difficult, but I am worried about the validity of the results. In the paper suggested by the authors, their new method seems to recover twice more DMPs compared to DSS and one third more compared to methylKit (figure 4; Sanchez et al., 2019), which is something I would believe as being an improvement in methodology. In this paper they seem to identify approximately 10 times more DMRs/DMGs with their new method compared to DSS and manage not to recover any of the DSS DMRs, which is worrying.

Response: It is important to note that the DMP numbers we present in Figure 4 of Sanchez et al. (2019) are DMP numbers from just one chromosome. This outcome is not comparable to DMG or DMR numbers presented in this manuscript.

In Figure 3 of Sanchez et al. (2019), we also present simulation data, so our analysis is not simply relevant to human data studies.

Our inclusion of DSS and Methyl-IT data in the previous version of this manuscript may have caused confusion. As indicated by the reviewer and presented in the previous Methyl-IT publication (Sanchez et al., 2019), a more comprehensive way to compare different methylome analysis methods is to compare at the DMP level, with most DMPs from different methodologies defined similarly. We have now replaced the DMR-related data in the previous version with a direct comparison of classification performance for DMPs obtained by different methodologies, including Fisher's exact test (used by methylKit), Wald Test (used by DSS) and Root-mean-square test (used by methylpy), similar to what was done in Sanchez et al. (2019) for simulation and human data. Results are now presented in Supplementary Figure 3. Consistent with our previous observations from simulation and human data, Supplementary Figure 3a, 3b show that the signal detection-machine learning procedure shows best overall DMP calling performance among the four methods (second highest number of DMPs identified, highest in accuracy, sensitivity, specificity and lowest in FDR). We have edited text in the manuscript at Page 6 line 143 - Page 7 line 275.

REVIEWERS' COMMENTS:

Reviewer #2 (Remarks to the Author):

The authors have performed the requested analysis for both reviewer's 1 and 2 comments. Despite the authors performing the recommended analysis, I cannot support the publication of this article in Nature Communications.
